# Supplementary figures and images for: A novel traffic optimization method using GRU based deep neural network for the IoV system (part 2 of 2)
Source: PeerJ Comput Sci. 2023 Jun 6;9:e1411. doi: 10.7717/peerj-cs.1411 (PMC10280423; doi:10.7717/peerj-cs.1411)

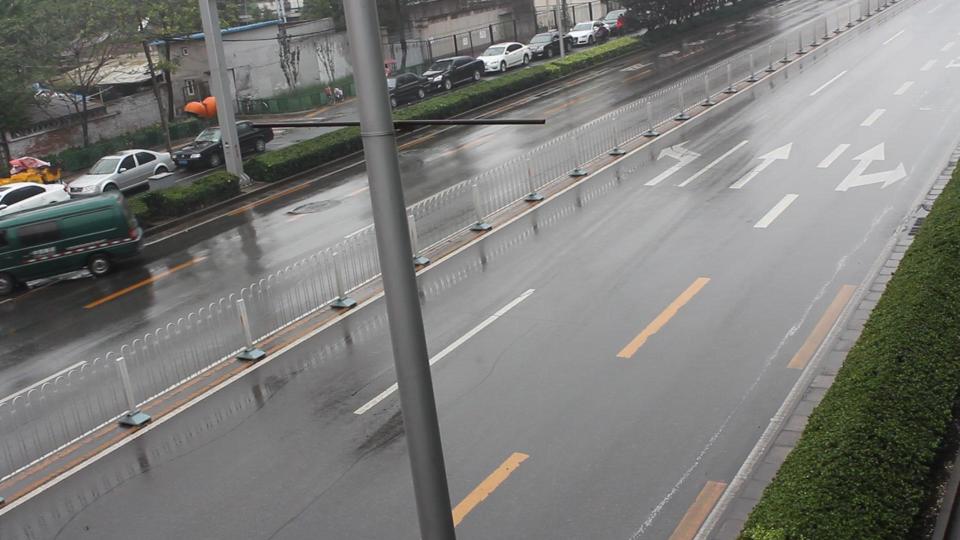

Supplement: Data S1 [file peerj-cs-09-1411-s001.zip › dataset/MVI_63544_img00531.jpg]

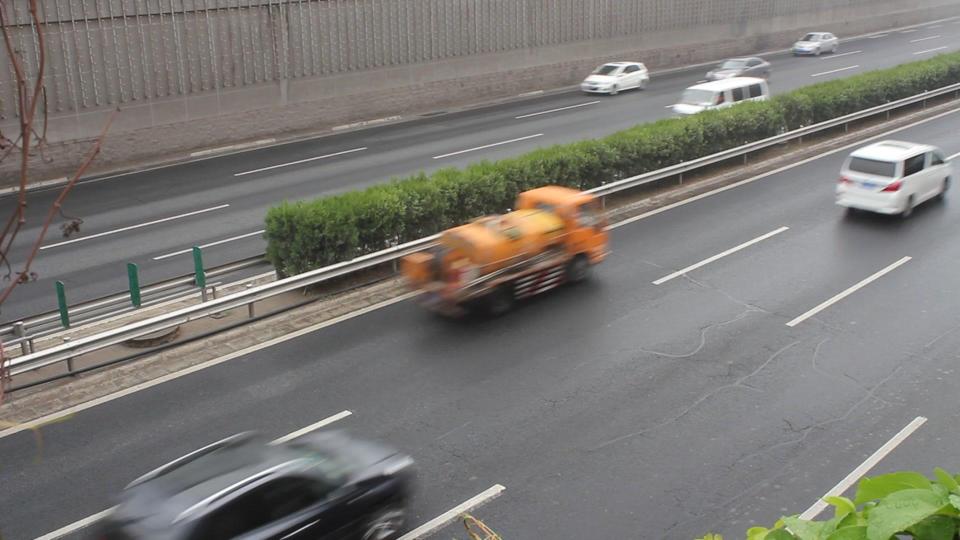

Supplement: Data S1 [file peerj-cs-09-1411-s001.zip › dataset/MVI_63562_img00878.jpg]

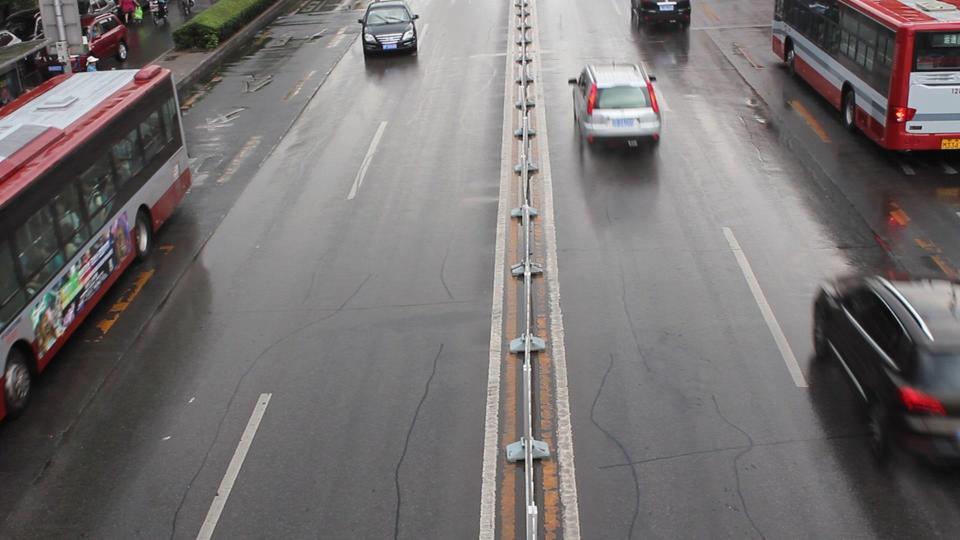

Supplement: Data S1 [file peerj-cs-09-1411-s001.zip › dataset/MVI_63525_img00886.jpg]

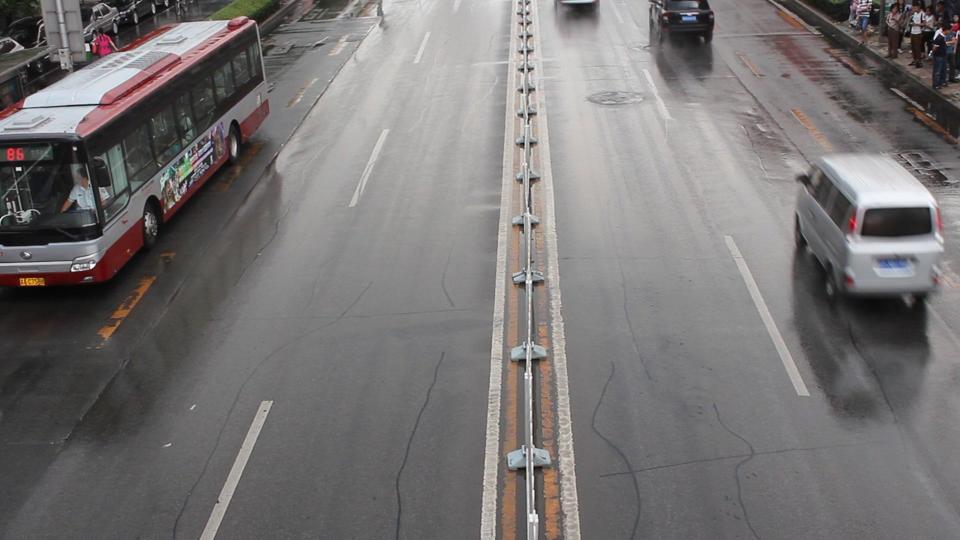

Supplement: Data S1 [file peerj-cs-09-1411-s001.zip › dataset/MVI_63525_img00676.jpg]

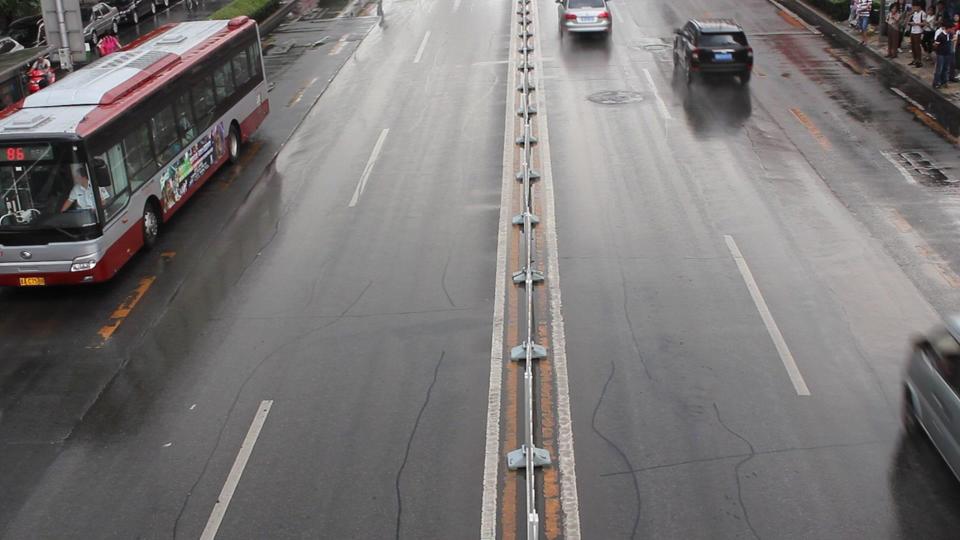

Supplement: Data S1 [file peerj-cs-09-1411-s001.zip › dataset/MVI_63525_img00662.jpg]

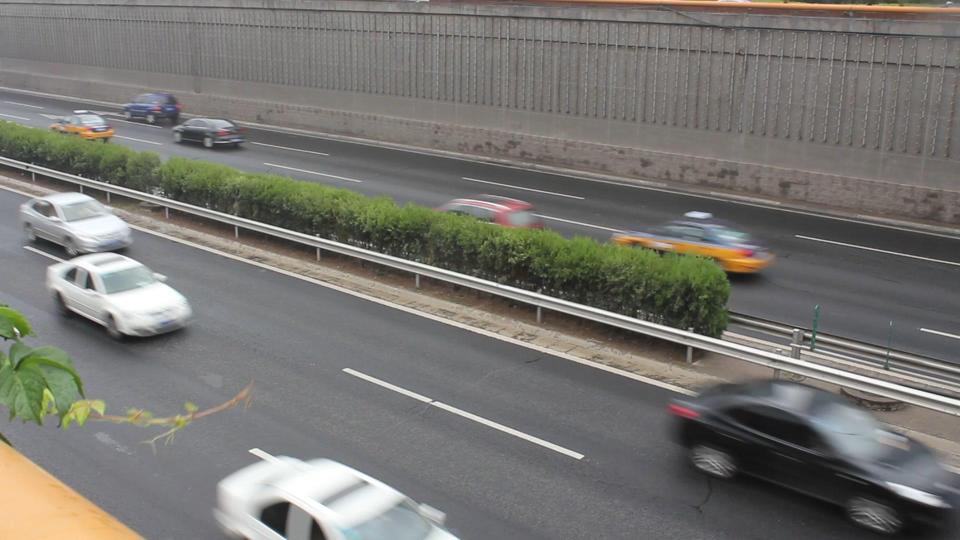

Supplement: Data S1 [file peerj-cs-09-1411-s001.zip › dataset/MVI_63554_img00852.jpg]

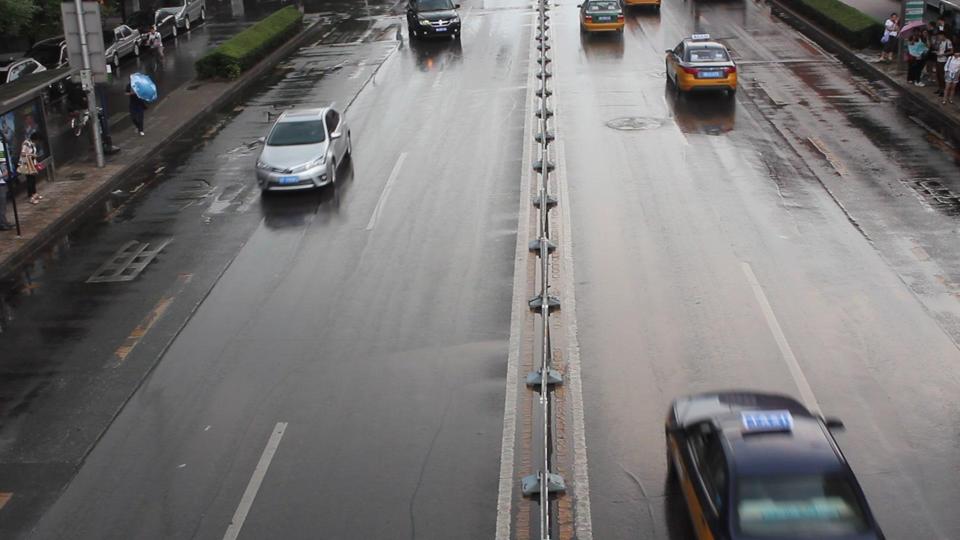

Supplement: Data S1 [file peerj-cs-09-1411-s001.zip › dataset/MVI_63521_img01951.jpg]

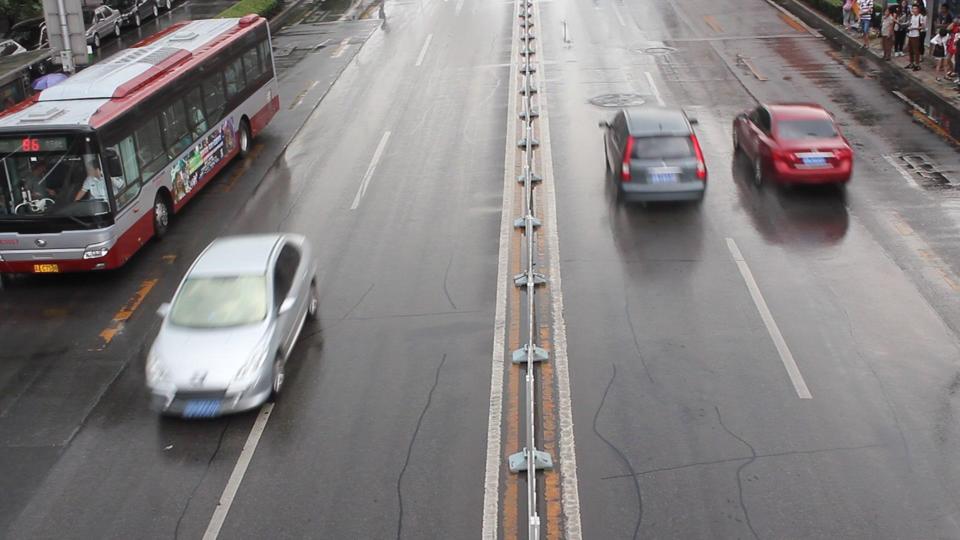

Supplement: Data S1 [file peerj-cs-09-1411-s001.zip › dataset/MVI_63525_img00312.jpg]

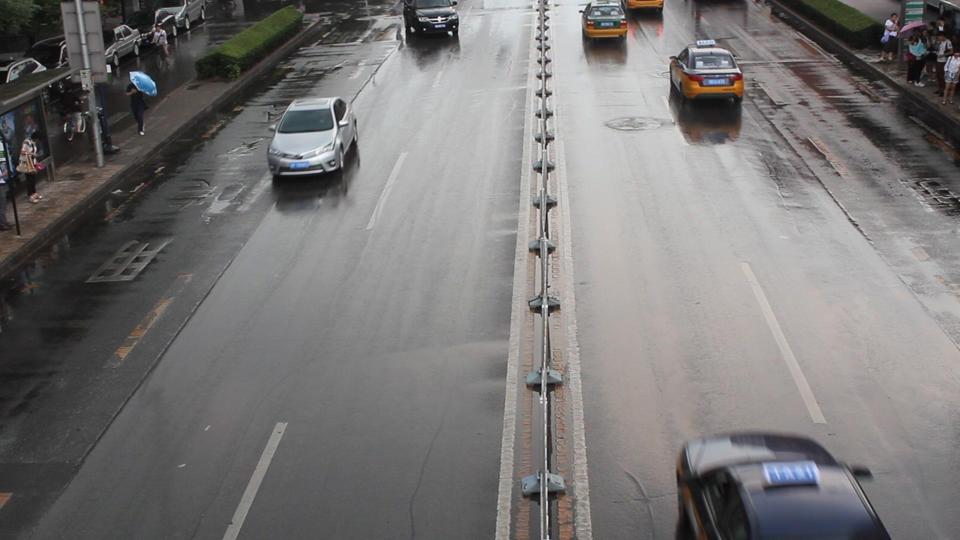

Supplement: Data S1 [file peerj-cs-09-1411-s001.zip › dataset/MVI_63521_img01947.jpg]

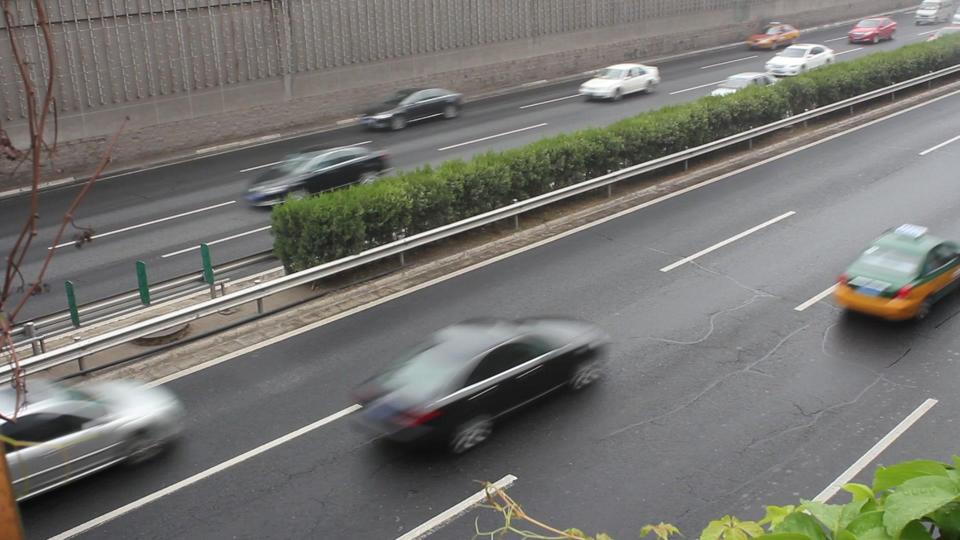

Supplement: Data S1 [file peerj-cs-09-1411-s001.zip › dataset/MVI_63562_img00280 - σë»μ£1⁄4.jpg]

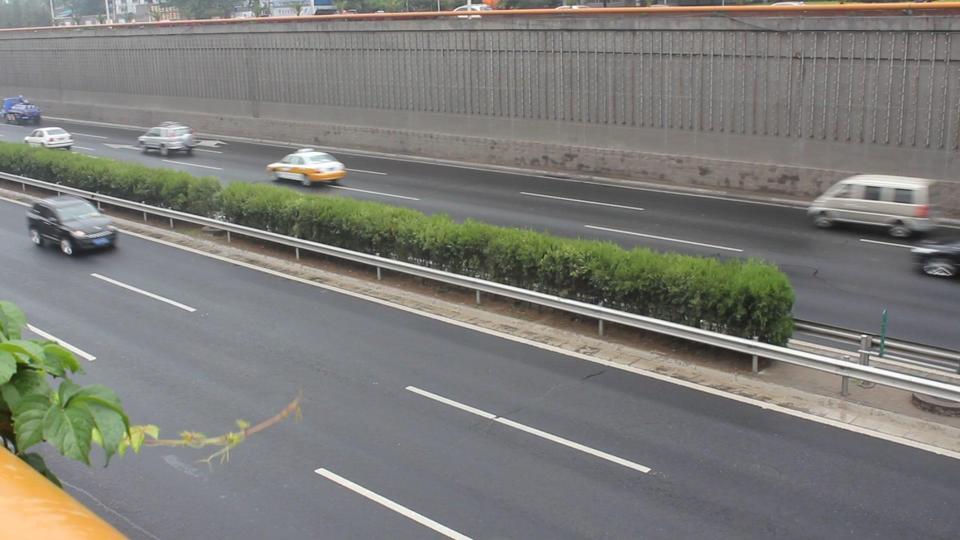

Supplement: Data S1 [file peerj-cs-09-1411-s001.zip › dataset/MVI_63552_img00248.jpg]

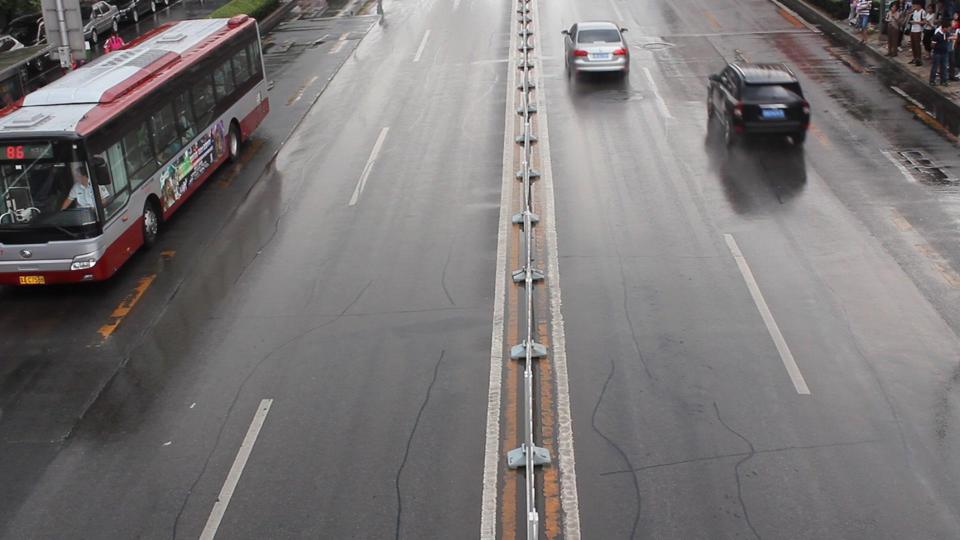

Supplement: Data S1 [file peerj-cs-09-1411-s001.zip › dataset/MVI_63525_img00648.jpg]

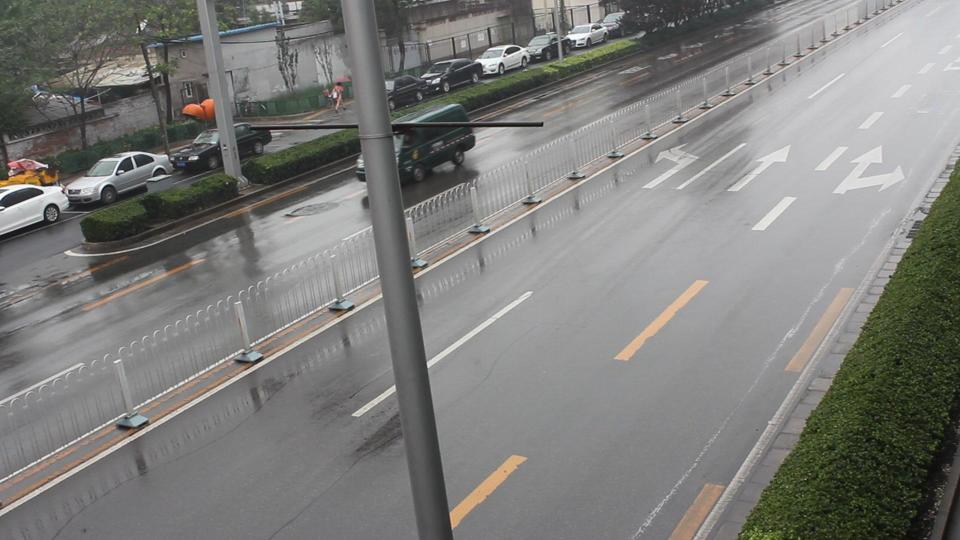

Supplement: Data S1 [file peerj-cs-09-1411-s001.zip › dataset/MVI_63544_img00447.jpg]

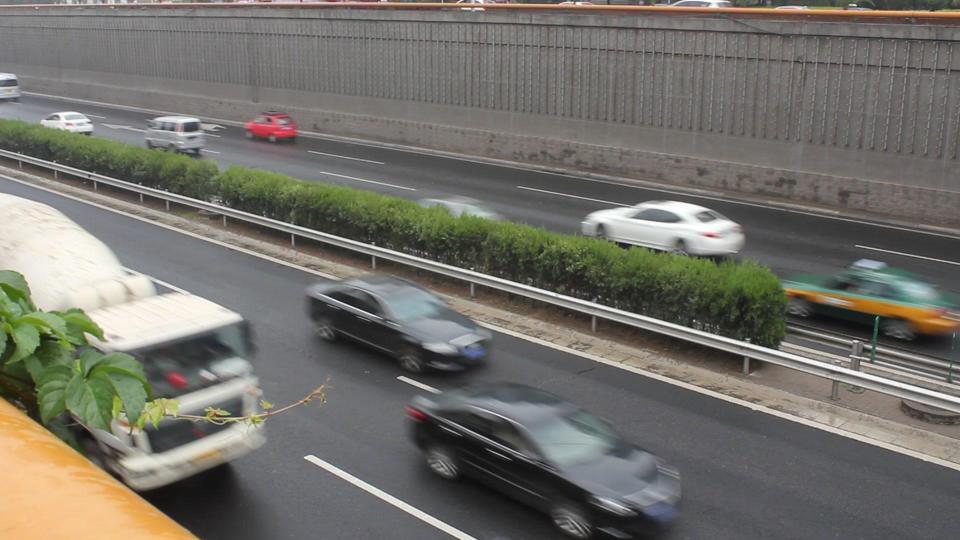

Supplement: Data S1 [file peerj-cs-09-1411-s001.zip › dataset/MVI_63553_img00071.jpg]

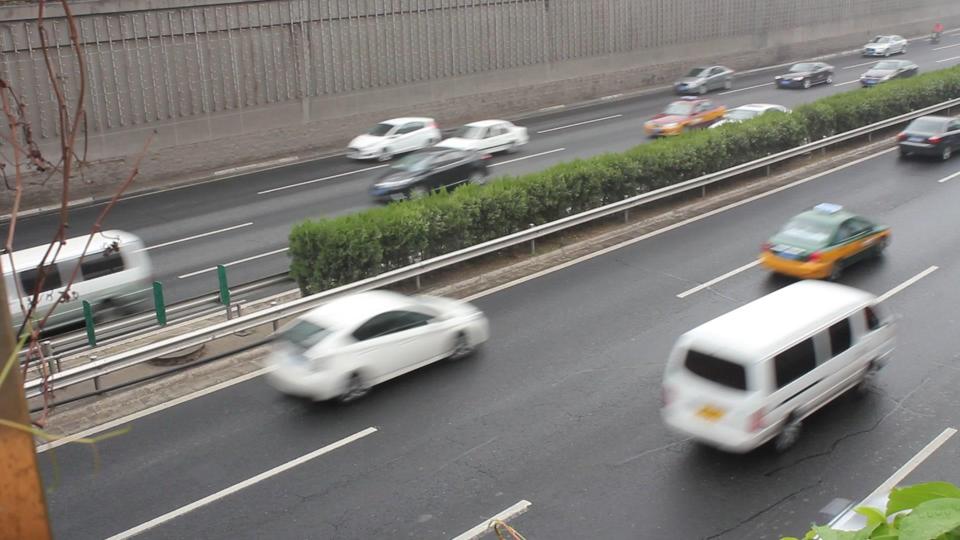

Supplement: Data S1 [file peerj-cs-09-1411-s001.zip › dataset/MVI_63563_img00289.jpg]

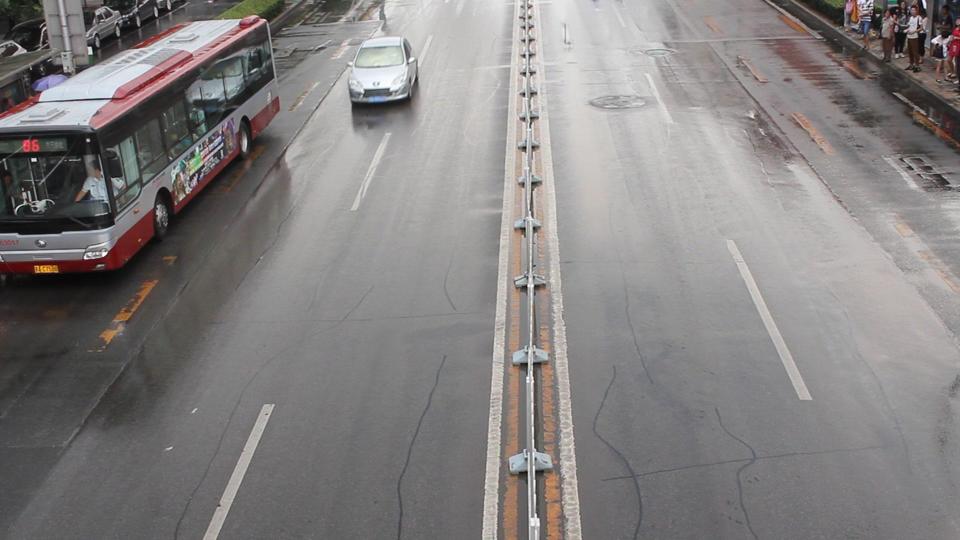

Supplement: Data S1 [file peerj-cs-09-1411-s001.zip › dataset/MVI_63525_img00270.jpg]

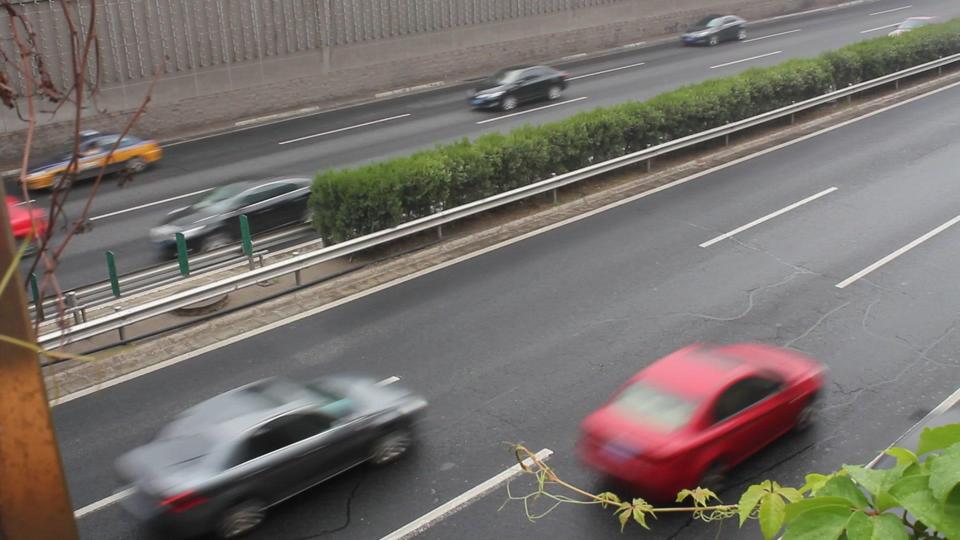

Supplement: Data S1 [file peerj-cs-09-1411-s001.zip › dataset/MVI_63563_img01357.jpg]

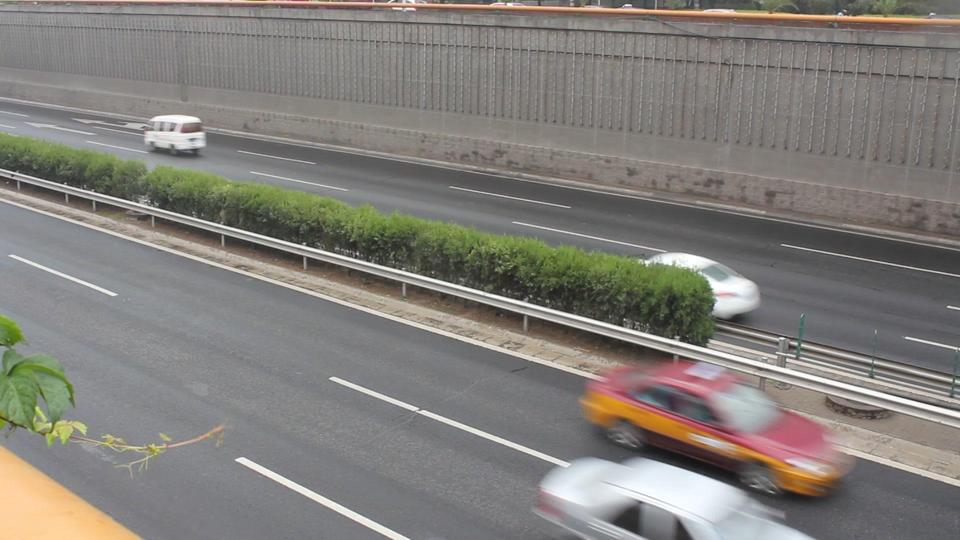

Supplement: Data S1 [file peerj-cs-09-1411-s001.zip › dataset/MVI_63554_img01385.jpg]

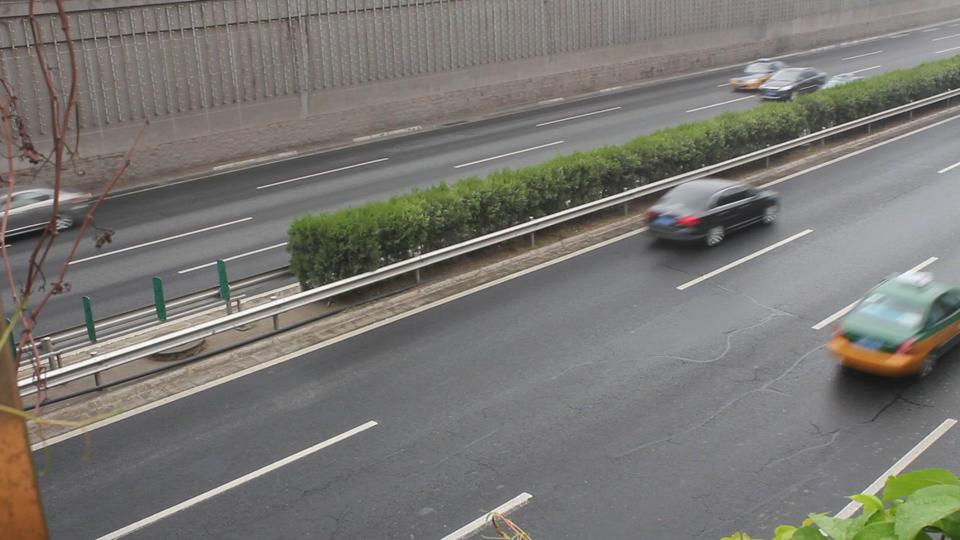

Supplement: Data S1 [file peerj-cs-09-1411-s001.zip › dataset/MVI_63563_img00707.jpg]

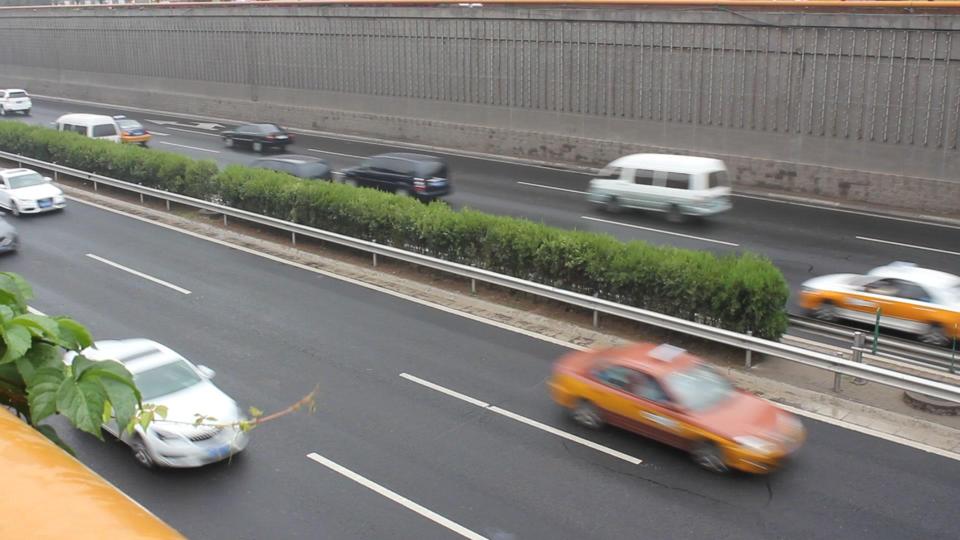

Supplement: Data S1 [file peerj-cs-09-1411-s001.zip › dataset/MVI_63552_img01023.jpg]

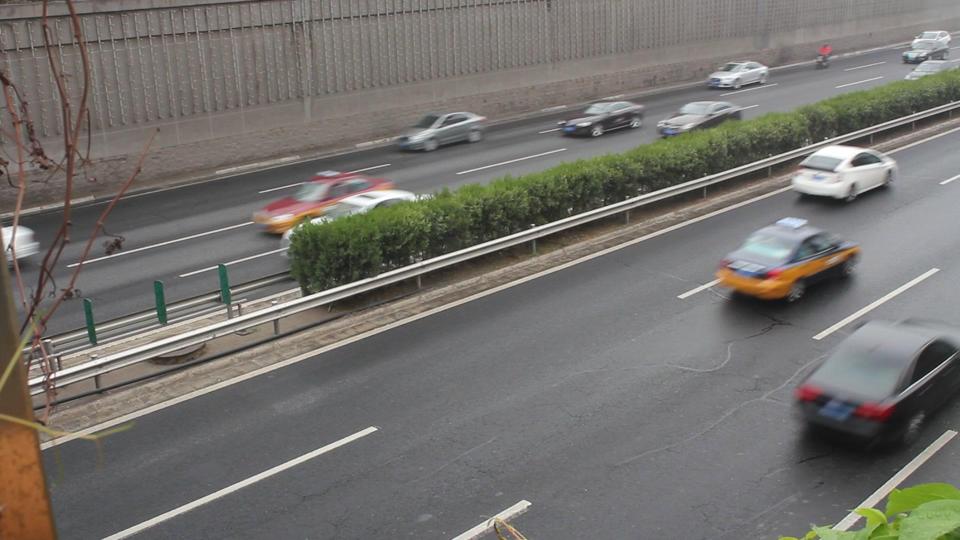

Supplement: Data S1 [file peerj-cs-09-1411-s001.zip › dataset/MVI_63563_img00317.jpg]

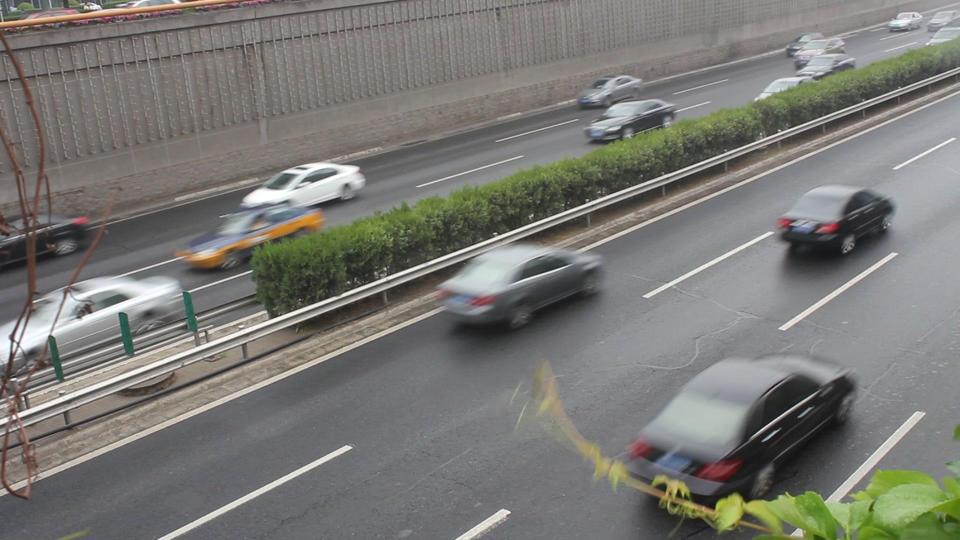

Supplement: Data S1 [file peerj-cs-09-1411-s001.zip › dataset/MVI_63561_img00536.jpg]

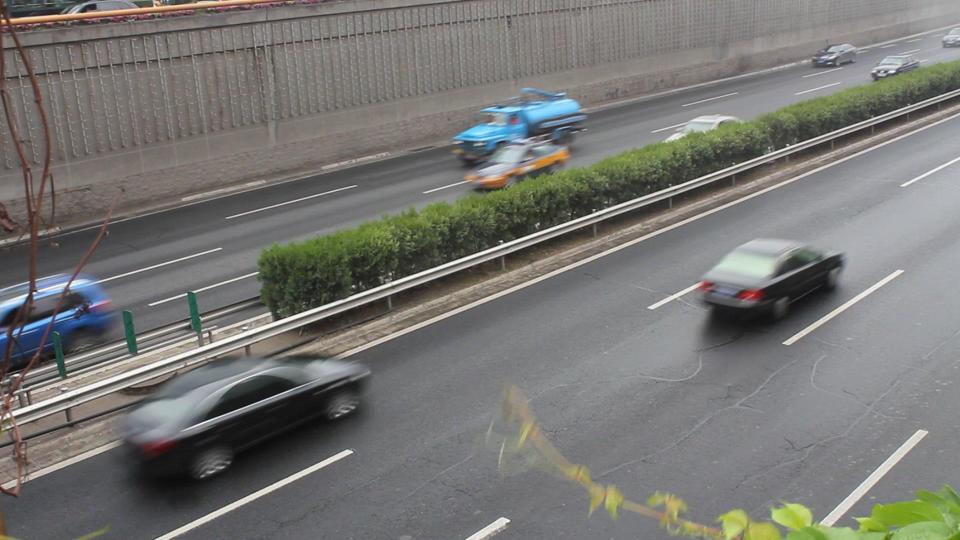

Supplement: Data S1 [file peerj-cs-09-1411-s001.zip › dataset/MVI_63561_img00720.jpg]

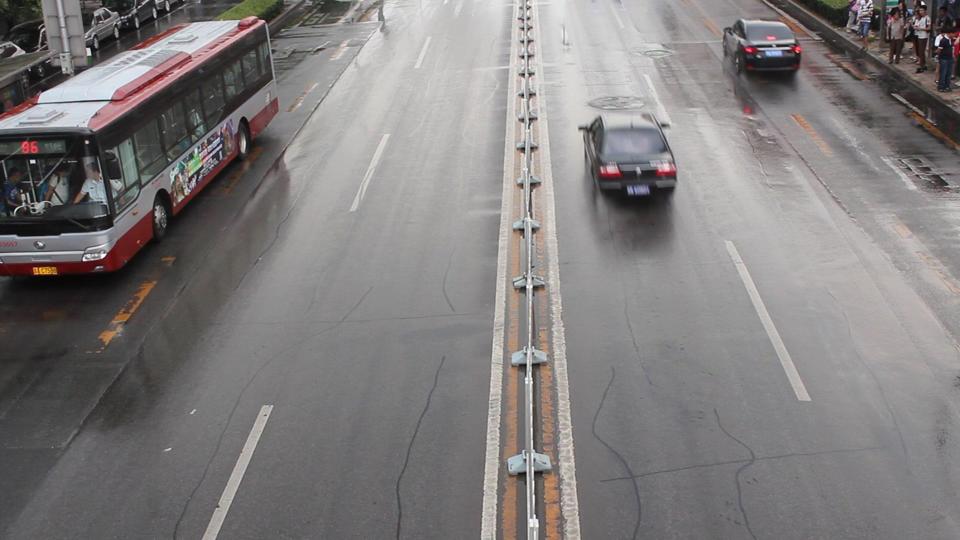

Supplement: Data S1 [file peerj-cs-09-1411-s001.zip › dataset/MVI_63525_img00452.jpg]

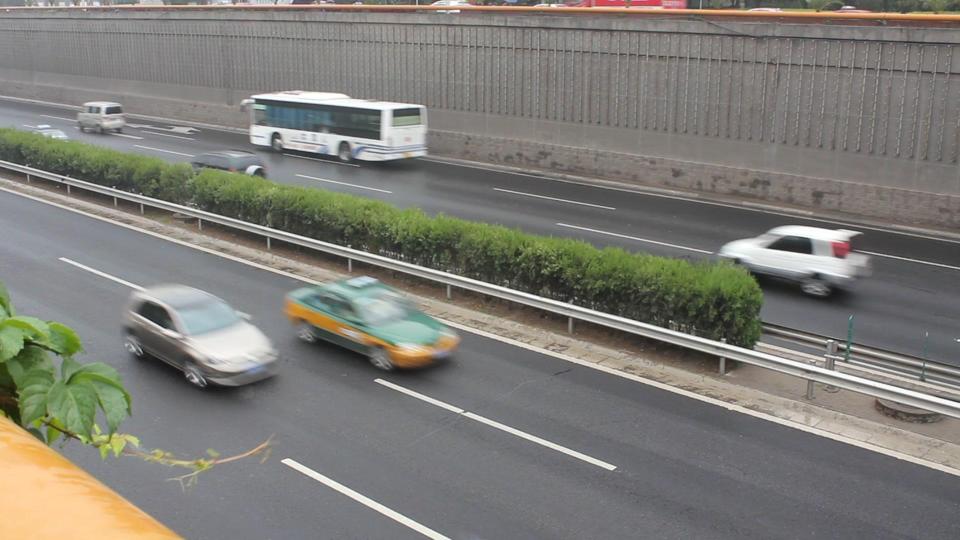

Supplement: Data S1 [file peerj-cs-09-1411-s001.zip › dataset/MVI_63553_img00486.jpg]

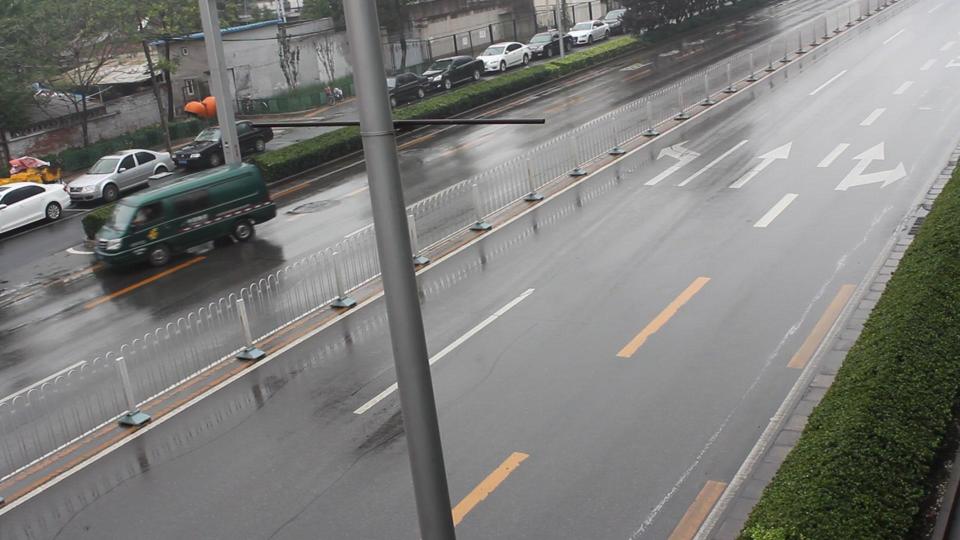

Supplement: Data S1 [file peerj-cs-09-1411-s001.zip › dataset/MVI_63544_img00503.jpg]

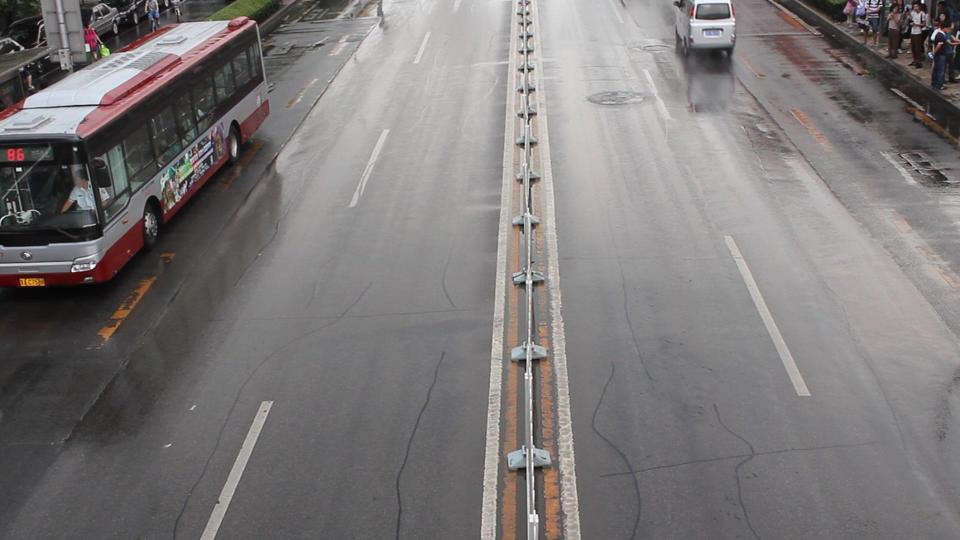

Supplement: Data S1 [file peerj-cs-09-1411-s001.zip › dataset/MVI_63525_img00718.jpg]

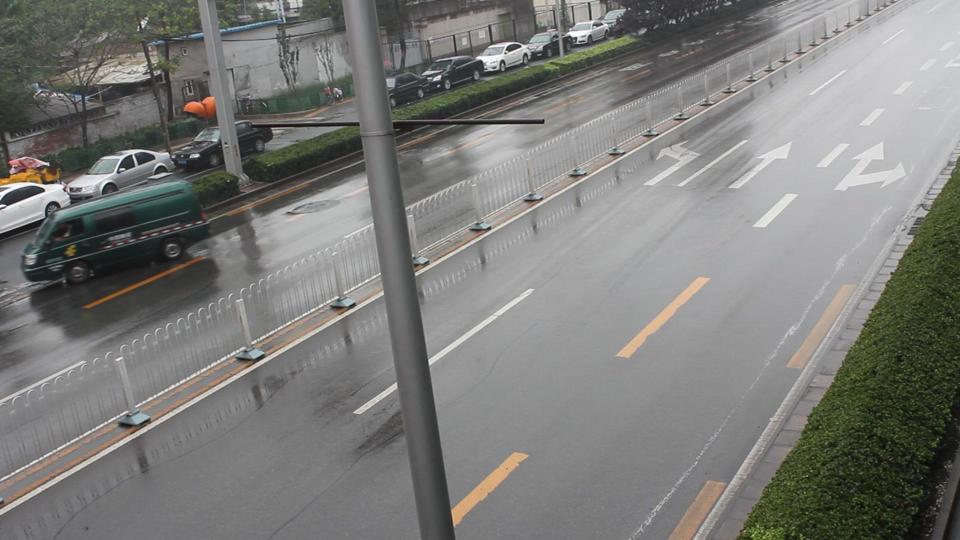

Supplement: Data S1 [file peerj-cs-09-1411-s001.zip › dataset/MVI_63544_img00517.jpg]

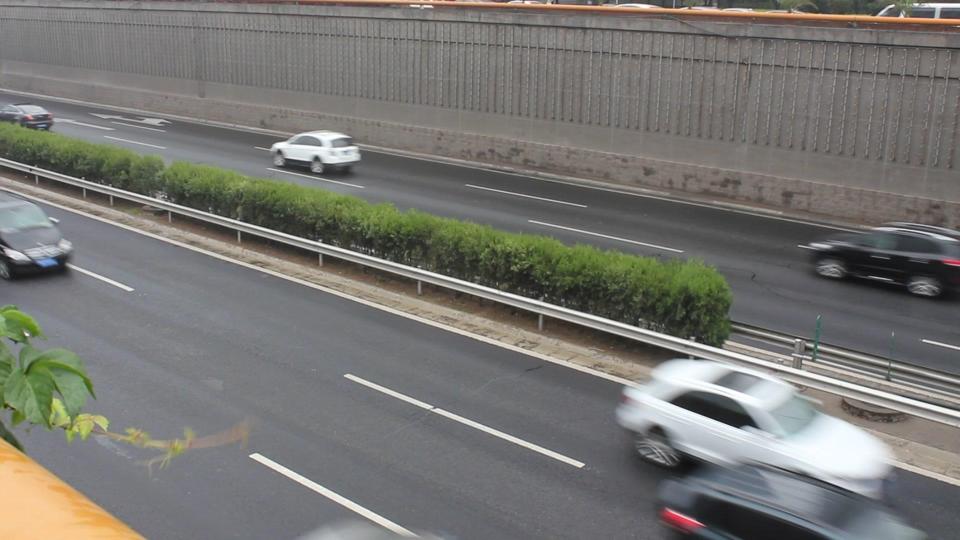

Supplement: Data S1 [file peerj-cs-09-1411-s001.zip › dataset/MVI_63553_img01149.jpg]

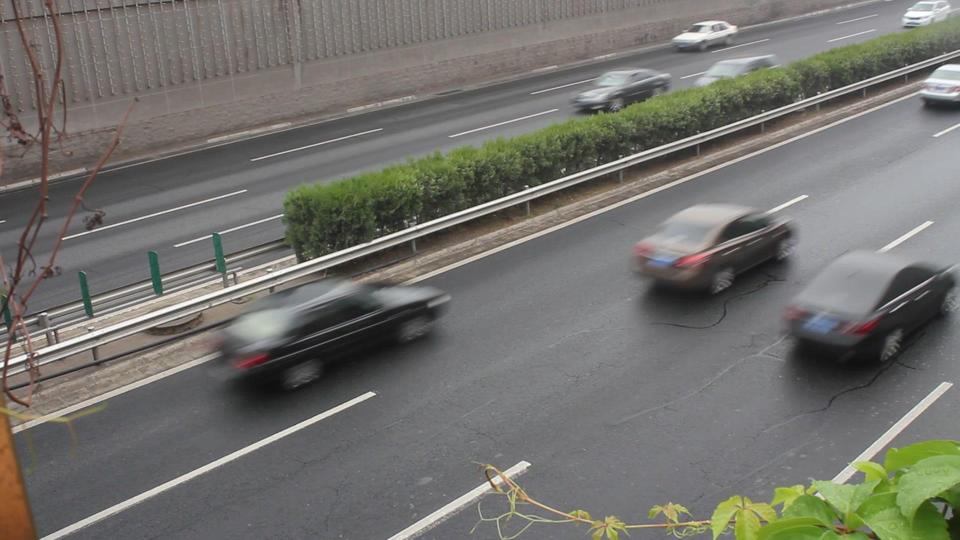

Supplement: Data S1 [file peerj-cs-09-1411-s001.zip › dataset/MVI_63562_img00447.jpg]

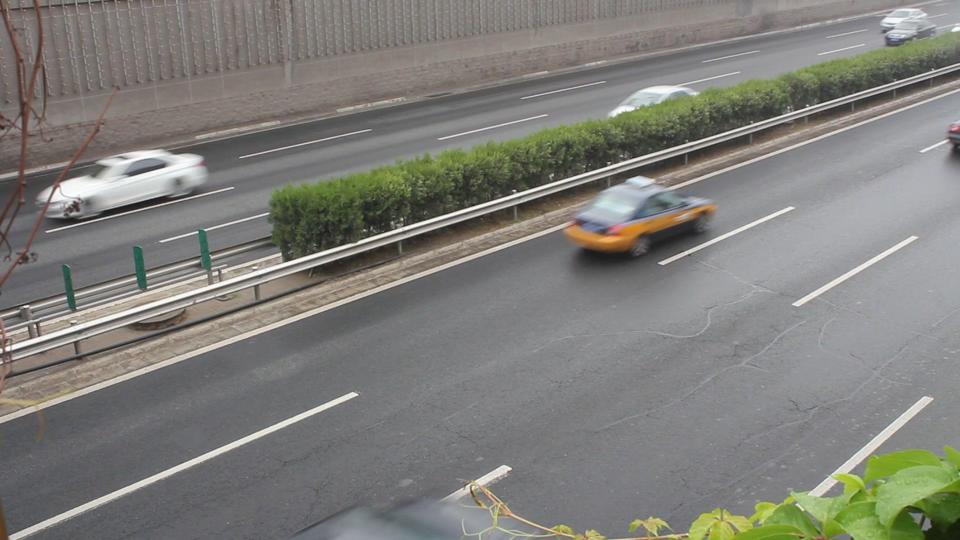

Supplement: Data S1 [file peerj-cs-09-1411-s001.zip › dataset/MVI_63562_img00644.jpg]

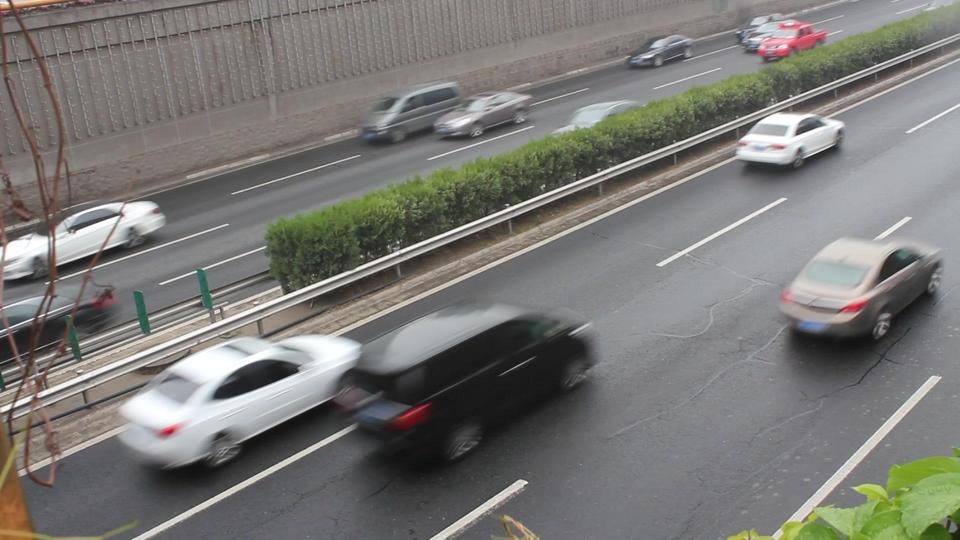

Supplement: Data S1 [file peerj-cs-09-1411-s001.zip › dataset/MVI_63561_img00314.jpg]

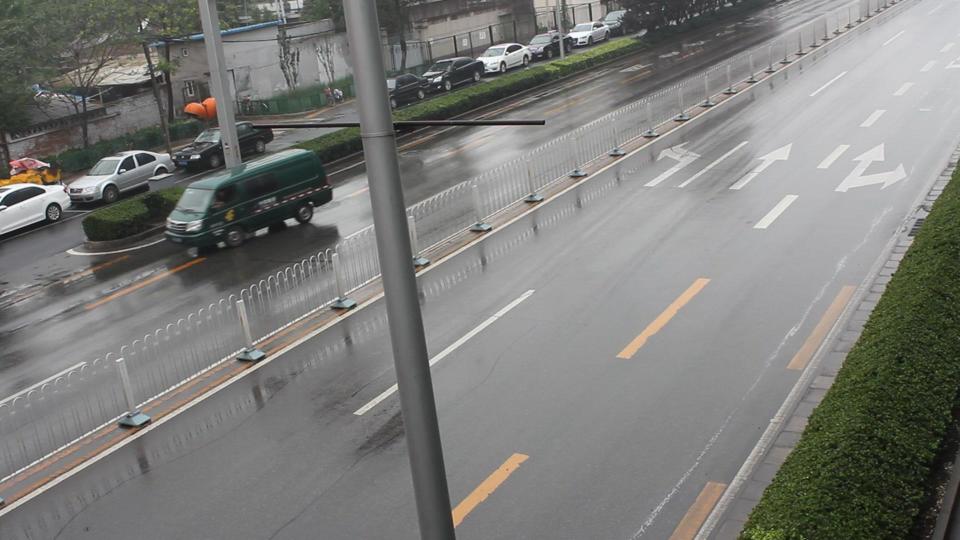

Supplement: Data S1 [file peerj-cs-09-1411-s001.zip › dataset/MVI_63544_img00489.jpg]

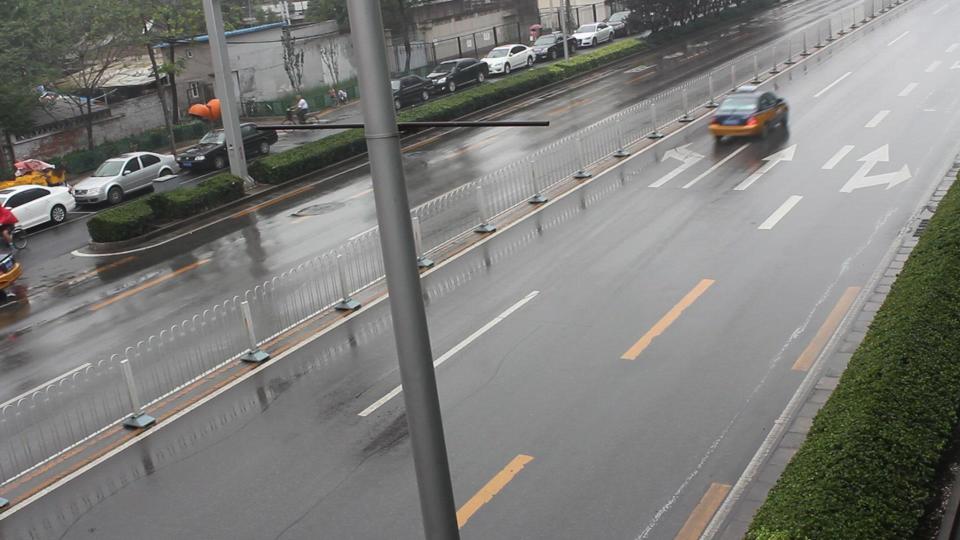

Supplement: Data S1 [file peerj-cs-09-1411-s001.zip › dataset/MVI_63544_img00853.jpg]

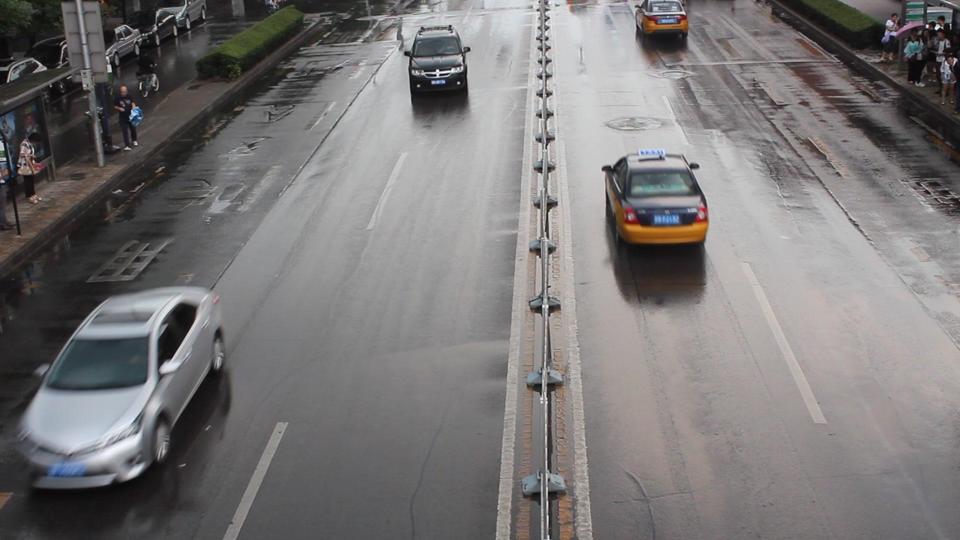

Supplement: Data S1 [file peerj-cs-09-1411-s001.zip › dataset/MVI_63521_img01989.jpg]

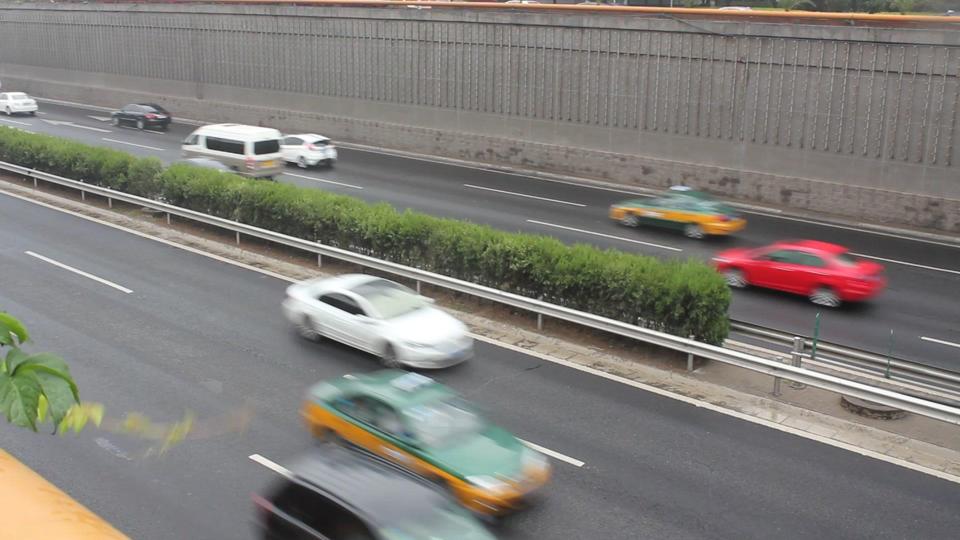

Supplement: Data S1 [file peerj-cs-09-1411-s001.zip › dataset/MVI_63554_img00446.jpg]

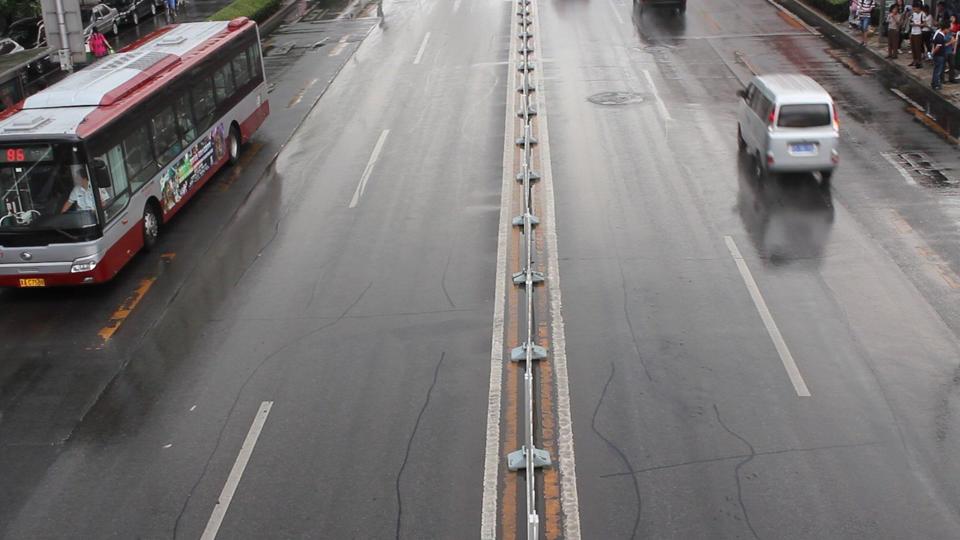

Supplement: Data S1 [file peerj-cs-09-1411-s001.zip › dataset/MVI_63525_img00690.jpg]

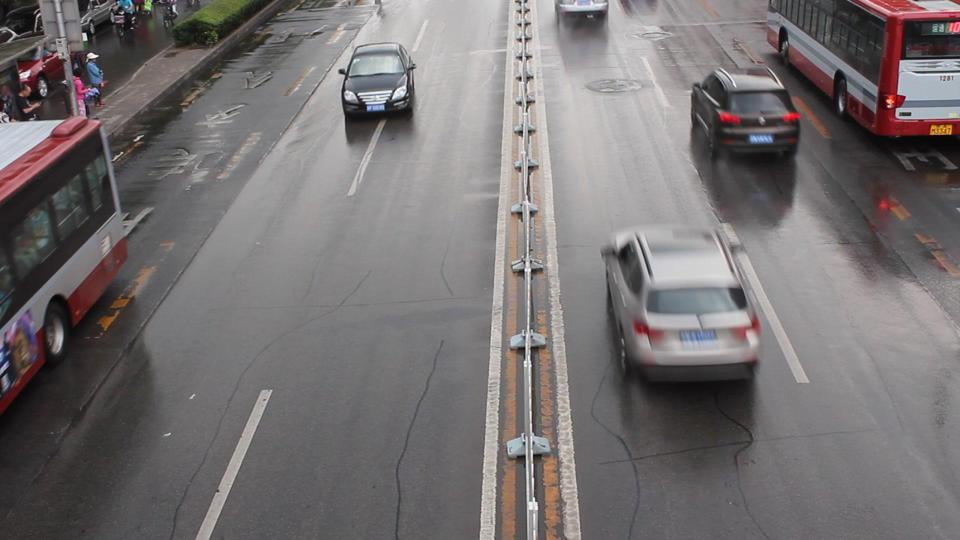

Supplement: Data S1 [file peerj-cs-09-1411-s001.zip › dataset/MVI_63525_img00914.jpg]

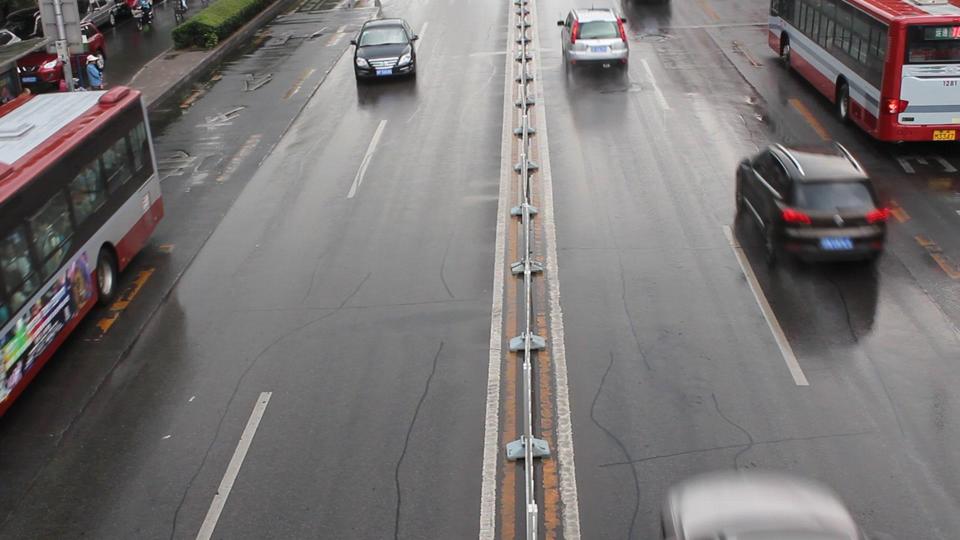

Supplement: Data S1 [file peerj-cs-09-1411-s001.zip › dataset/MVI_63525_img00900.jpg]

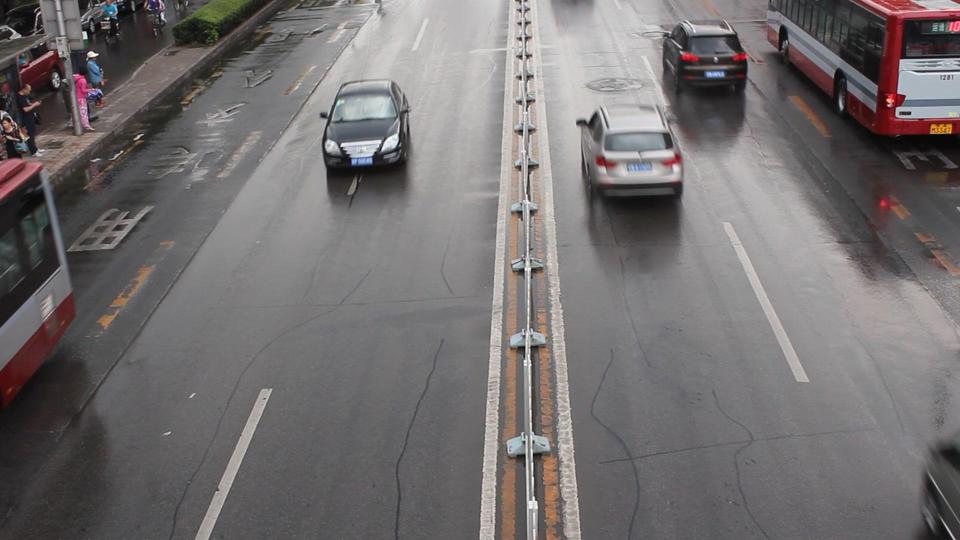

Supplement: Data S1 [file peerj-cs-09-1411-s001.zip › dataset/MVI_63525_img00928.jpg]

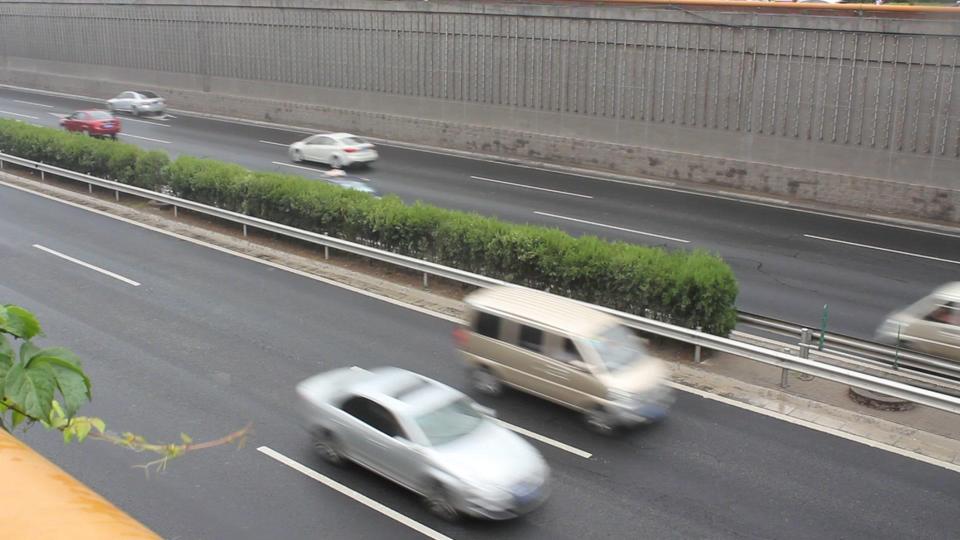

Supplement: Data S1 [file peerj-cs-09-1411-s001.zip › dataset/MVI_63554_img00042.jpg]

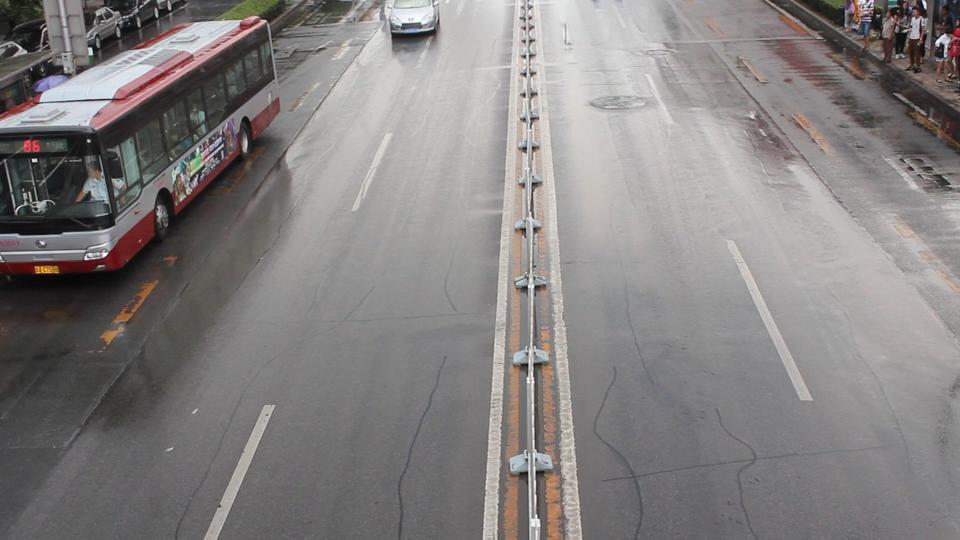

Supplement: Data S1 [file peerj-cs-09-1411-s001.zip › dataset/MVI_63525_img00242.jpg]

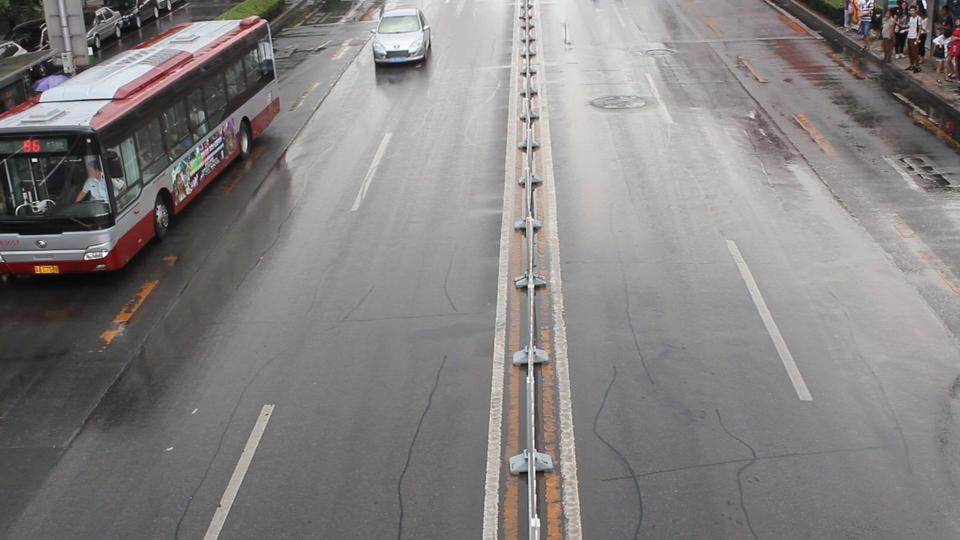

Supplement: Data S1 [file peerj-cs-09-1411-s001.zip › dataset/MVI_63525_img00256.jpg]

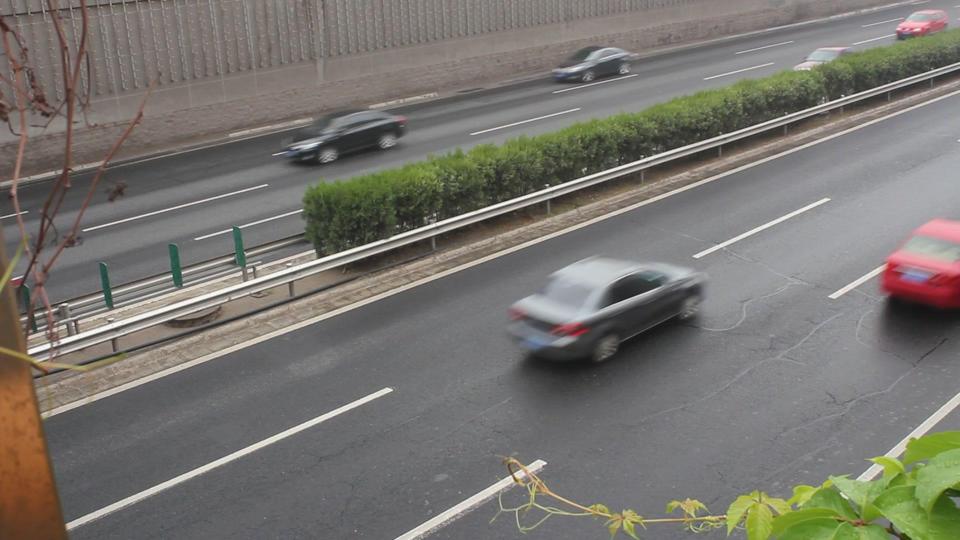

Supplement: Data S1 [file peerj-cs-09-1411-s001.zip › dataset/MVI_63563_img01370.jpg]

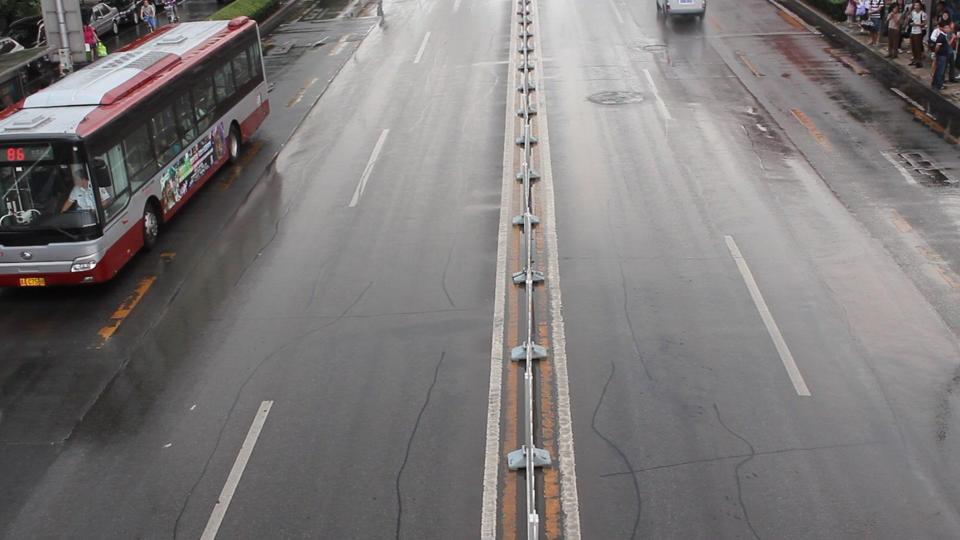

Supplement: Data S1 [file peerj-cs-09-1411-s001.zip › dataset/MVI_63525_img00732.jpg]

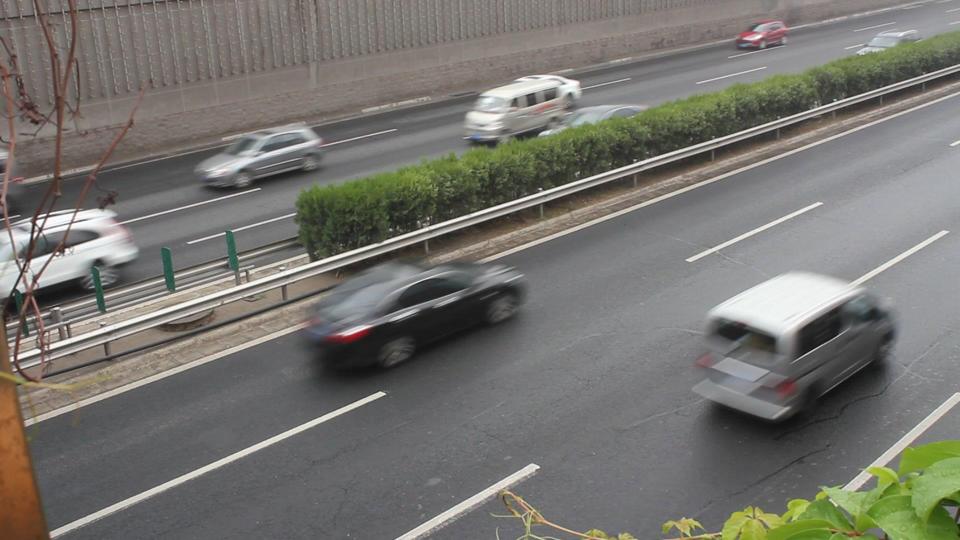

Supplement: Data S1 [file peerj-cs-09-1411-s001.zip › dataset/MVI_63563_img01199.jpg]

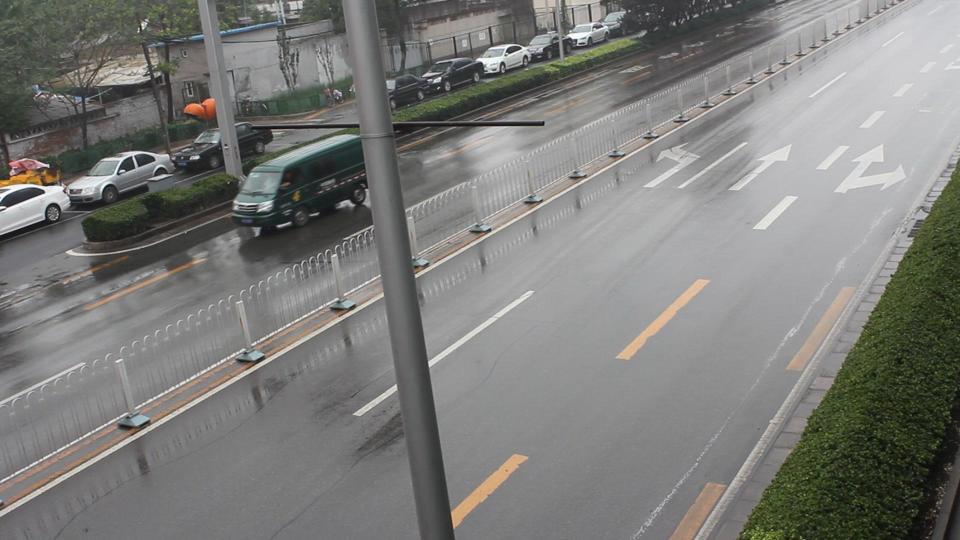

Supplement: Data S1 [file peerj-cs-09-1411-s001.zip › dataset/MVI_63544_img00475.jpg]

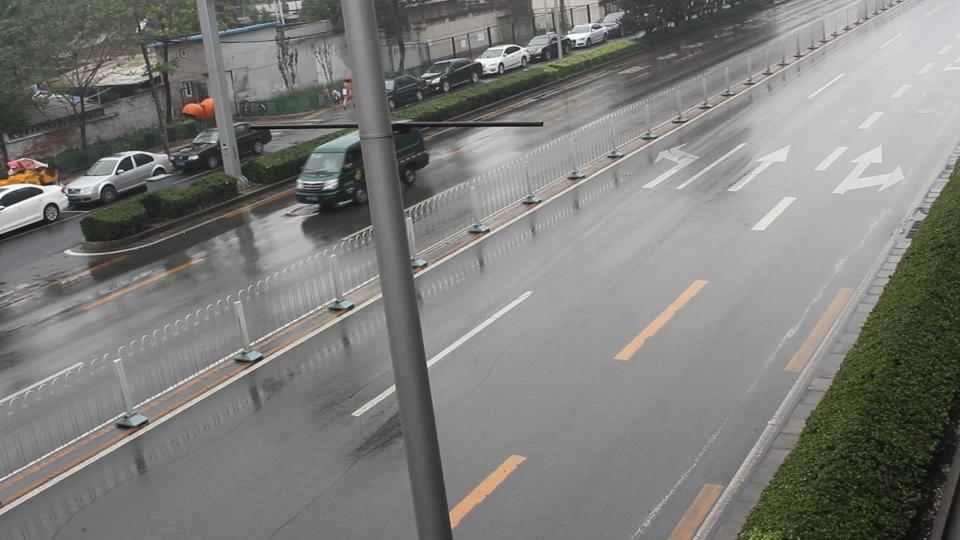

Supplement: Data S1 [file peerj-cs-09-1411-s001.zip › dataset/MVI_63544_img00461.jpg]

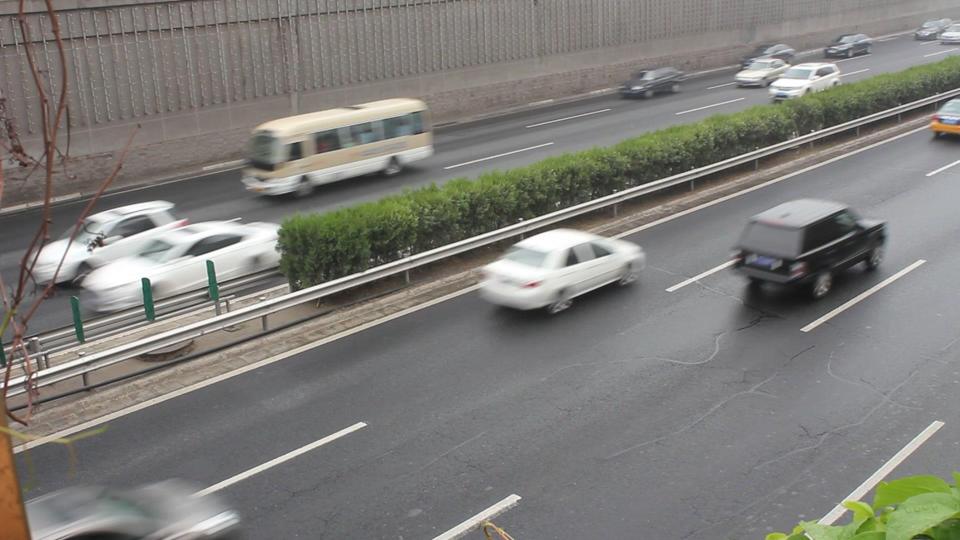

Supplement: Data S1 [file peerj-cs-09-1411-s001.zip › dataset/MVI_63562_img01149.jpg]

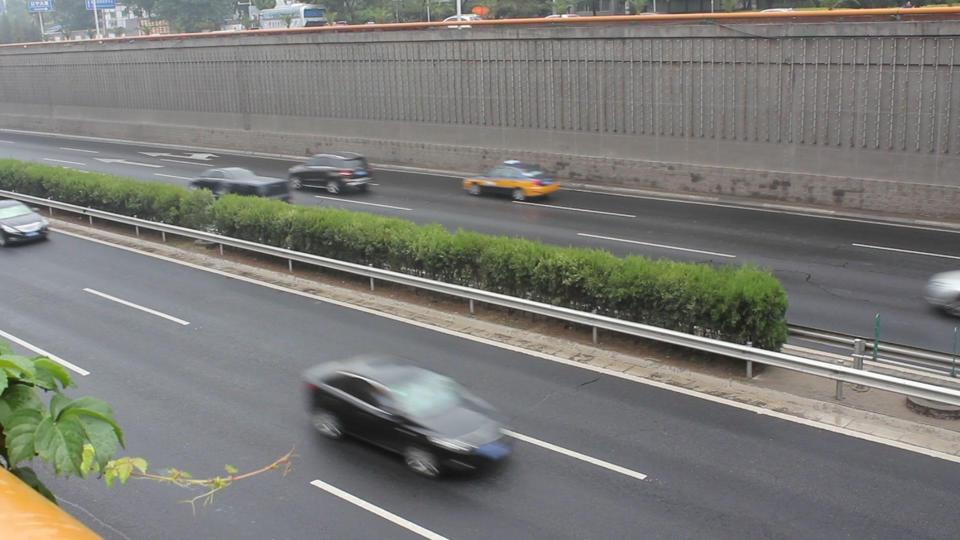

Supplement: Data S1 [file peerj-cs-09-1411-s001.zip › dataset/MVI_63552_img00087.jpg]

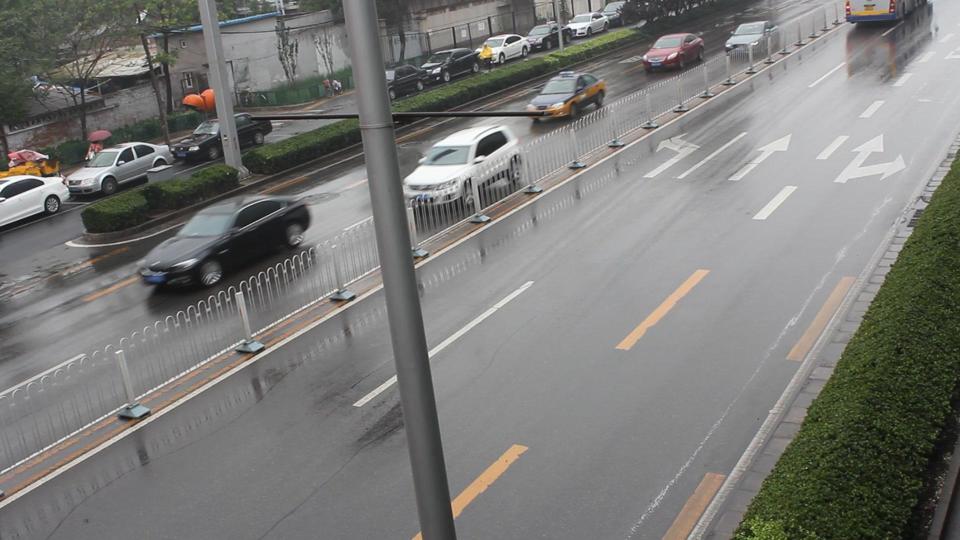

Supplement: Data S1 [file peerj-cs-09-1411-s001.zip › dataset/MVI_63544_img00139.jpg]

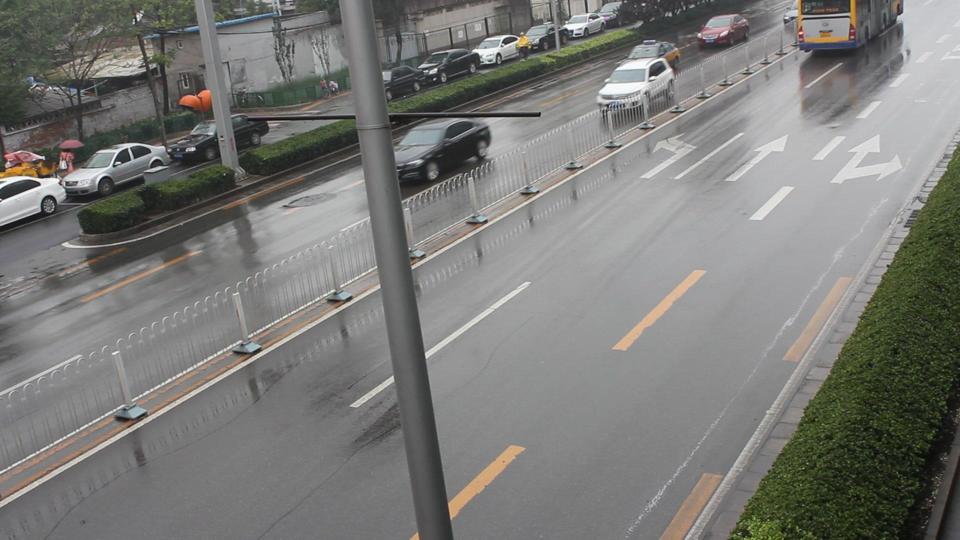

Supplement: Data S1 [file peerj-cs-09-1411-s001.zip › dataset/MVI_63544_img00111.jpg]

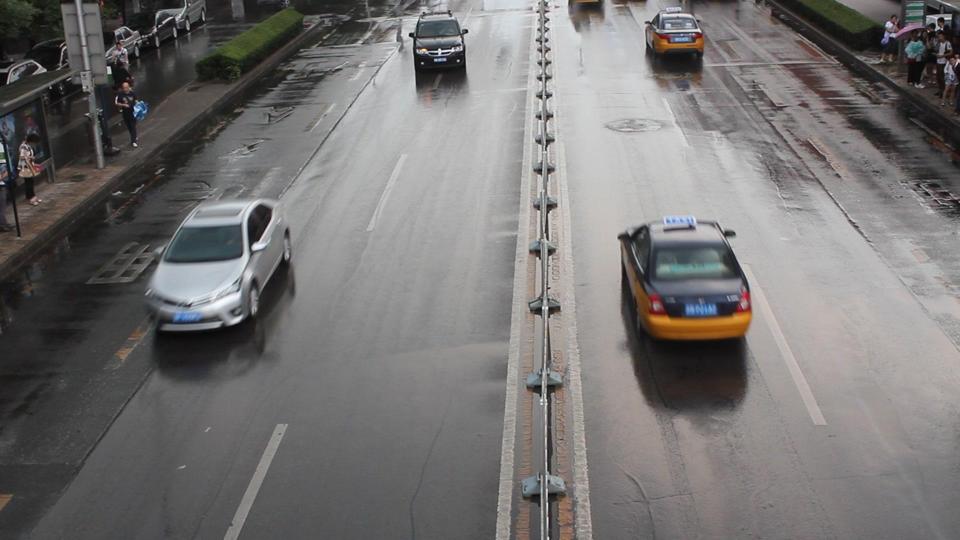

Supplement: Data S1 [file peerj-cs-09-1411-s001.zip › dataset/MVI_63521_img01975.jpg]

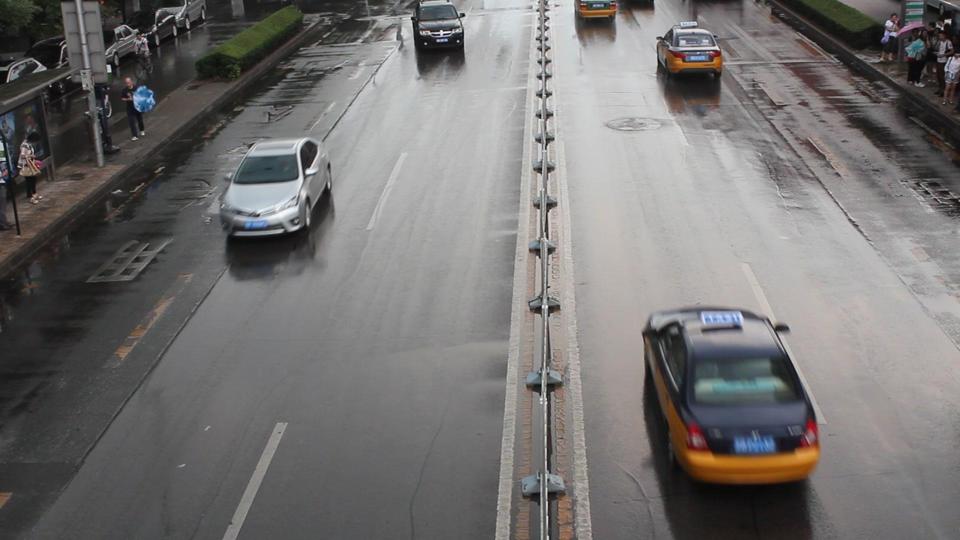

Supplement: Data S1 [file peerj-cs-09-1411-s001.zip › dataset/MVI_63521_img01961.jpg]

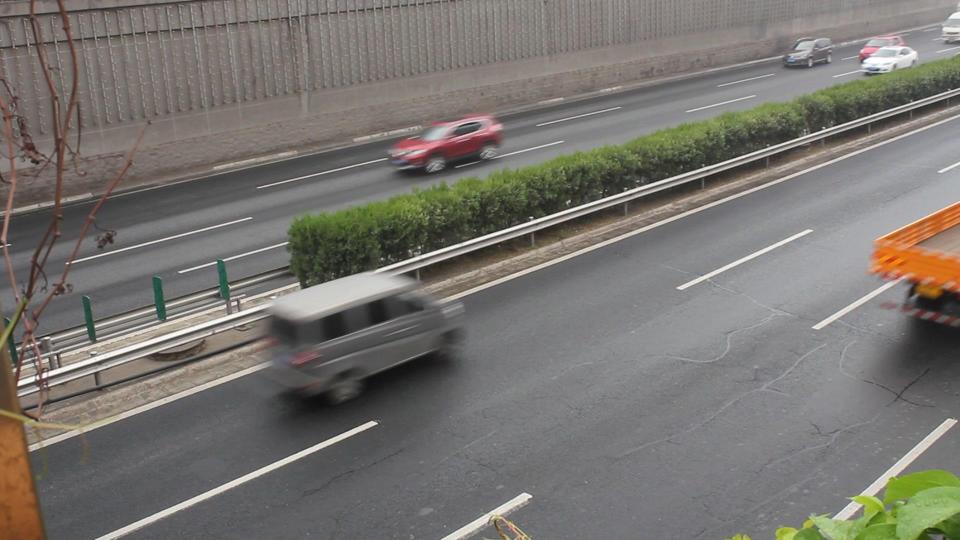

Supplement: Data S1 [file peerj-cs-09-1411-s001.zip › dataset/MVI_63563_img00863.jpg]

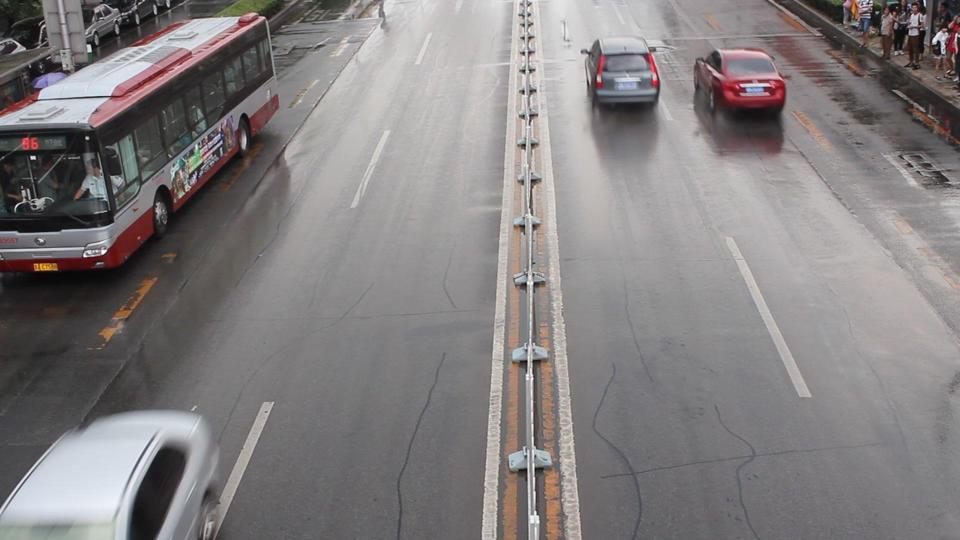

Supplement: Data S1 [file peerj-cs-09-1411-s001.zip › dataset/MVI_63525_img00326.jpg]

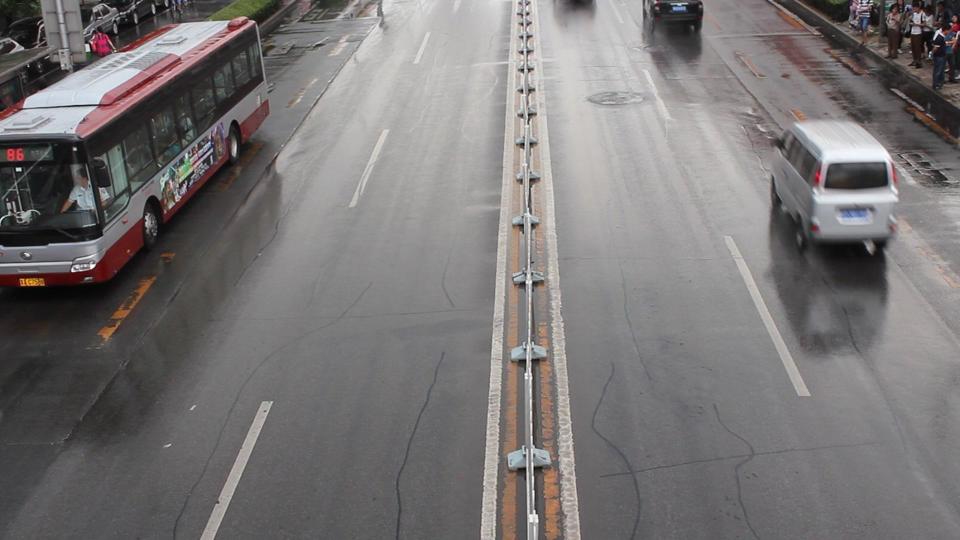

Supplement: Data S1 [file peerj-cs-09-1411-s001.zip › dataset/MVI_63525_img00681.jpg]

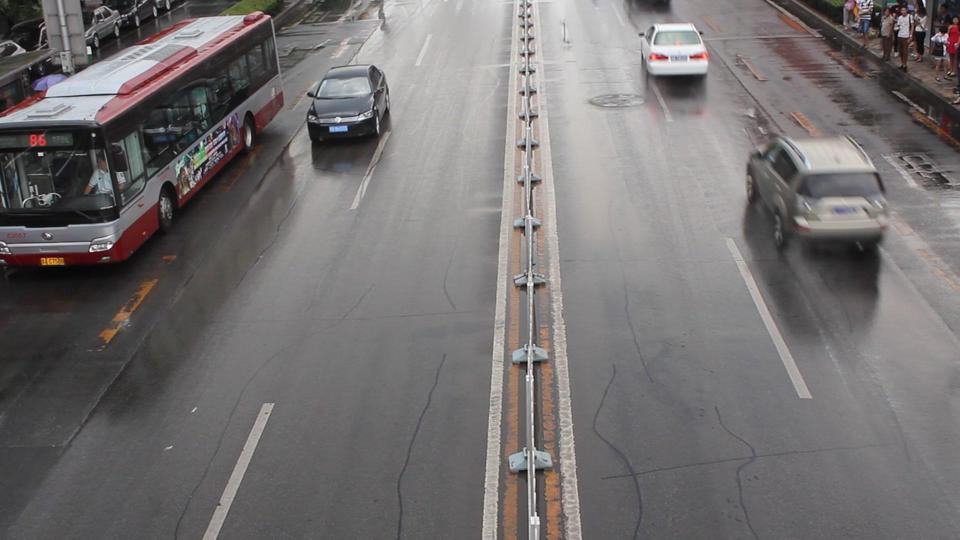

Supplement: Data S1 [file peerj-cs-09-1411-s001.zip › dataset/MVI_63525_img00130.jpg]

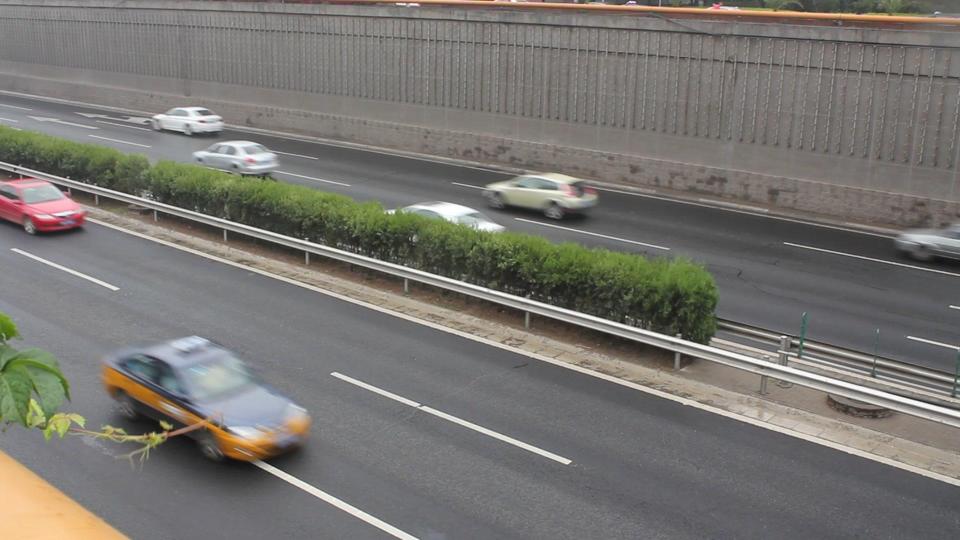

Supplement: Data S1 [file peerj-cs-09-1411-s001.zip › dataset/MVI_63554_img01173.jpg]

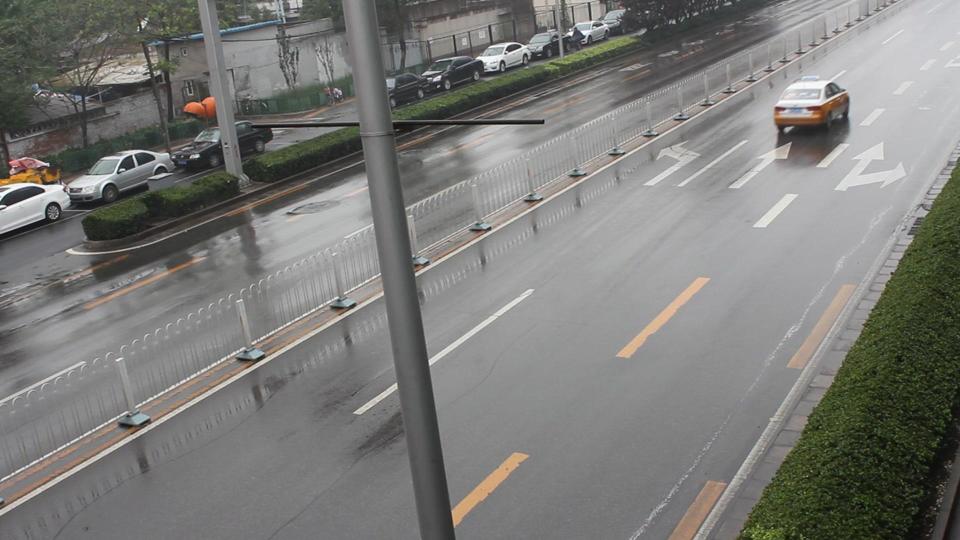

Supplement: Data S1 [file peerj-cs-09-1411-s001.zip › dataset/MVI_63544_img01155.jpg]

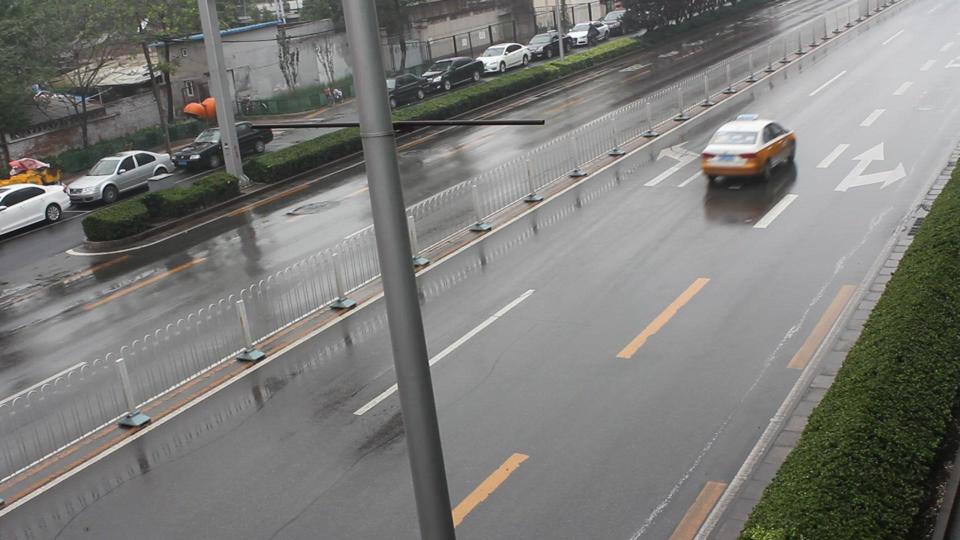

Supplement: Data S1 [file peerj-cs-09-1411-s001.zip › dataset/MVI_63544_img01141.jpg]

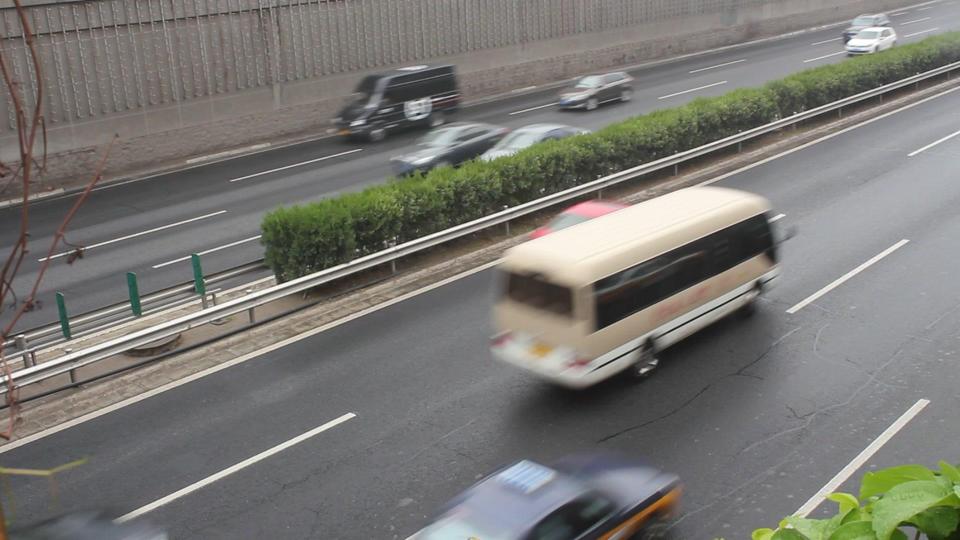

Supplement: Data S1 [file peerj-cs-09-1411-s001.zip › dataset/MVI_63561_img01227.jpg]

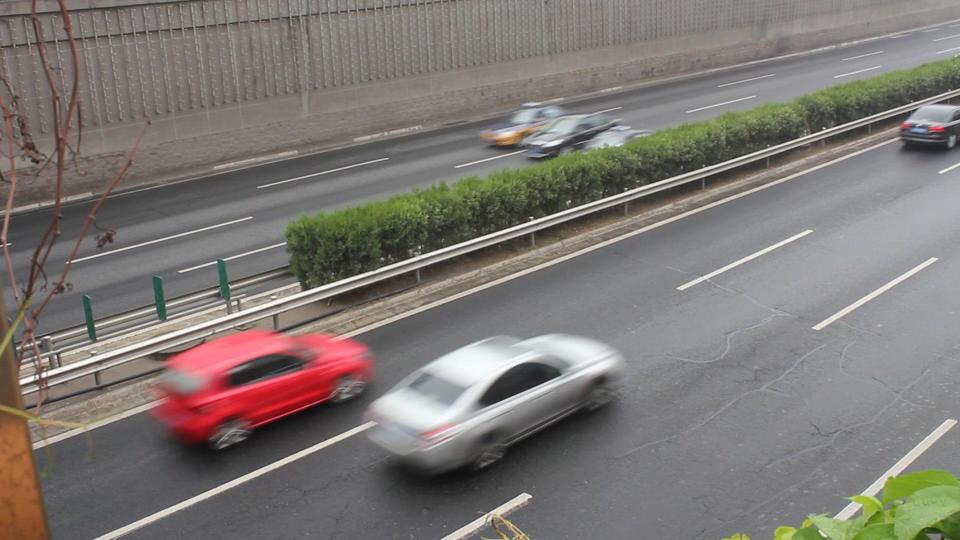

Supplement: Data S1 [file peerj-cs-09-1411-s001.zip › dataset/MVI_63563_img00730.jpg]

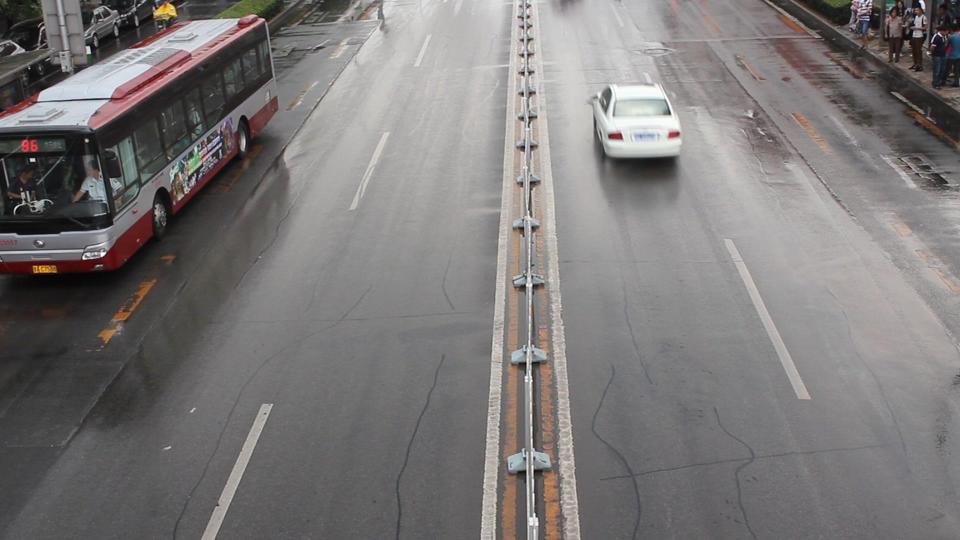

Supplement: Data S1 [file peerj-cs-09-1411-s001.zip › dataset/MVI_63525_img00508.jpg]

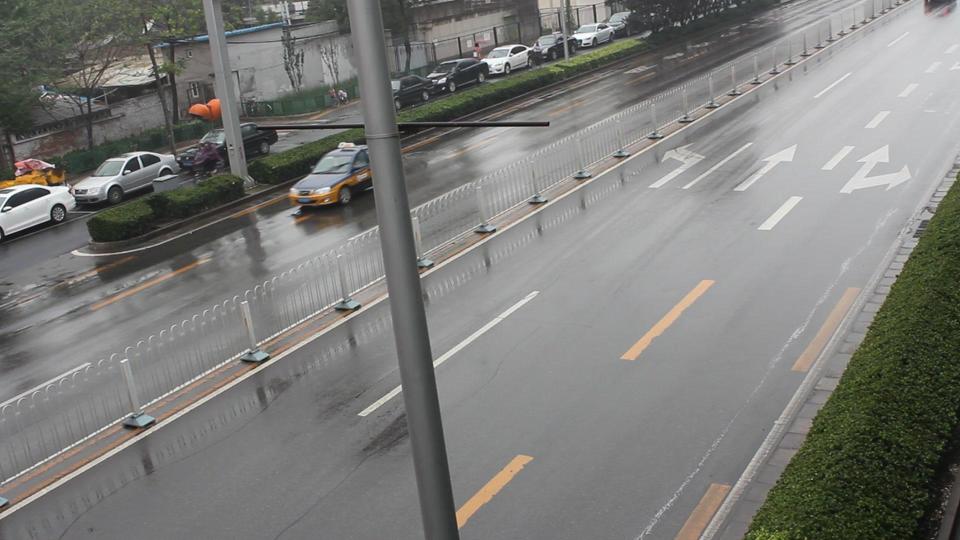

Supplement: Data S1 [file peerj-cs-09-1411-s001.zip › dataset/MVI_63544_img00713.jpg]

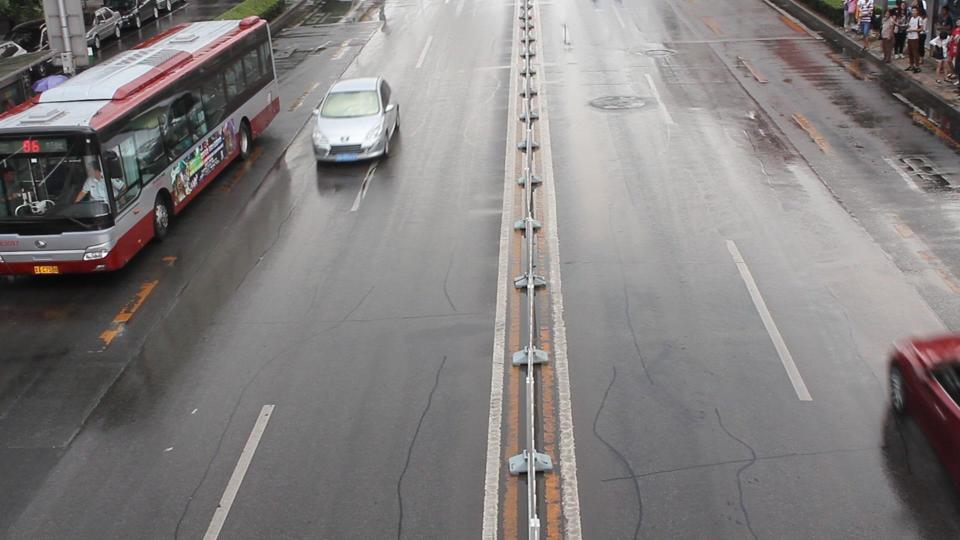

Supplement: Data S1 [file peerj-cs-09-1411-s001.zip › dataset/MVI_63525_img00284.jpg]

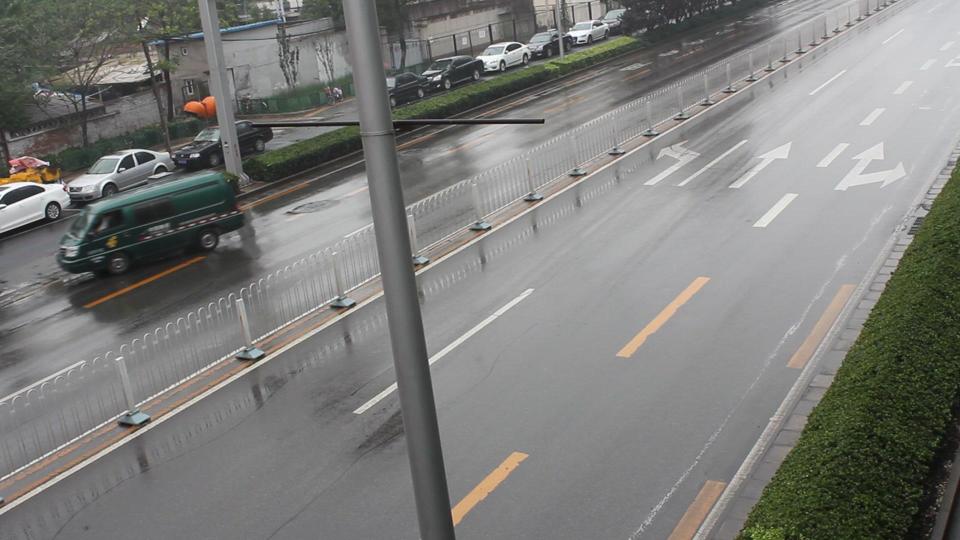

Supplement: Data S1 [file peerj-cs-09-1411-s001.zip › dataset/MVI_63544_img00510.jpg]

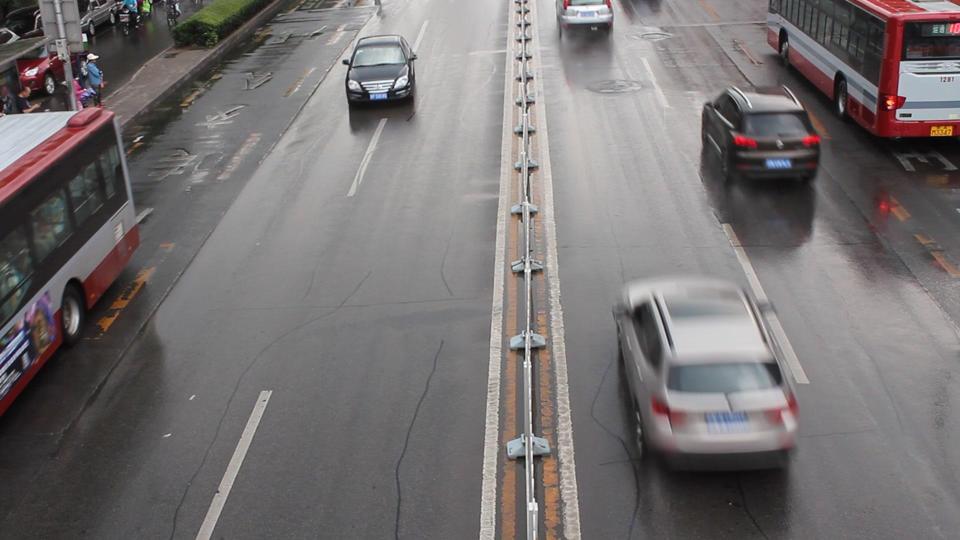

Supplement: Data S1 [file peerj-cs-09-1411-s001.zip › dataset/MVI_63525_img00910.jpg]

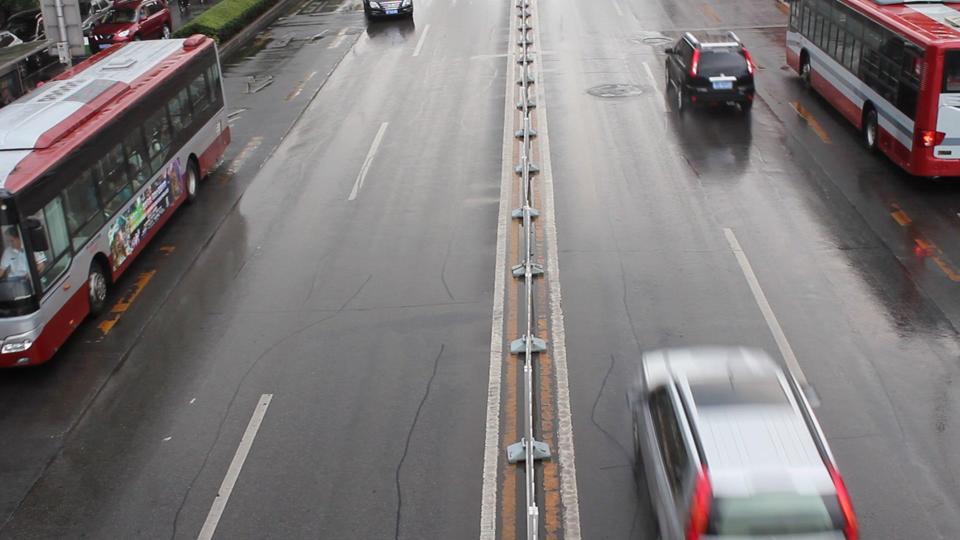

Supplement: Data S1 [file peerj-cs-09-1411-s001.zip › dataset/MVI_63525_img00858.jpg]

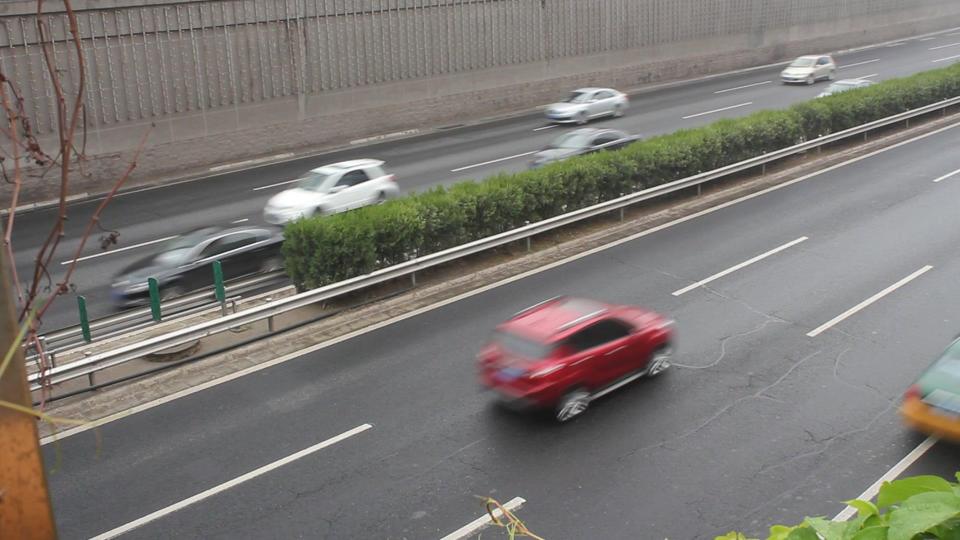

Supplement: Data S1 [file peerj-cs-09-1411-s001.zip › dataset/MVI_63563_img00484.jpg]

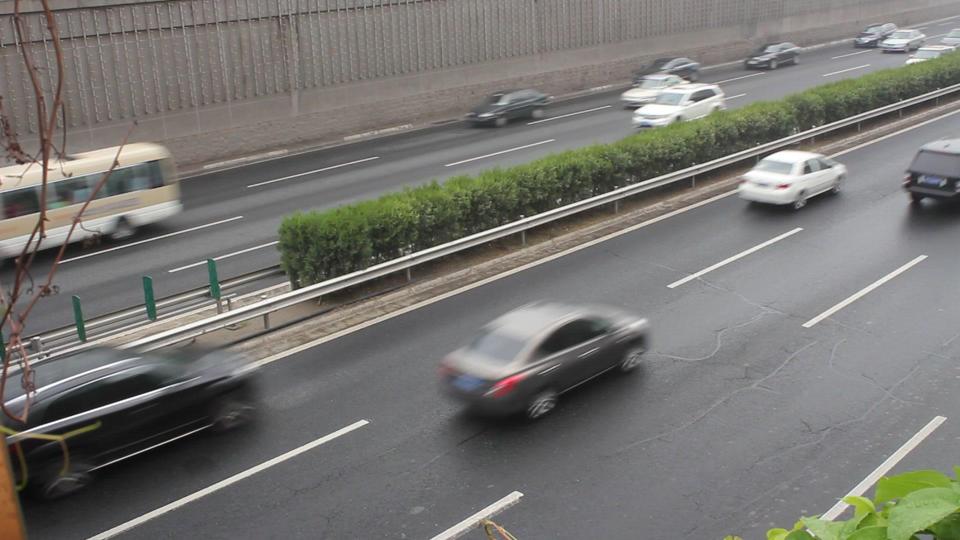

Supplement: Data S1 [file peerj-cs-09-1411-s001.zip › dataset/MVI_63562_img01164.jpg]

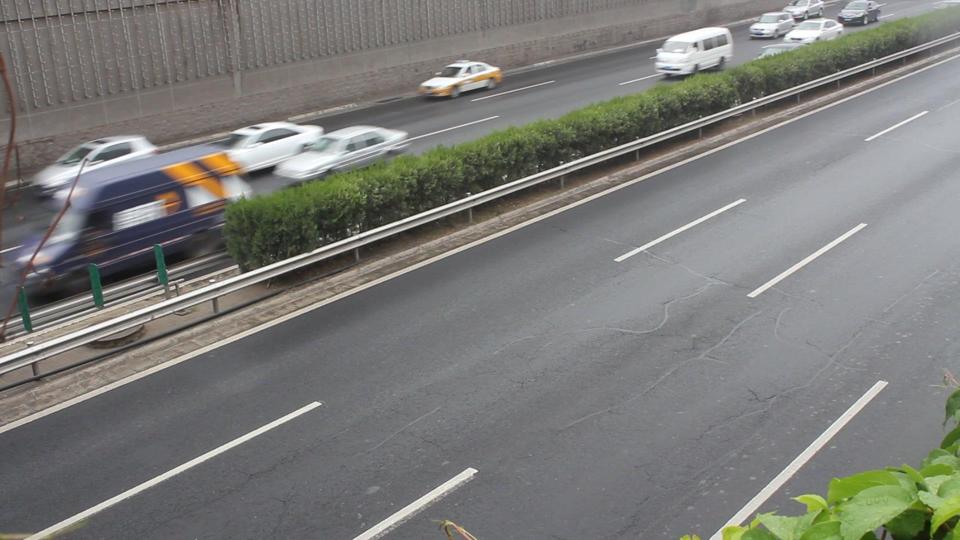

Supplement: Data S1 [file peerj-cs-09-1411-s001.zip › dataset/MVI_63561_img00070.jpg]

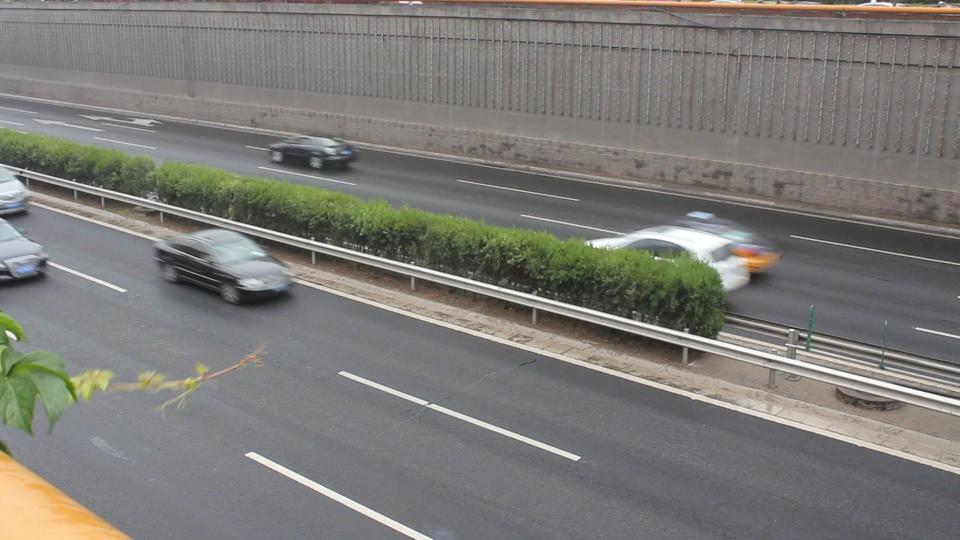

Supplement: Data S1 [file peerj-cs-09-1411-s001.zip › dataset/MVI_63553_img01006.jpg]

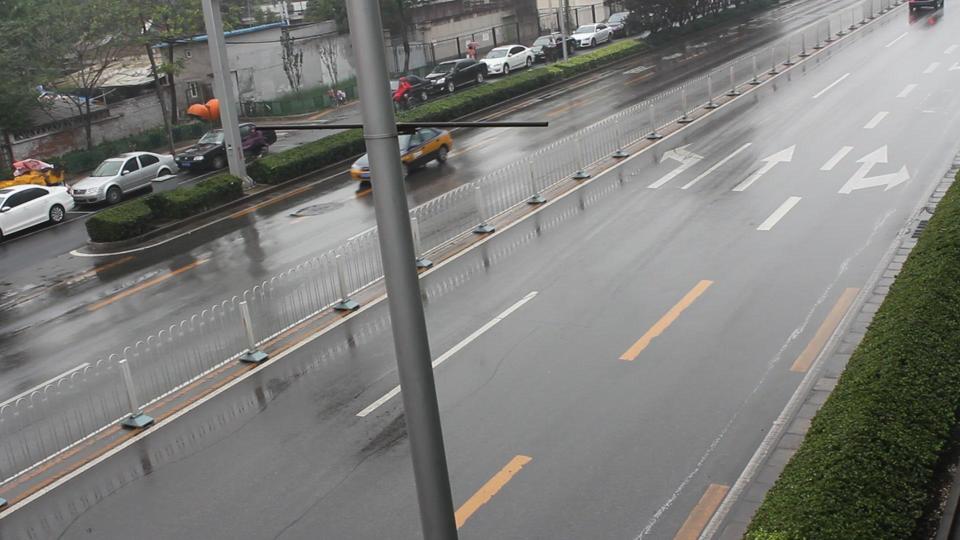

Supplement: Data S1 [file peerj-cs-09-1411-s001.zip › dataset/MVI_63544_img00699.jpg]

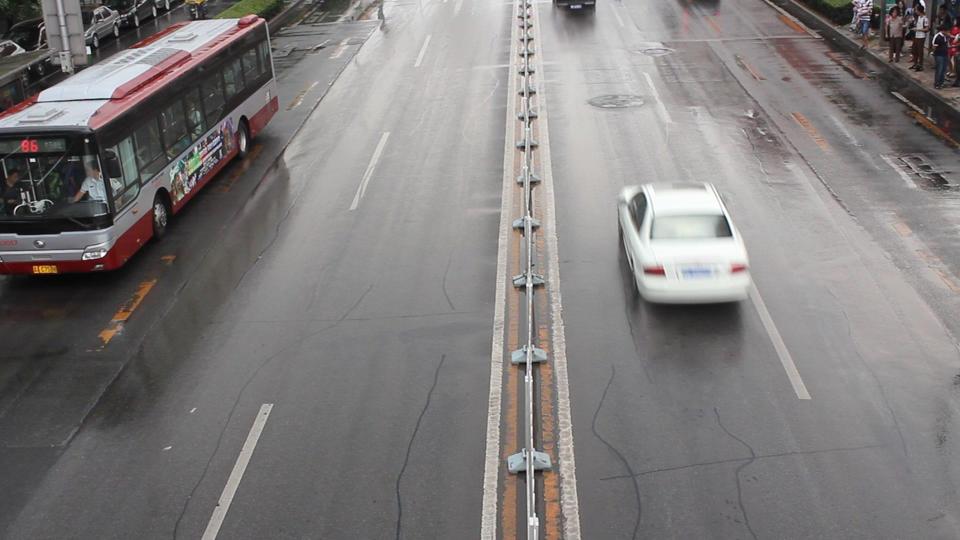

Supplement: Data S1 [file peerj-cs-09-1411-s001.zip › dataset/MVI_63525_img00494.jpg]

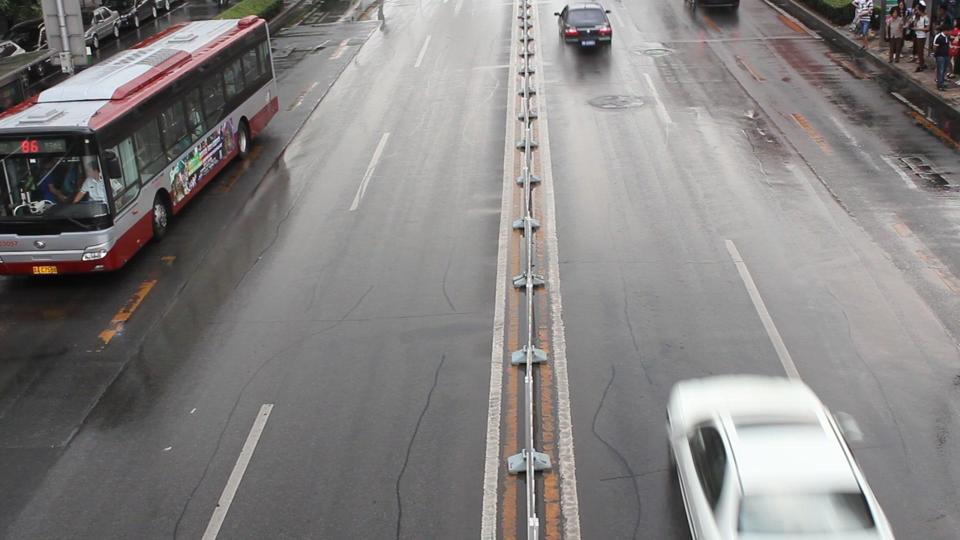

Supplement: Data S1 [file peerj-cs-09-1411-s001.zip › dataset/MVI_63525_img00480.jpg]

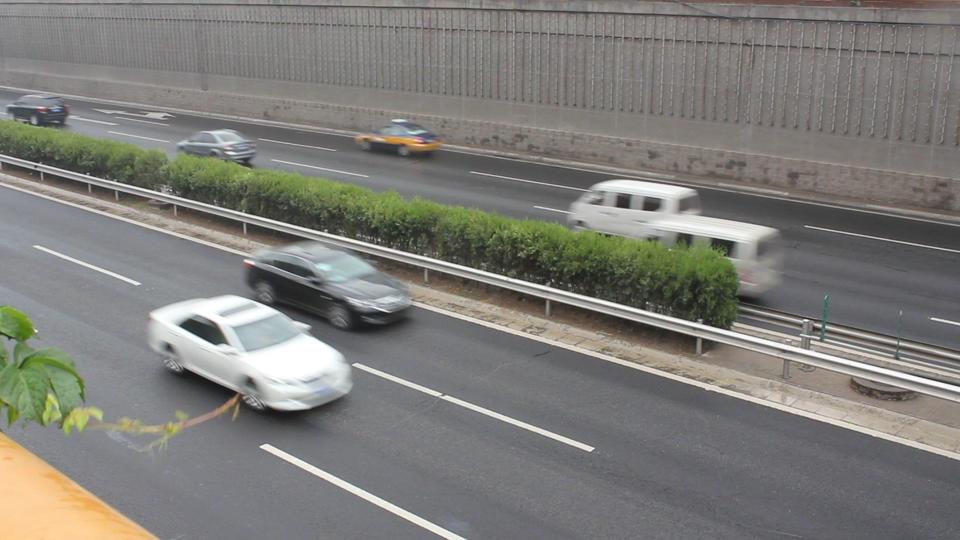

Supplement: Data S1 [file peerj-cs-09-1411-s001.zip › dataset/MVI_63554_img00124.jpg]

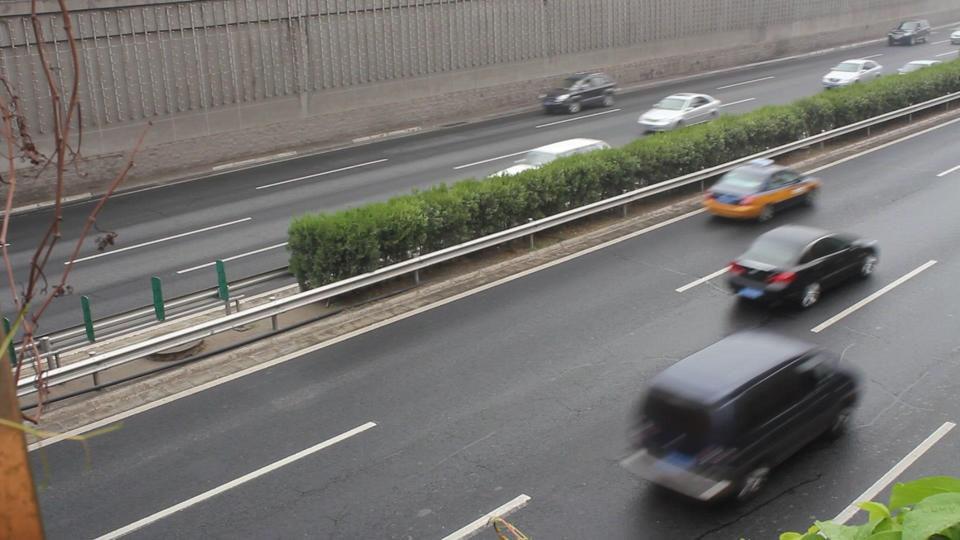

Supplement: Data S1 [file peerj-cs-09-1411-s001.zip › dataset/MVI_63563_img00135.jpg]

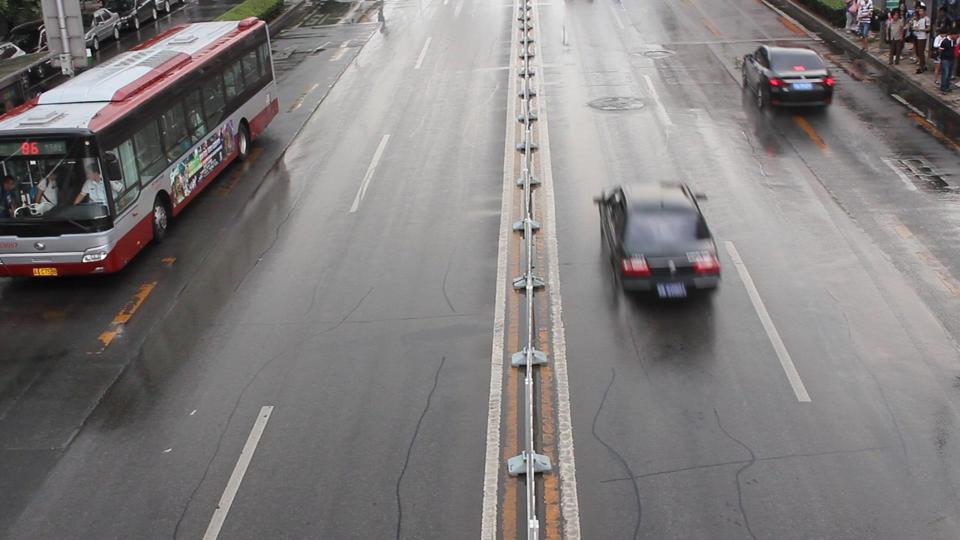

Supplement: Data S1 [file peerj-cs-09-1411-s001.zip › dataset/MVI_63525_img00443.jpg]

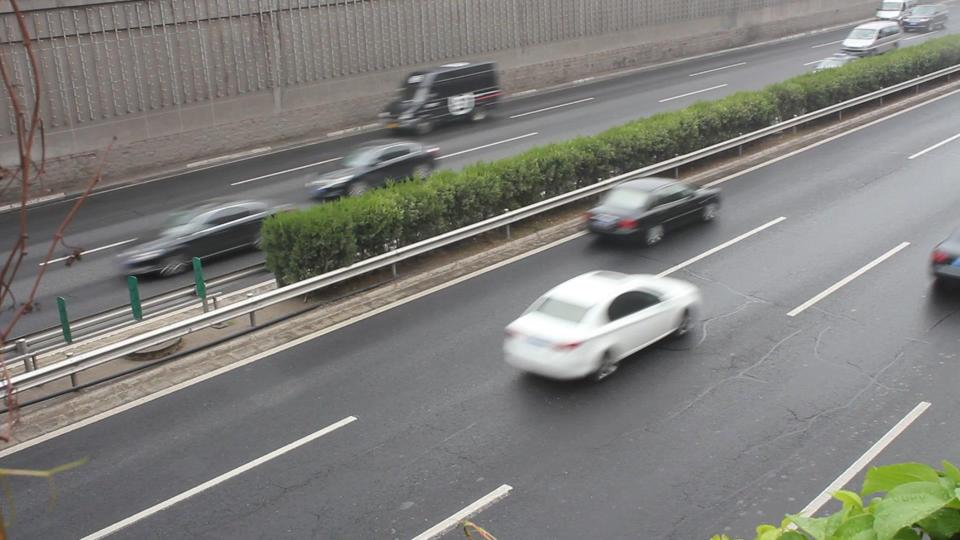

Supplement: Data S1 [file peerj-cs-09-1411-s001.zip › dataset/MVI_63562_img00085.jpg]

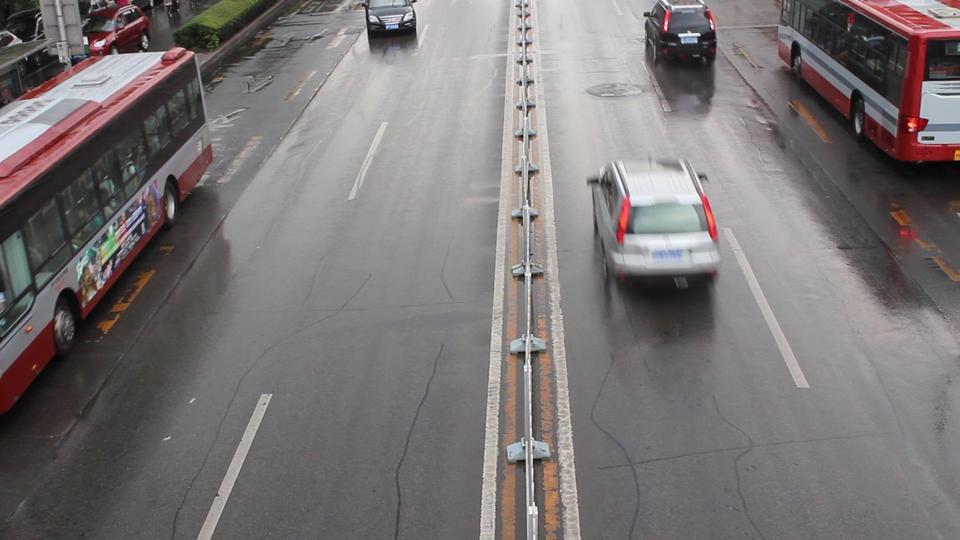

Supplement: Data S1 [file peerj-cs-09-1411-s001.zip › dataset/MVI_63525_img00872.jpg]

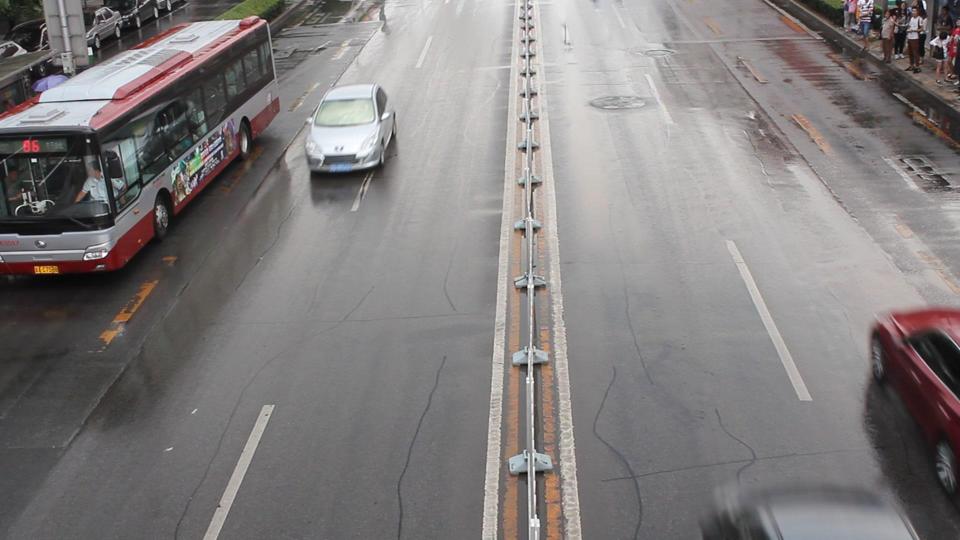

Supplement: Data S1 [file peerj-cs-09-1411-s001.zip › dataset/MVI_63525_img00286.jpg]

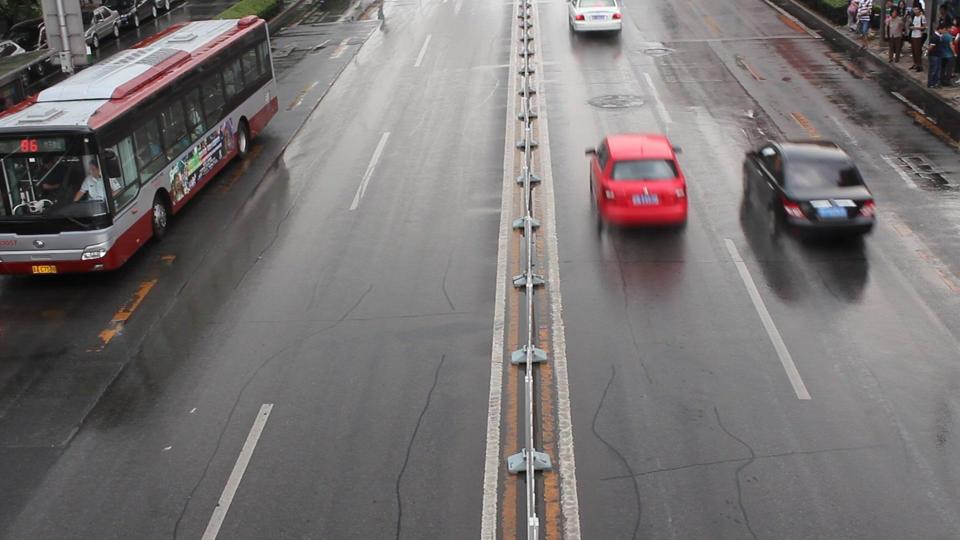

Supplement: Data S1 [file peerj-cs-09-1411-s001.zip › dataset/MVI_63525_img00536.jpg]

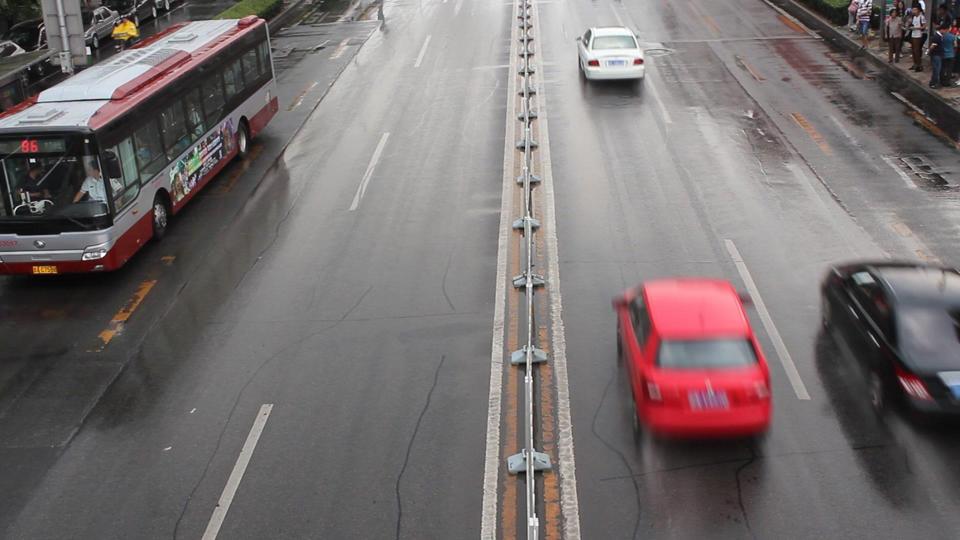

Supplement: Data S1 [file peerj-cs-09-1411-s001.zip › dataset/MVI_63525_img00522.jpg]

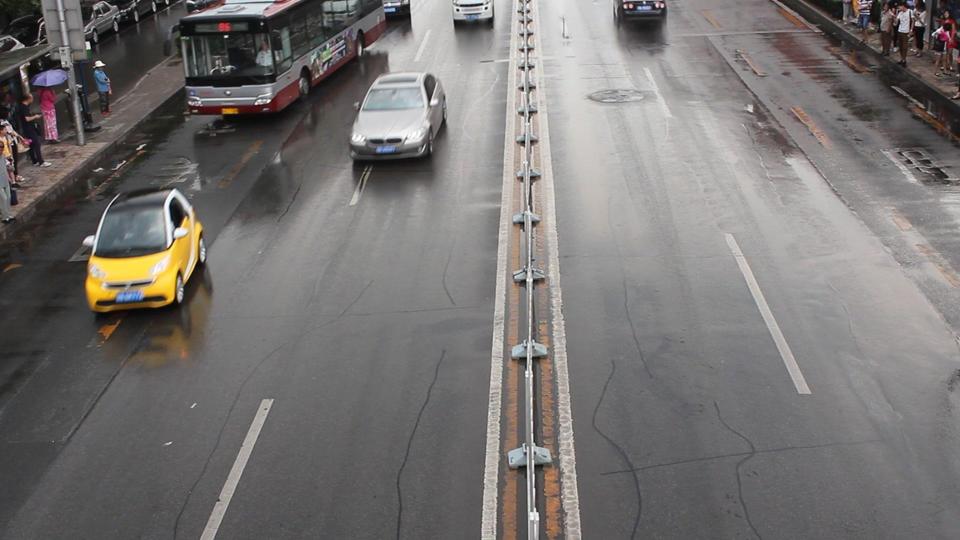

Supplement: Data S1 [file peerj-cs-09-1411-s001.zip › dataset/MVI_63525_img00046.jpg]

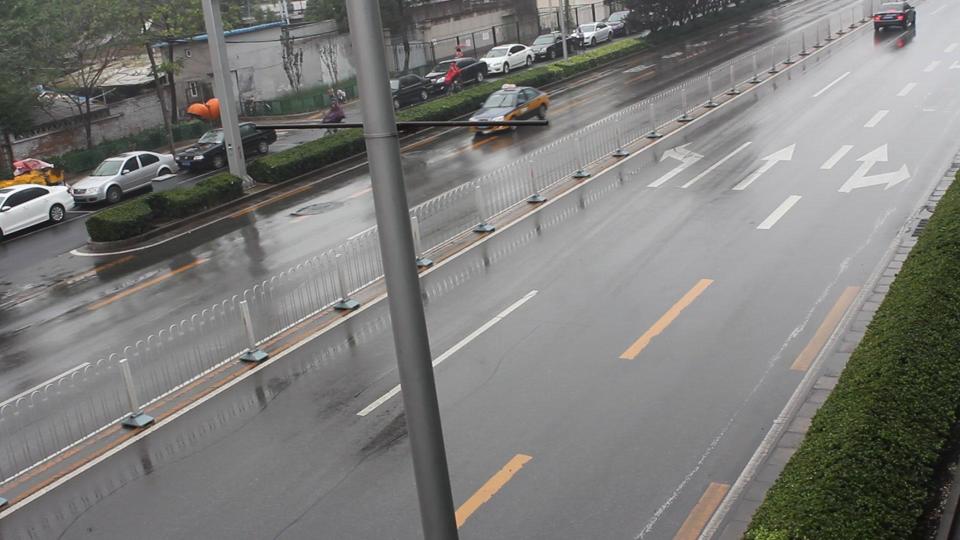

Supplement: Data S1 [file peerj-cs-09-1411-s001.zip › dataset/MVI_63544_img00671.jpg]

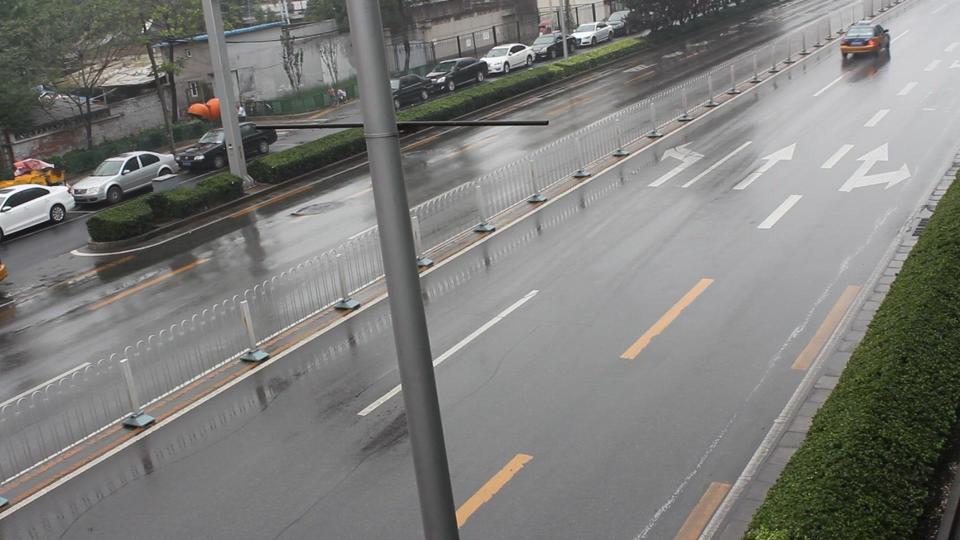

Supplement: Data S1 [file peerj-cs-09-1411-s001.zip › dataset/MVI_63544_img00881.jpg]

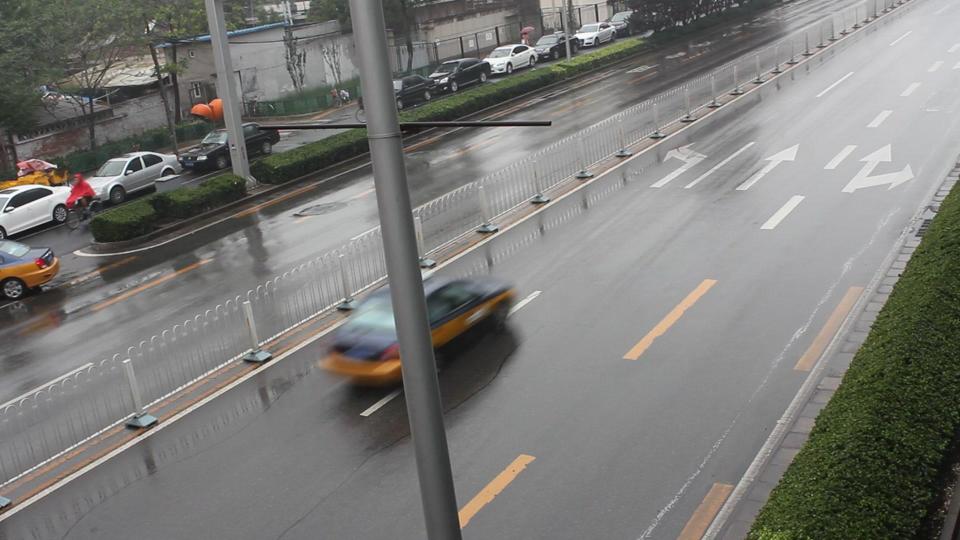

Supplement: Data S1 [file peerj-cs-09-1411-s001.zip › dataset/MVI_63544_img00825.jpg]

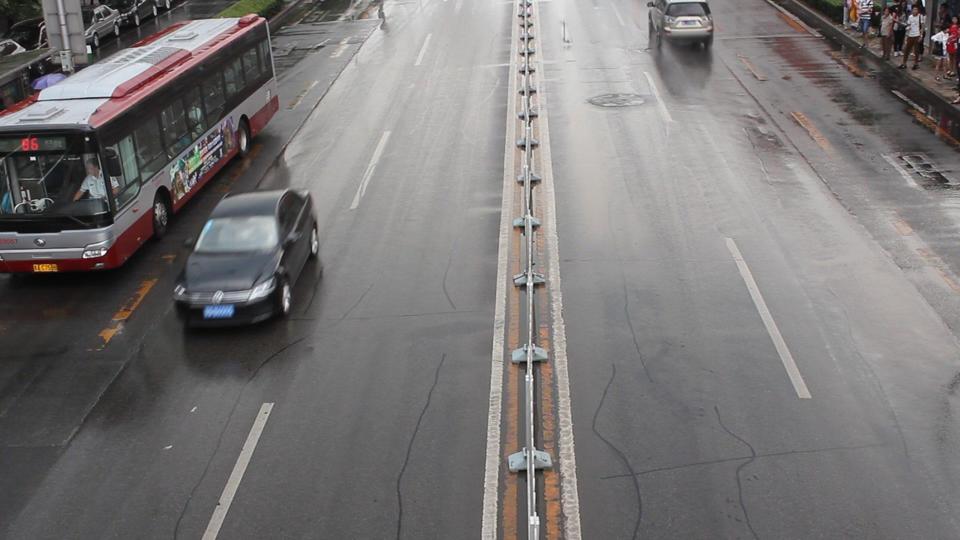

Supplement: Data S1 [file peerj-cs-09-1411-s001.zip › dataset/MVI_63525_img00169.jpg]

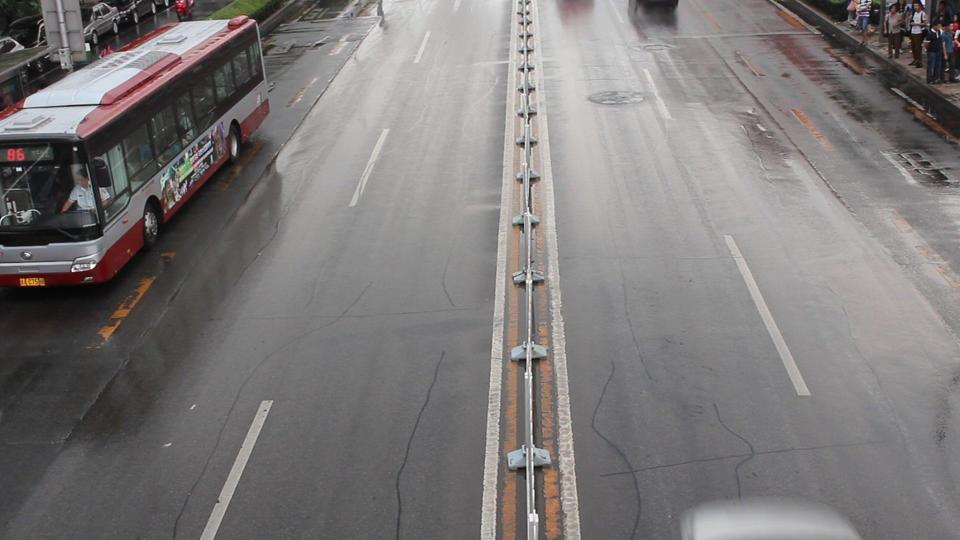

Supplement: Data S1 [file peerj-cs-09-1411-s001.zip › dataset/MVI_63525_img00592.jpg]

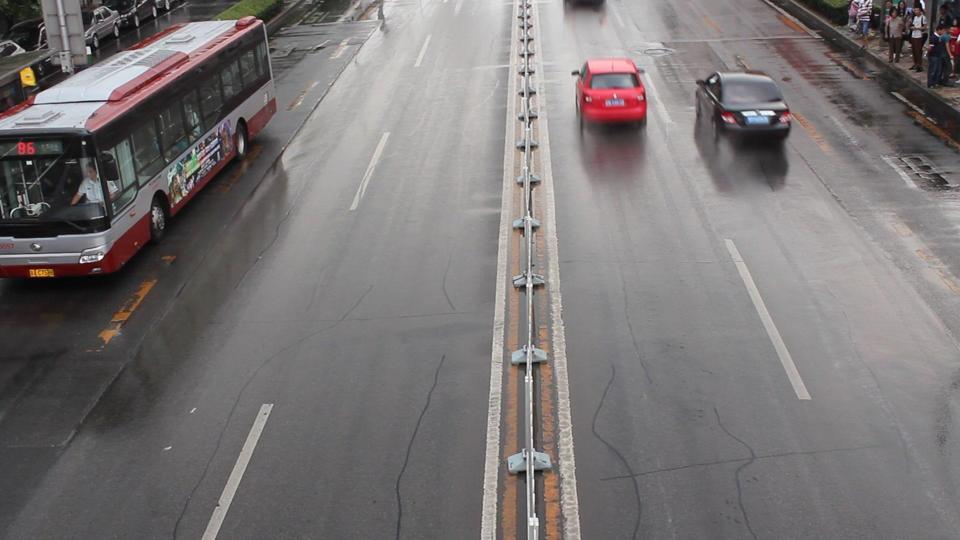

Supplement: Data S1 [file peerj-cs-09-1411-s001.zip › dataset/MVI_63525_img00550.jpg]

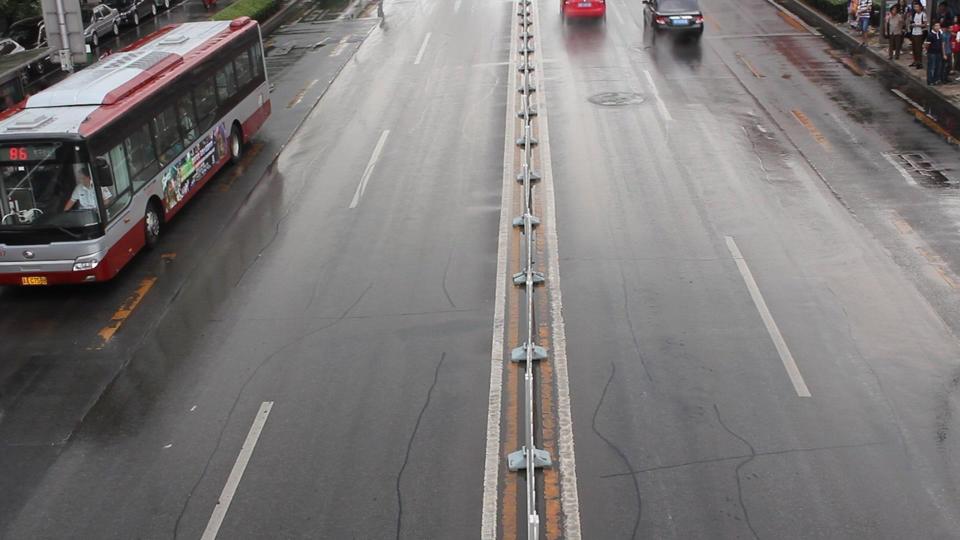

Supplement: Data S1 [file peerj-cs-09-1411-s001.zip › dataset/MVI_63525_img00578.jpg]

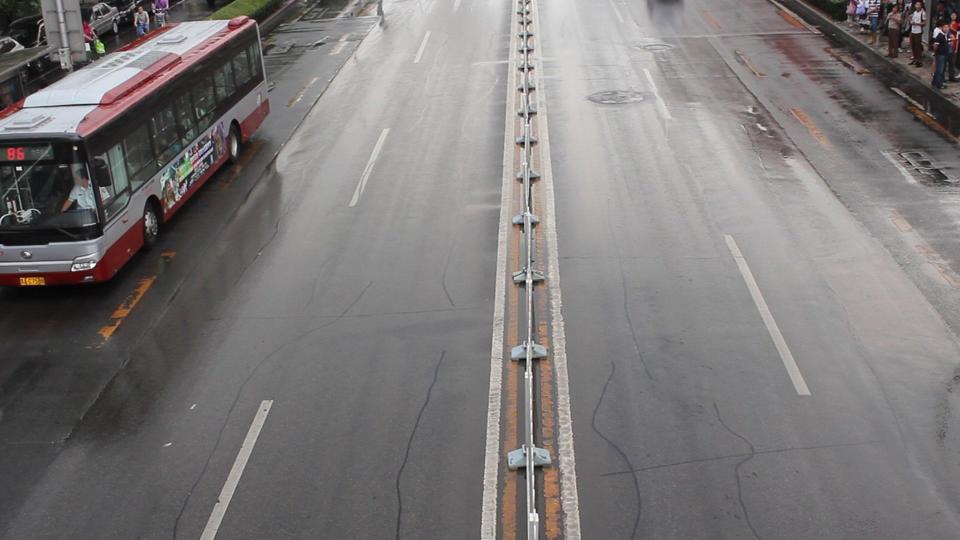

Supplement: Data S1 [file peerj-cs-09-1411-s001.zip › dataset/MVI_63525_img00746.jpg]

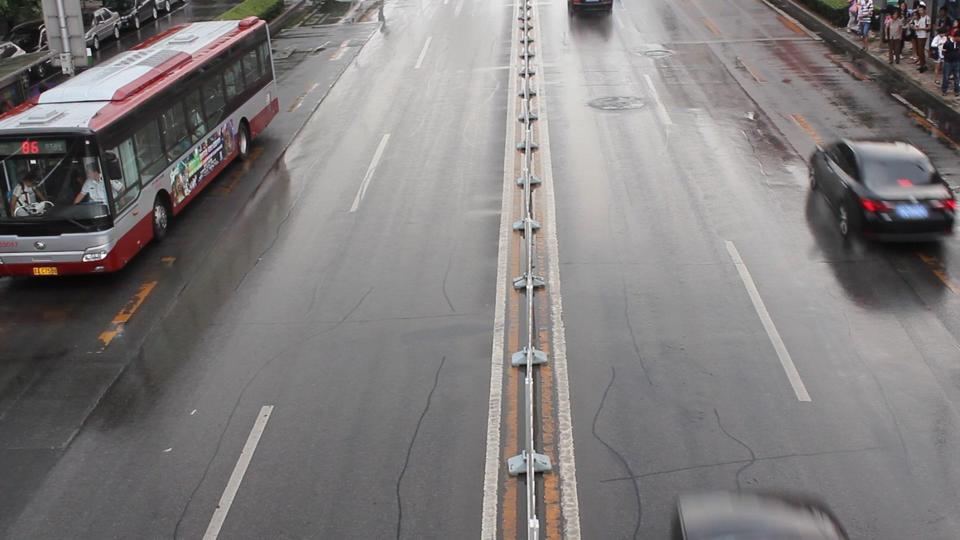

Supplement: Data S1 [file peerj-cs-09-1411-s001.zip › dataset/MVI_63525_img00424.jpg]

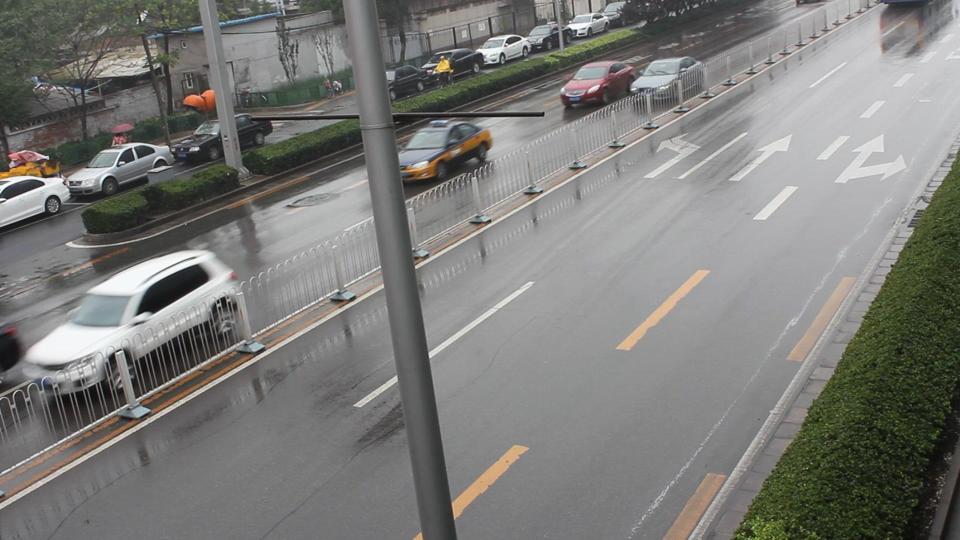

Supplement: Data S1 [file peerj-cs-09-1411-s001.zip › dataset/MVI_63544_img00167.jpg]

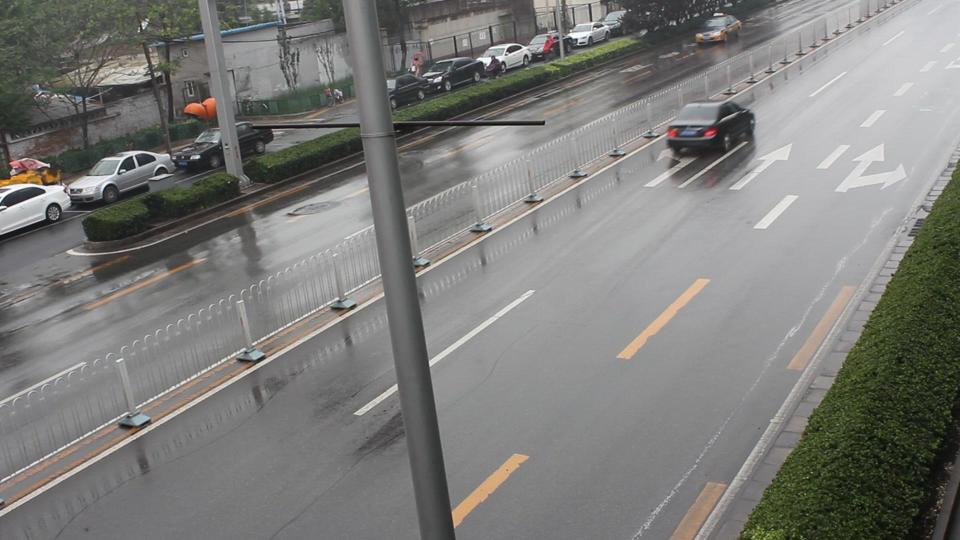

Supplement: Data S1 [file peerj-cs-09-1411-s001.zip › dataset/MVI_63544_img00601.jpg]

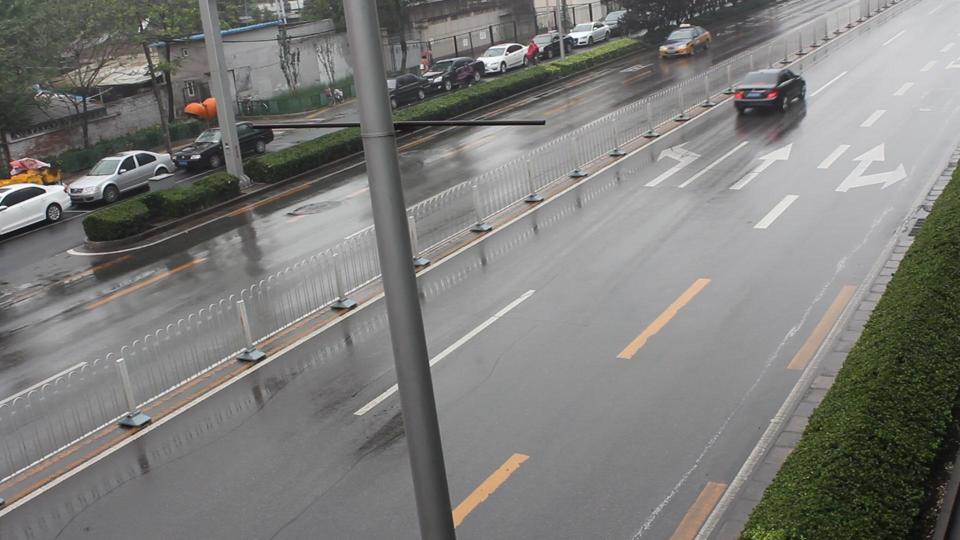

Supplement: Data S1 [file peerj-cs-09-1411-s001.zip › dataset/MVI_63544_img00615.jpg]

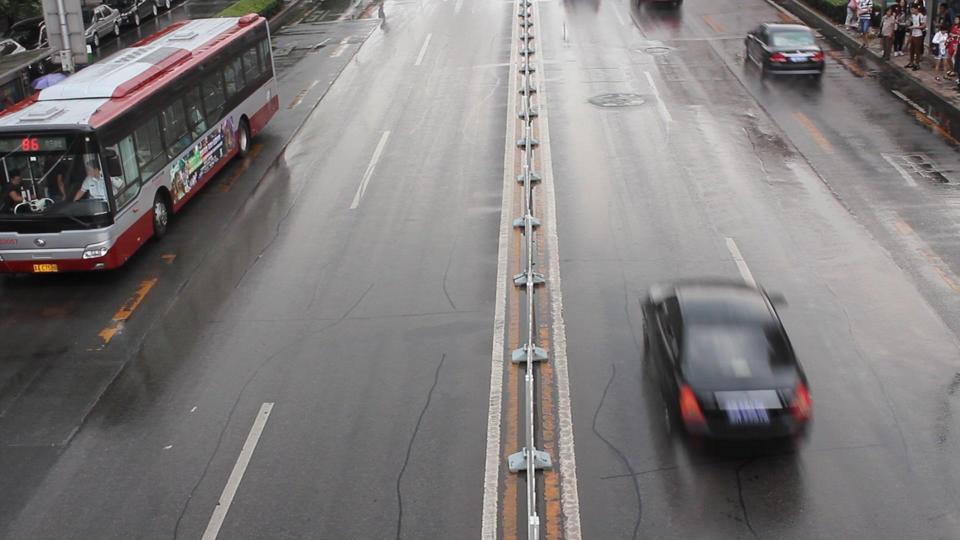

Supplement: Data S1 [file peerj-cs-09-1411-s001.zip › dataset/MVI_63525_img00368.jpg]
