# Supplementary figures and images for: A novel traffic optimization method using GRU based deep neural network for the IoV system (part 1 of 2)
Source: PeerJ Comput Sci. 2023 Jun 6;9:e1411. doi: 10.7717/peerj-cs.1411 (PMC10280423; doi:10.7717/peerj-cs.1411)

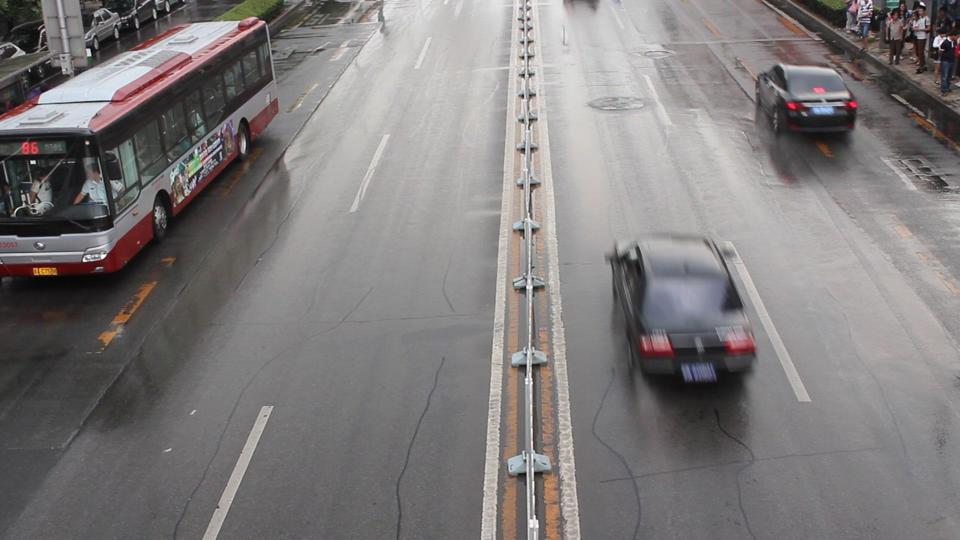

Supplement: Data S1 [file peerj-cs-09-1411-s001.zip › dataset/MVI_63525_img00438.jpg]

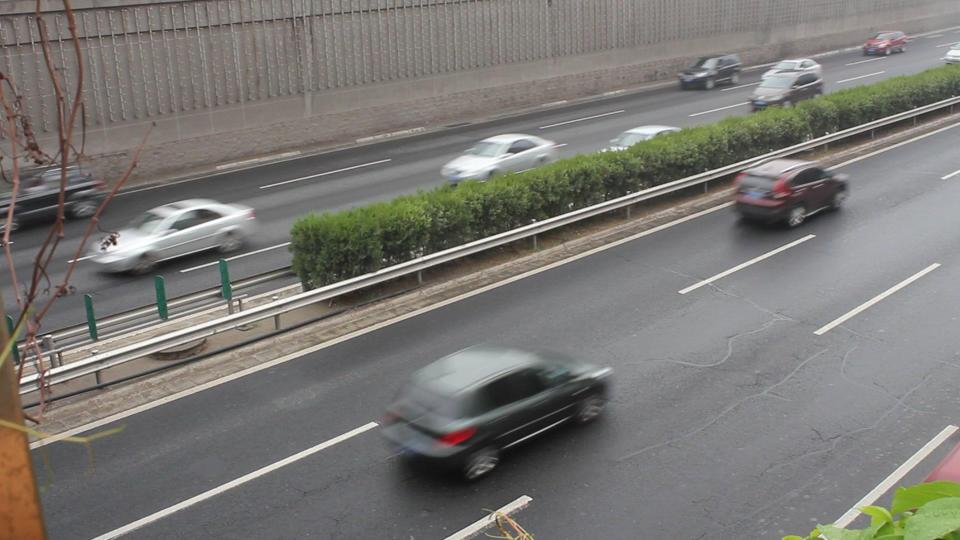

Supplement: Data S1 [file peerj-cs-09-1411-s001.zip › dataset/MVI_63563_img00172.jpg]

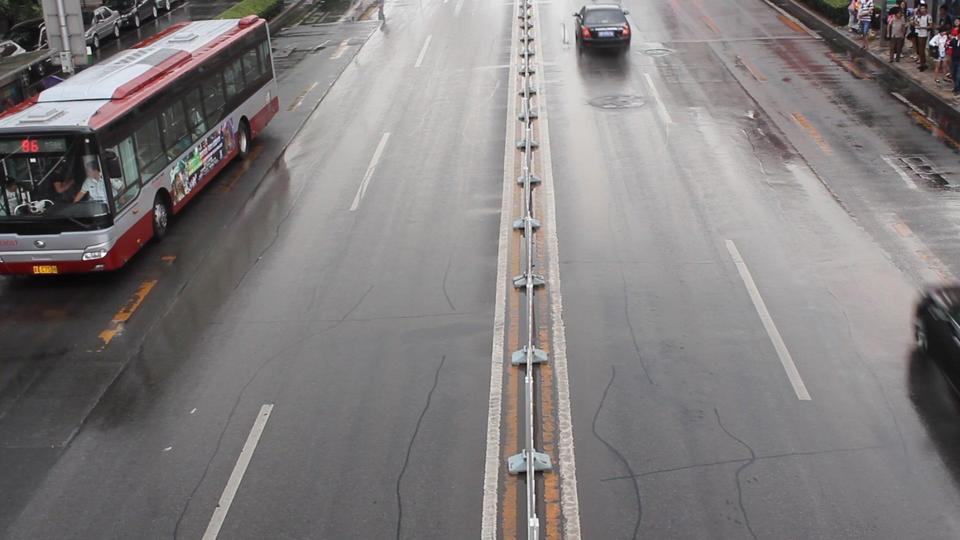

Supplement: Data S1 [file peerj-cs-09-1411-s001.zip › dataset/MVI_63525_img00410.jpg]

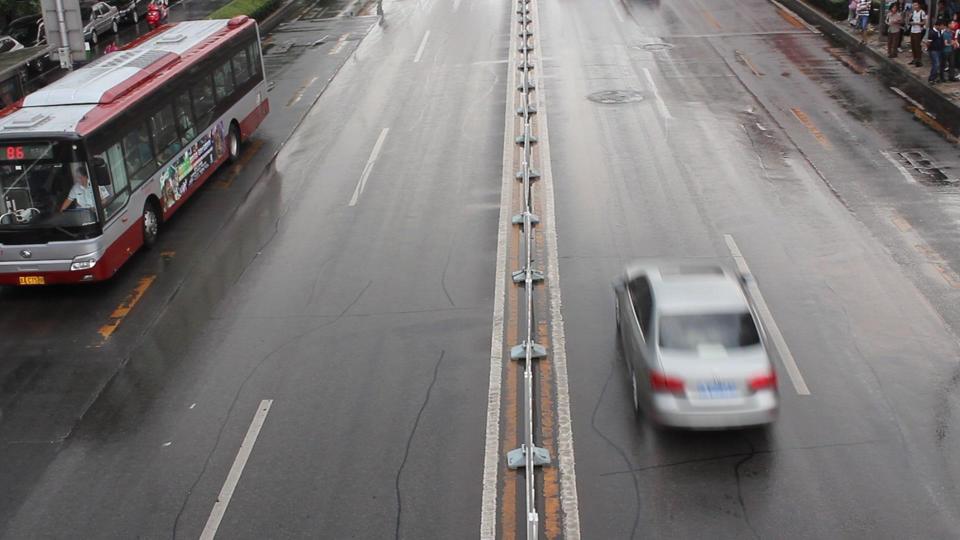

Supplement: Data S1 [file peerj-cs-09-1411-s001.zip › dataset/MVI_63525_img00606.jpg]

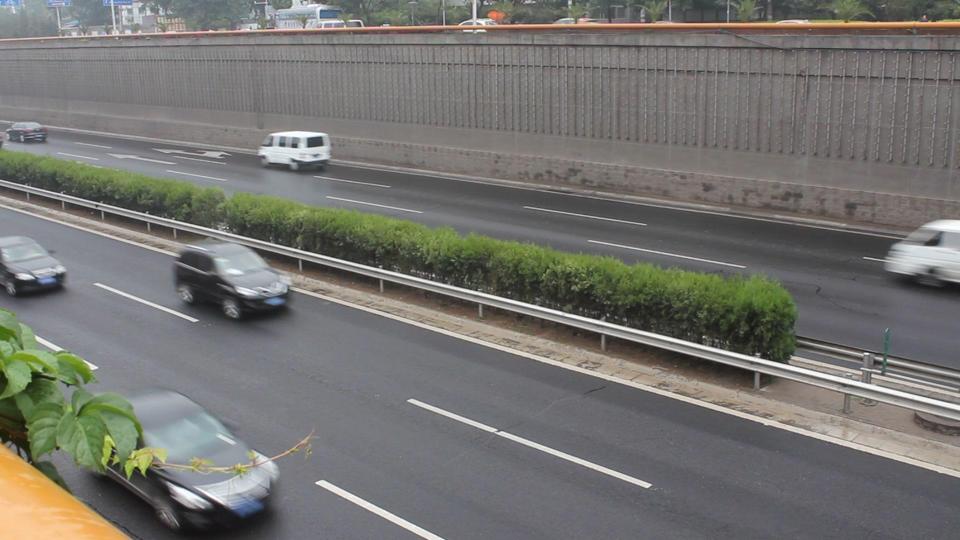

Supplement: Data S1 [file peerj-cs-09-1411-s001.zip › dataset/MVI_63552_img00399.jpg]

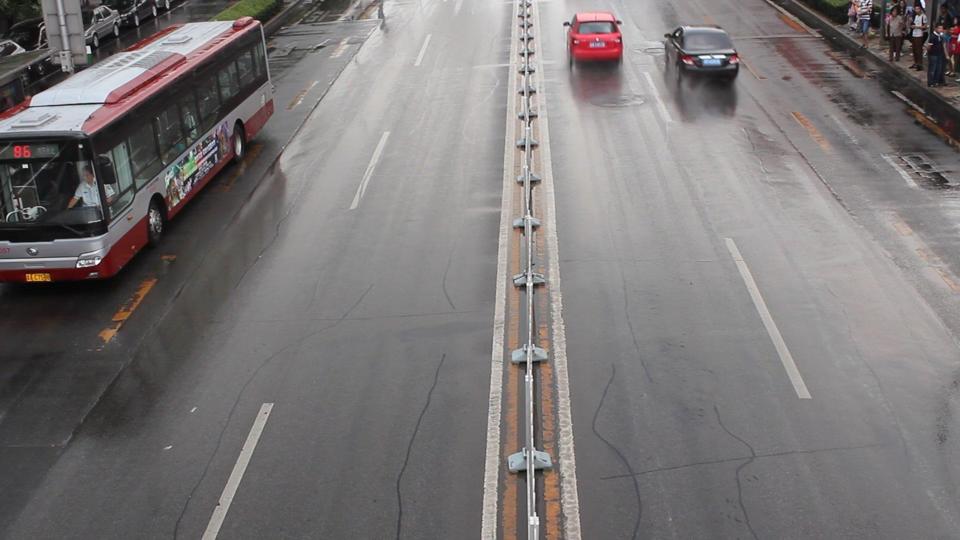

Supplement: Data S1 [file peerj-cs-09-1411-s001.zip › dataset/MVI_63525_img00564.jpg]

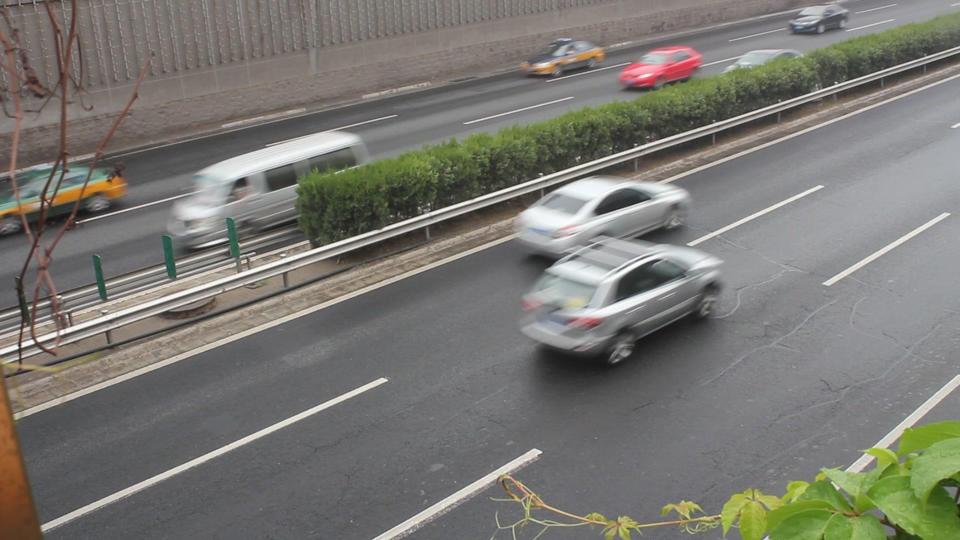

Supplement: Data S1 [file peerj-cs-09-1411-s001.zip › dataset/MVI_63563_img01318.jpg]

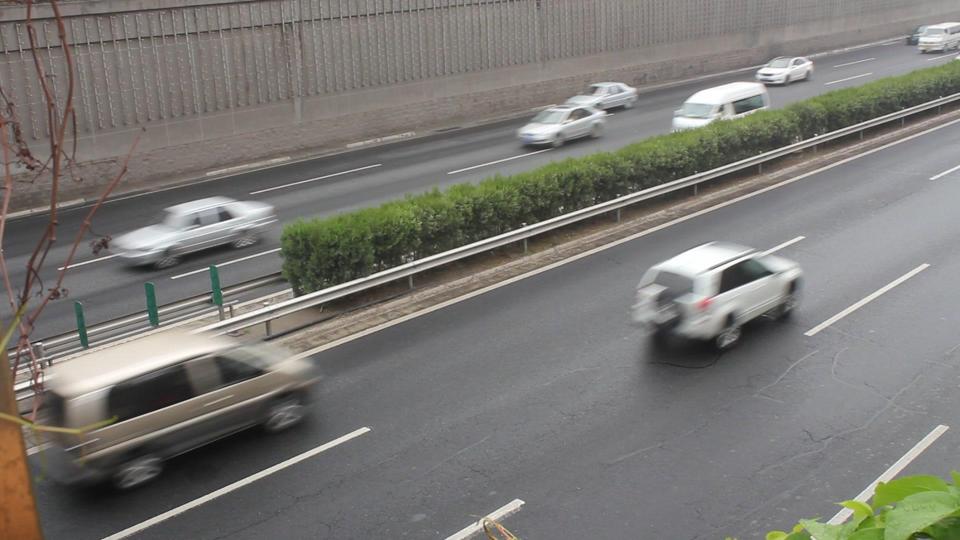

Supplement: Data S1 [file peerj-cs-09-1411-s001.zip › dataset/MVI_63563_img00577.jpg]

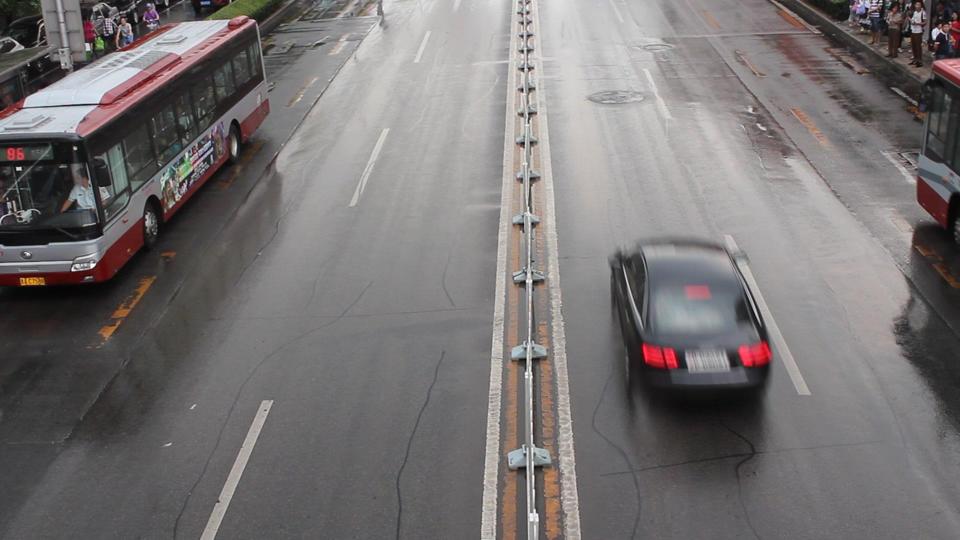

Supplement: Data S1 [file peerj-cs-09-1411-s001.zip › dataset/MVI_63525_img00767.jpg]

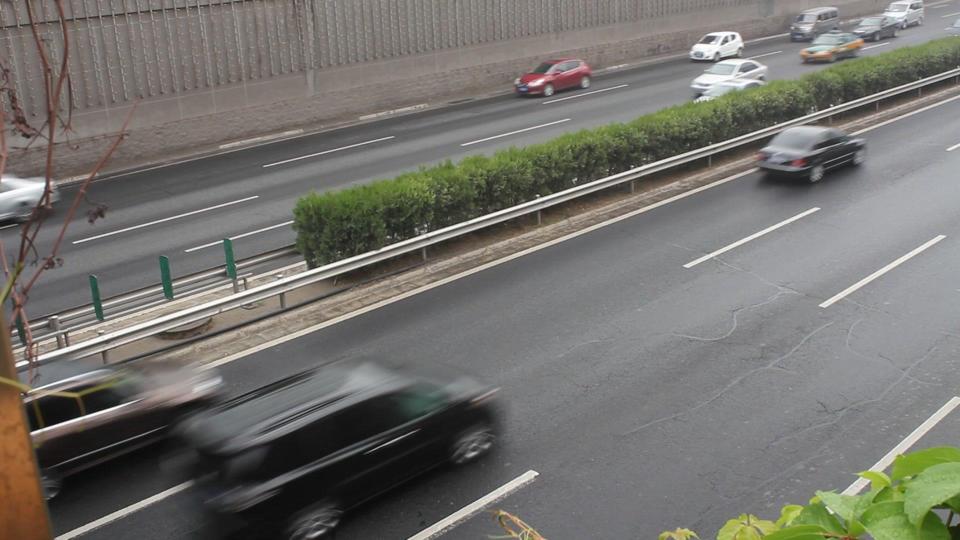

Supplement: Data S1 [file peerj-cs-09-1411-s001.zip › dataset/MVI_63563_img01084.jpg]

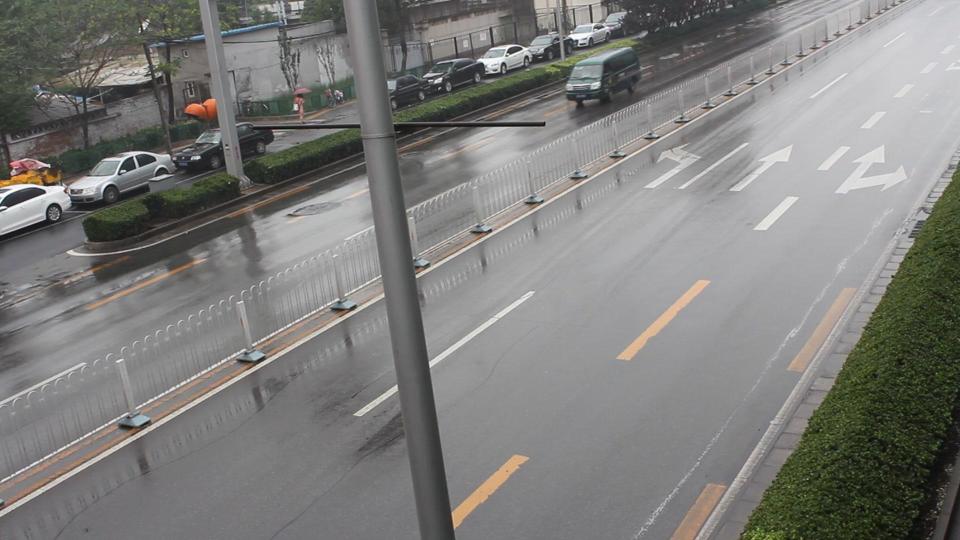

Supplement: Data S1 [file peerj-cs-09-1411-s001.zip › dataset/MVI_63544_img00391.jpg]

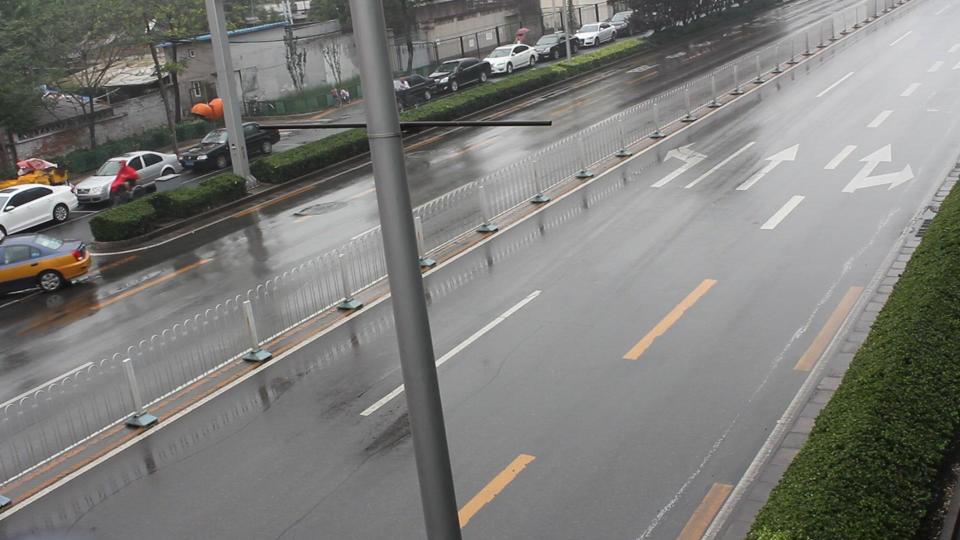

Supplement: Data S1 [file peerj-cs-09-1411-s001.zip › dataset/MVI_63544_img00811.jpg]

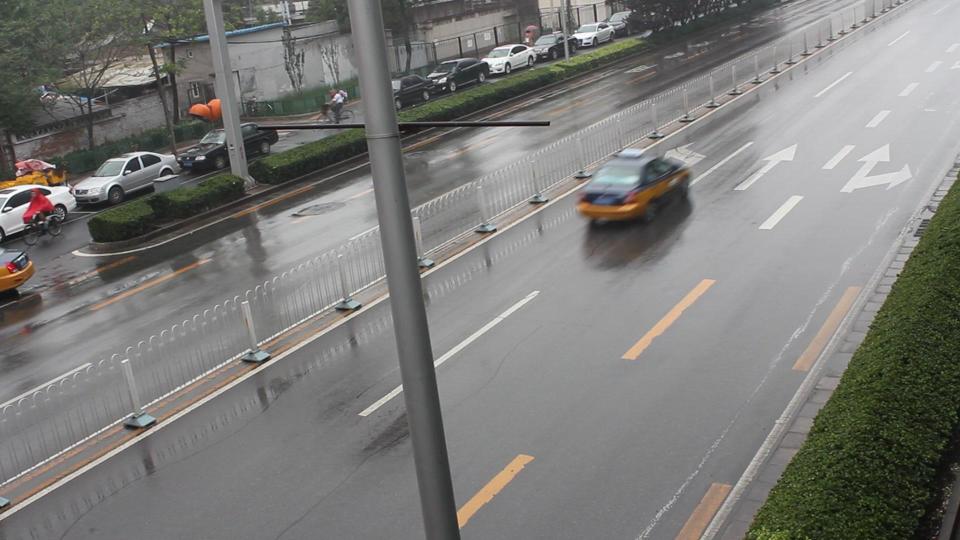

Supplement: Data S1 [file peerj-cs-09-1411-s001.zip › dataset/MVI_63544_img00839.jpg]

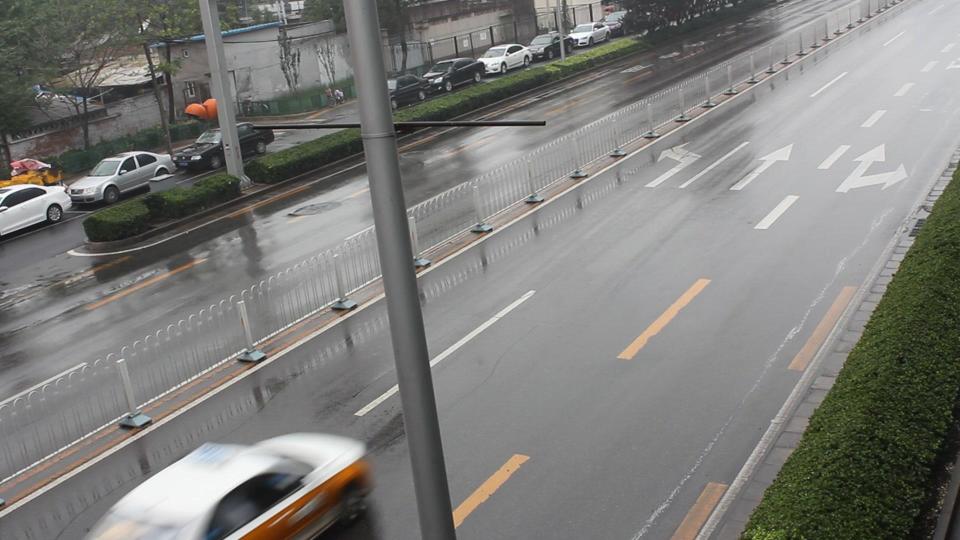

Supplement: Data S1 [file peerj-cs-09-1411-s001.zip › dataset/MVI_63544_img01099.jpg]

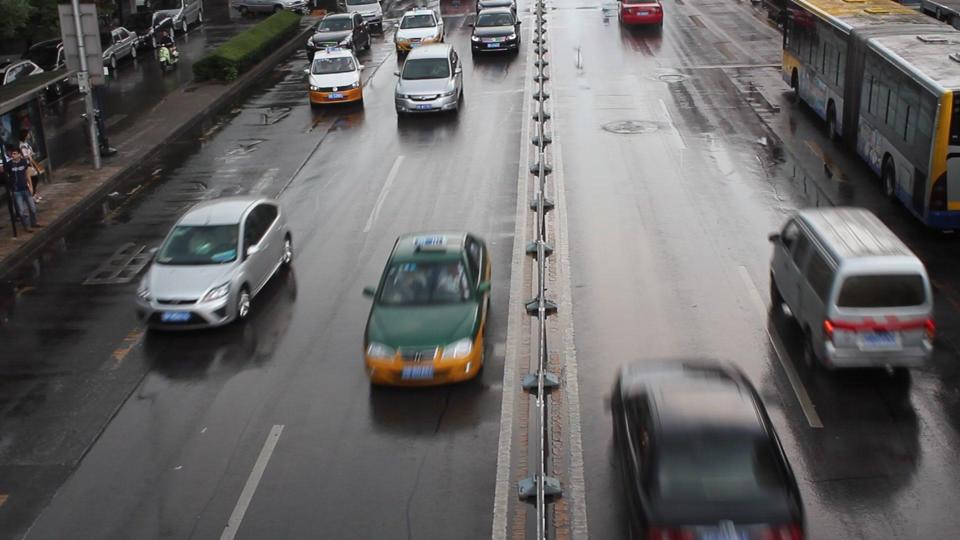

Supplement: Data S1 [file peerj-cs-09-1411-s001.zip › dataset/MVI_63521_img01261.jpg]

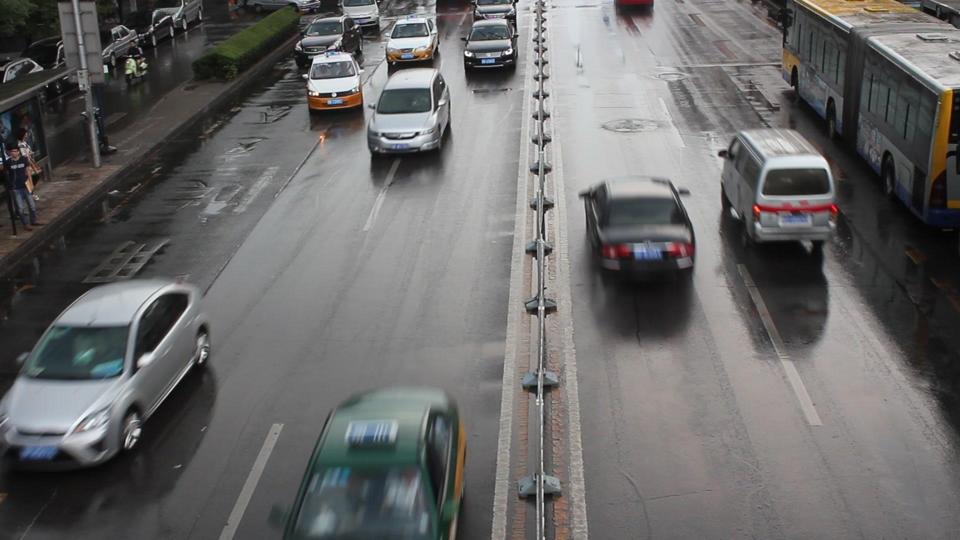

Supplement: Data S1 [file peerj-cs-09-1411-s001.zip › dataset/MVI_63521_img01275.jpg]

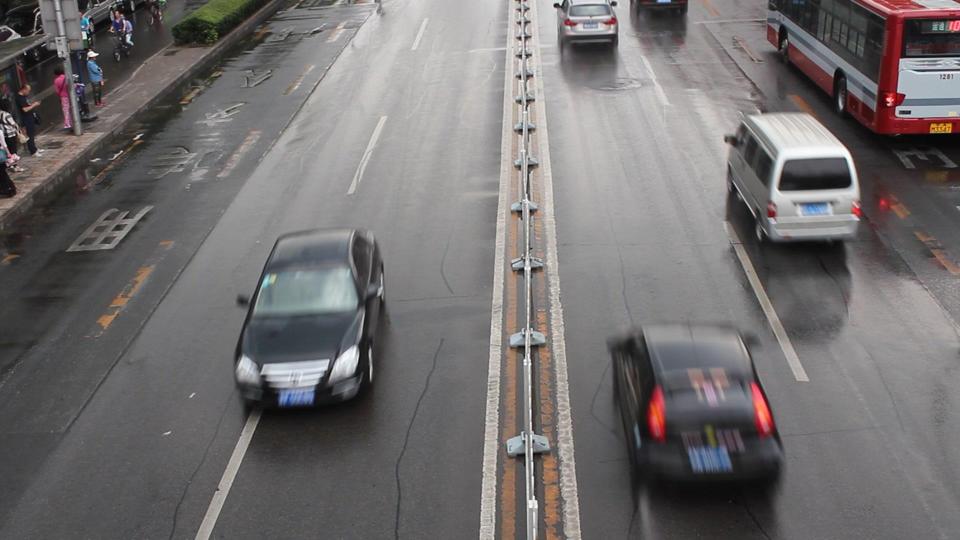

Supplement: Data S1 [file peerj-cs-09-1411-s001.zip › dataset/MVI_63525_img00956.jpg]

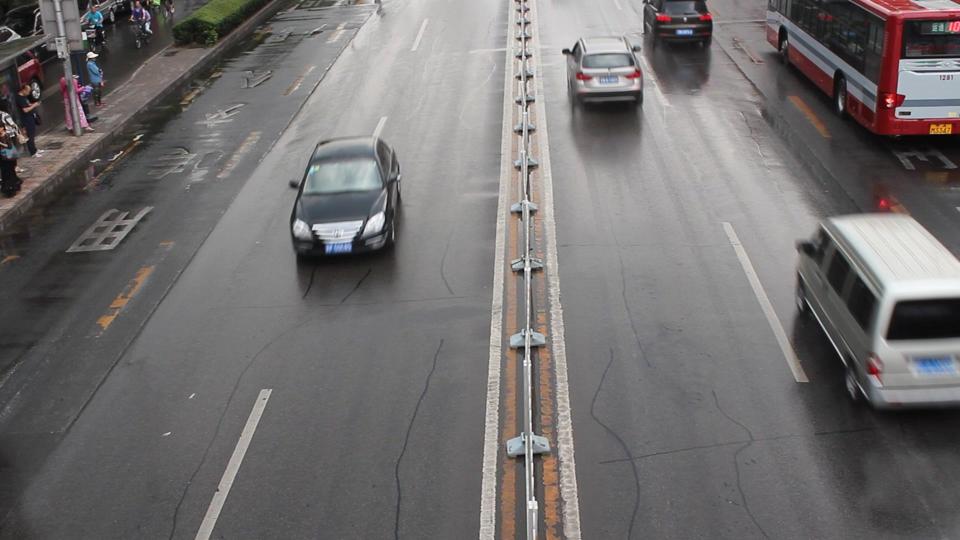

Supplement: Data S1 [file peerj-cs-09-1411-s001.zip › dataset/MVI_63525_img00942.jpg]

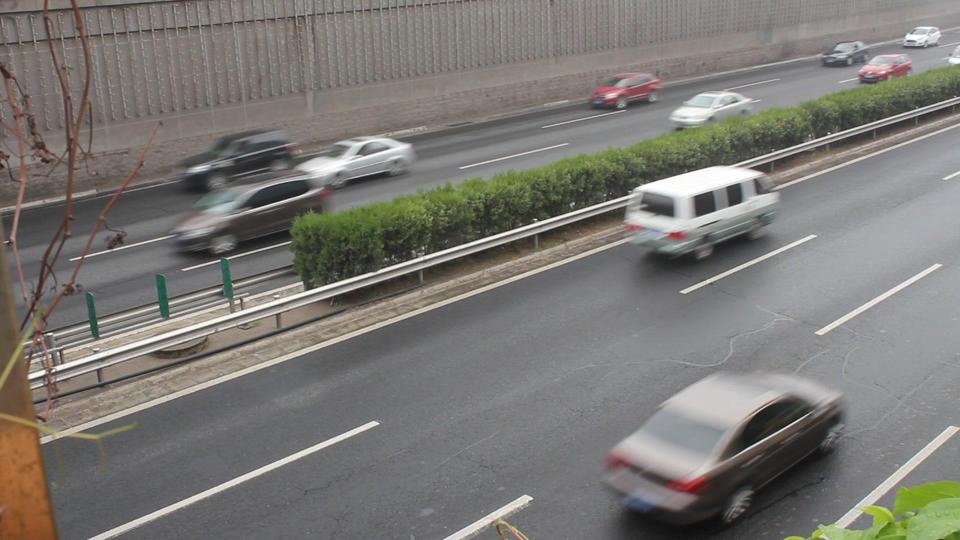

Supplement: Data S1 [file peerj-cs-09-1411-s001.zip › dataset/MVI_63563_img00213.jpg]

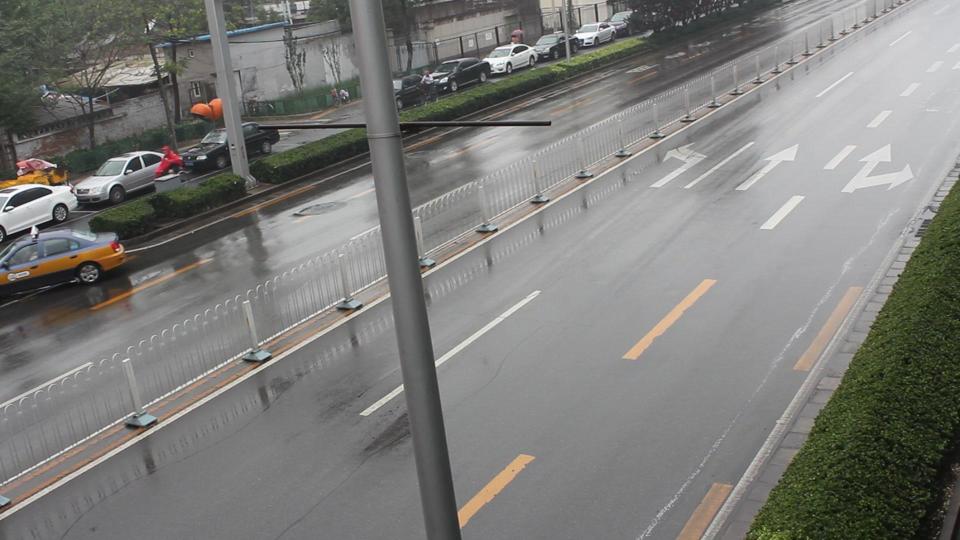

Supplement: Data S1 [file peerj-cs-09-1411-s001.zip › dataset/MVI_63544_img00797.jpg]

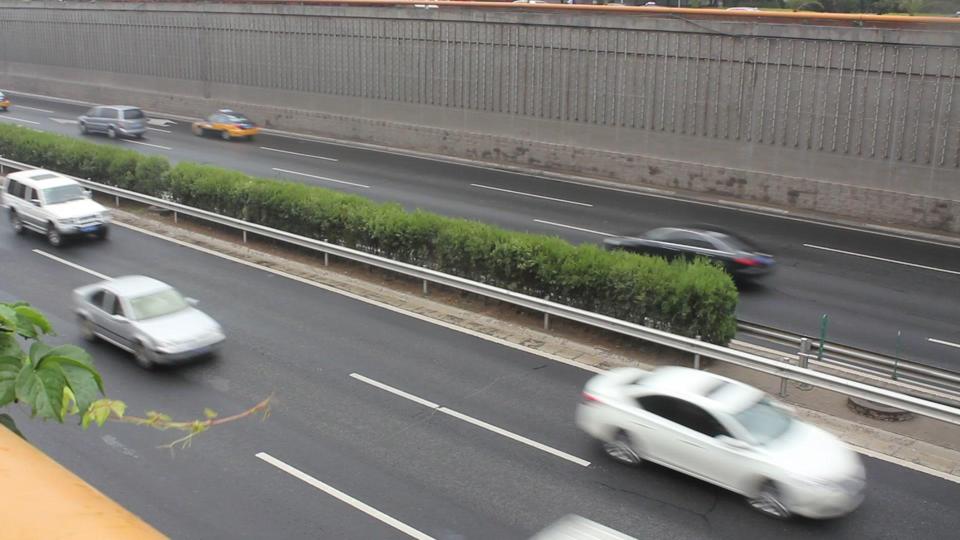

Supplement: Data S1 [file peerj-cs-09-1411-s001.zip › dataset/MVI_63554_img00982.jpg]

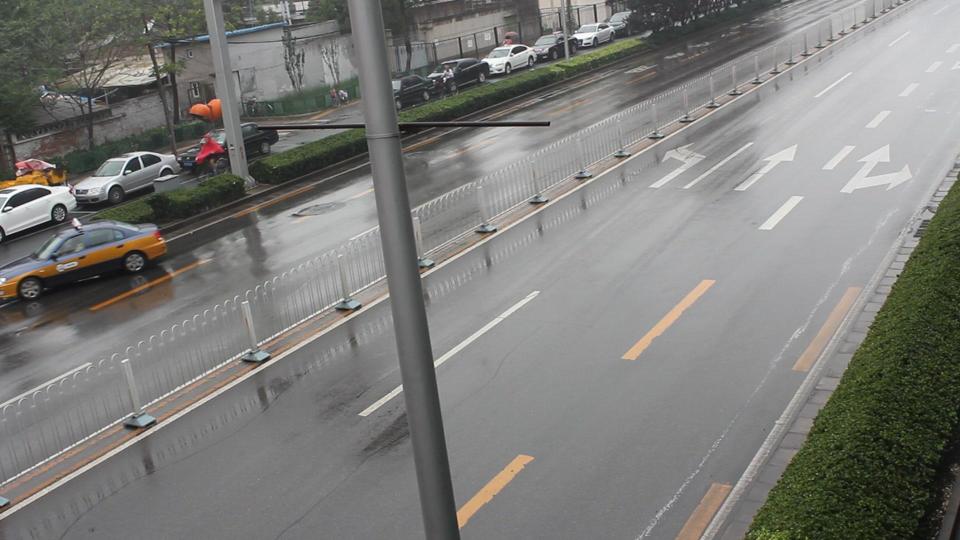

Supplement: Data S1 [file peerj-cs-09-1411-s001.zip › dataset/MVI_63544_img00783.jpg]

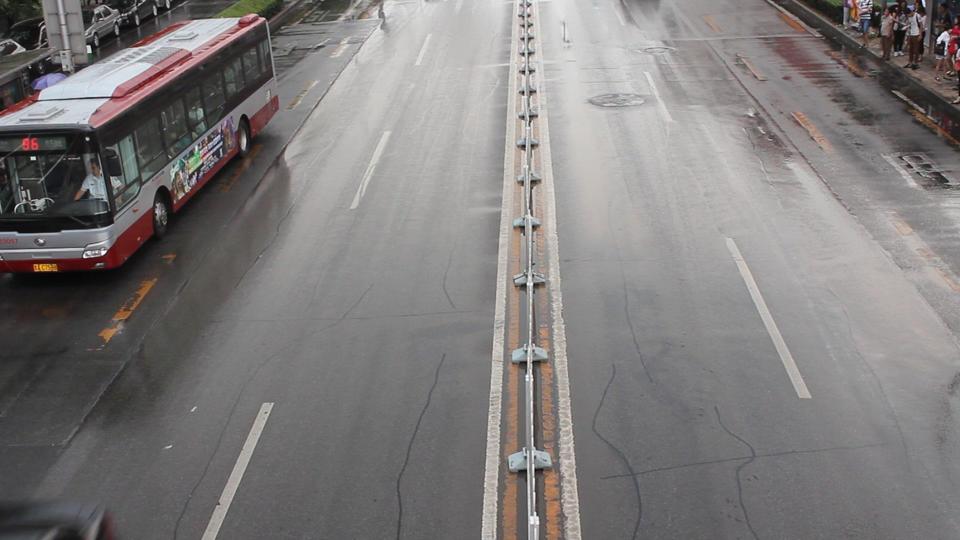

Supplement: Data S1 [file peerj-cs-09-1411-s001.zip › dataset/MVI_63525_img00200.jpg]

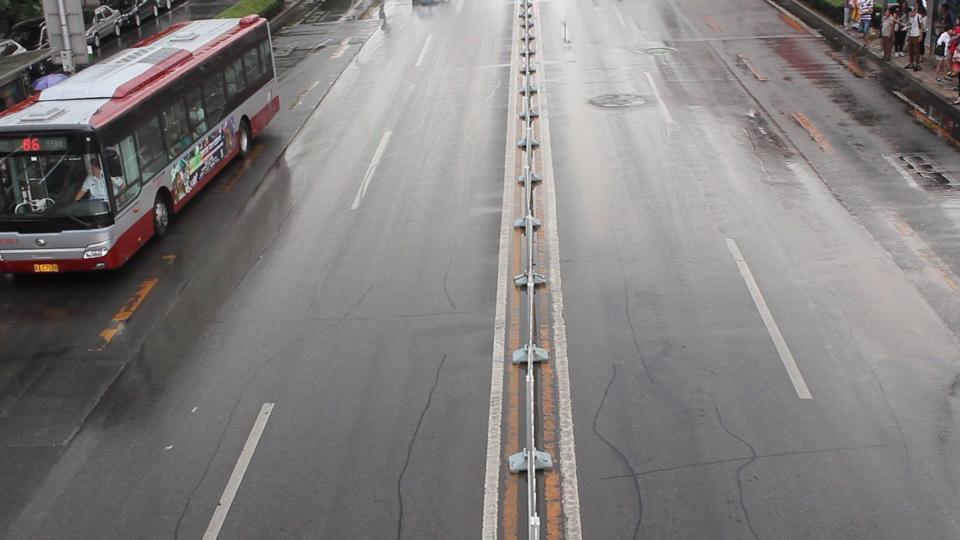

Supplement: Data S1 [file peerj-cs-09-1411-s001.zip › dataset/MVI_63525_img00214.jpg]

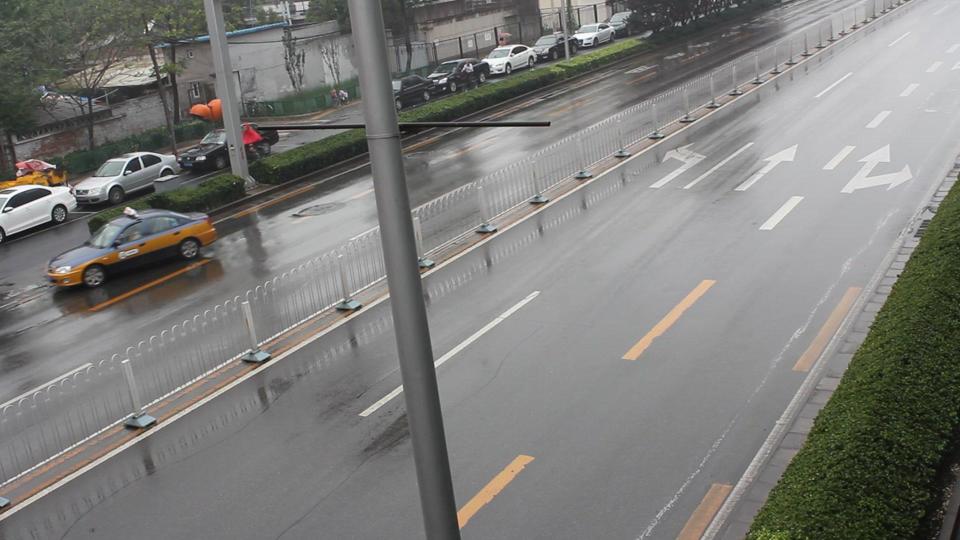

Supplement: Data S1 [file peerj-cs-09-1411-s001.zip › dataset/MVI_63544_img00769.jpg]

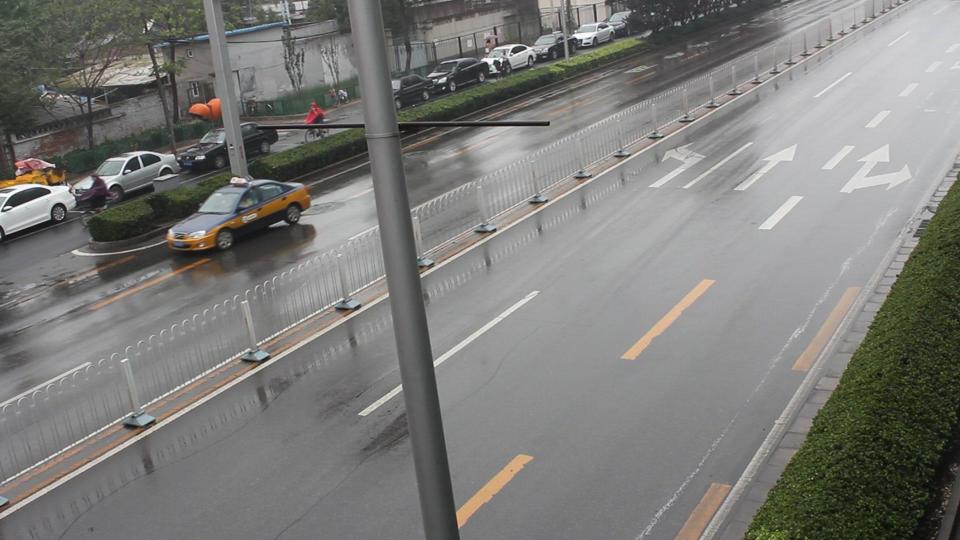

Supplement: Data S1 [file peerj-cs-09-1411-s001.zip › dataset/MVI_63544_img00741.jpg]

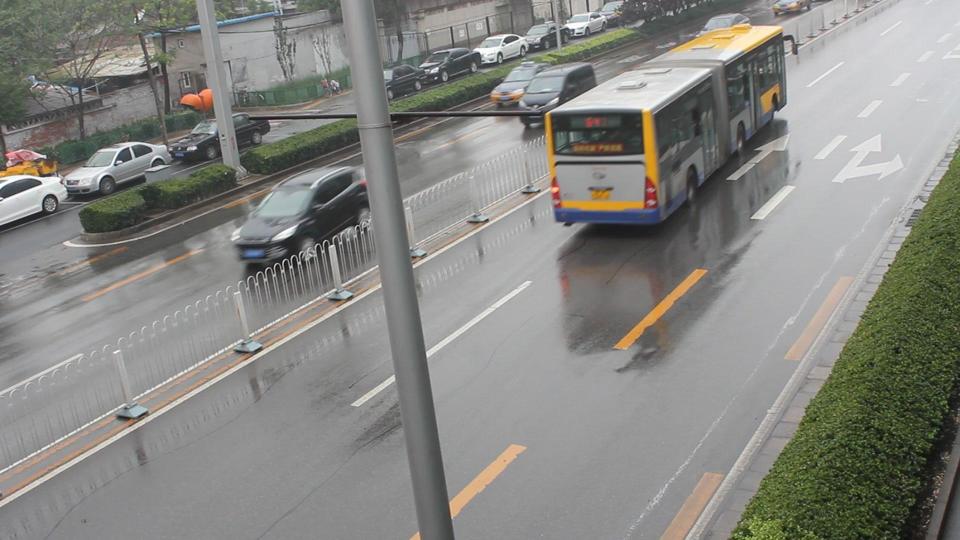

Supplement: Data S1 [file peerj-cs-09-1411-s001.zip › dataset/MVI_63544_img00027.jpg]

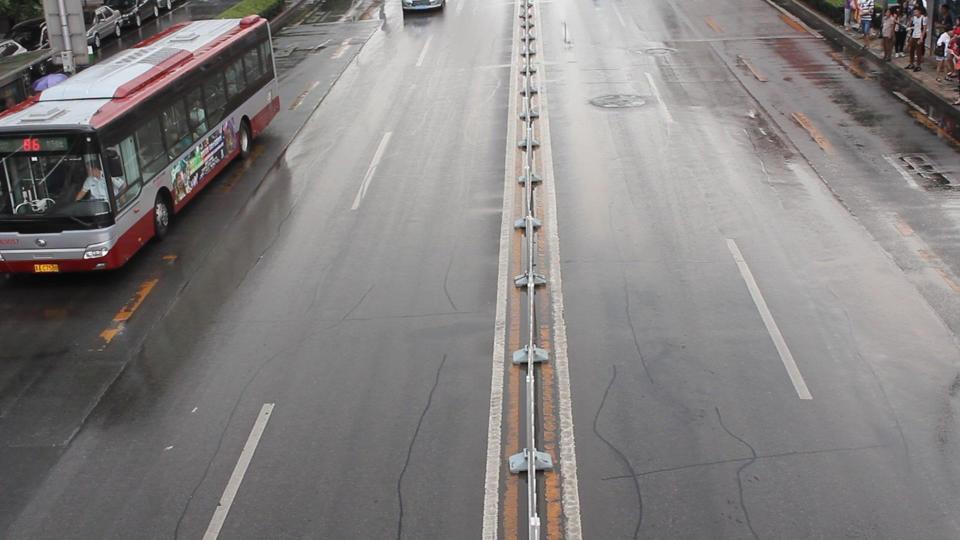

Supplement: Data S1 [file peerj-cs-09-1411-s001.zip › dataset/MVI_63525_img00228.jpg]

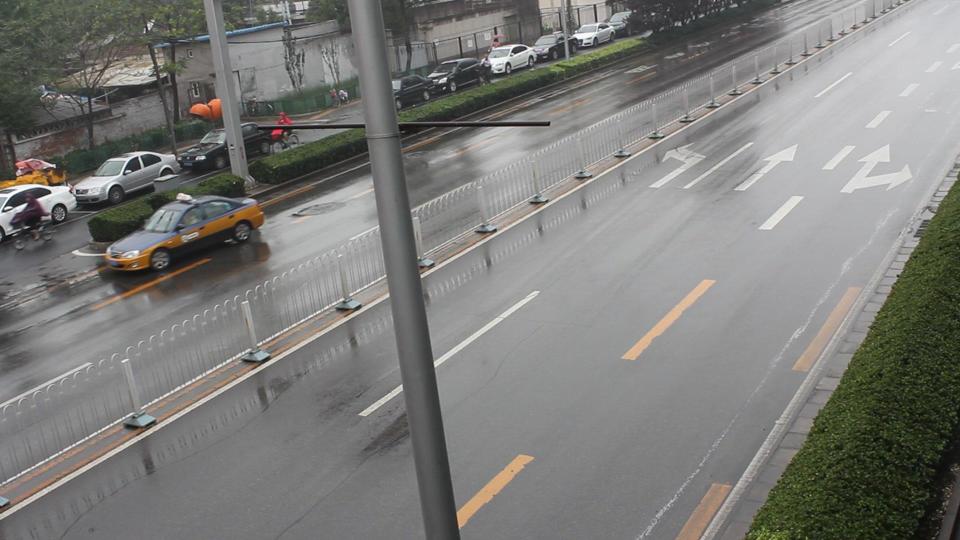

Supplement: Data S1 [file peerj-cs-09-1411-s001.zip › dataset/MVI_63544_img00755.jpg]

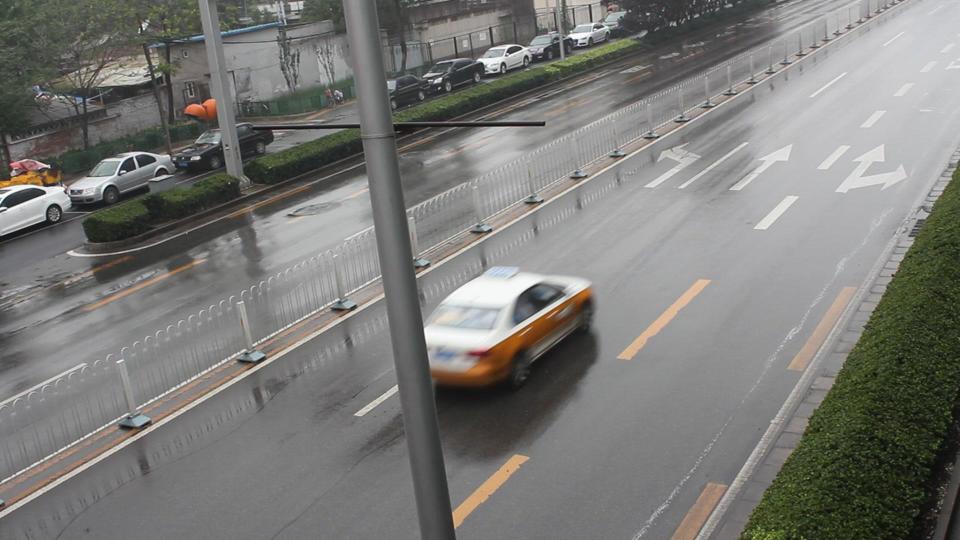

Supplement: Data S1 [file peerj-cs-09-1411-s001.zip › dataset/MVI_63544_img01113.jpg]

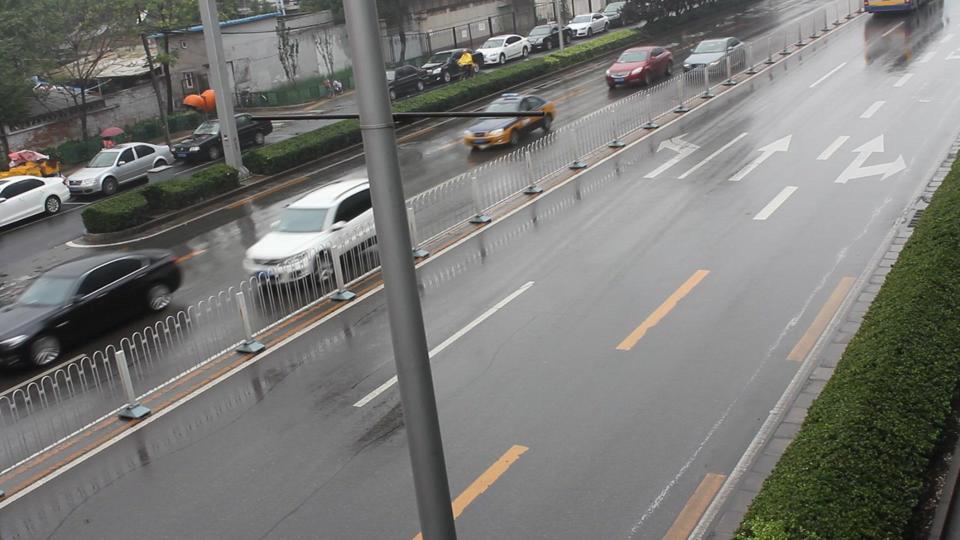

Supplement: Data S1 [file peerj-cs-09-1411-s001.zip › dataset/MVI_63544_img00153.jpg]

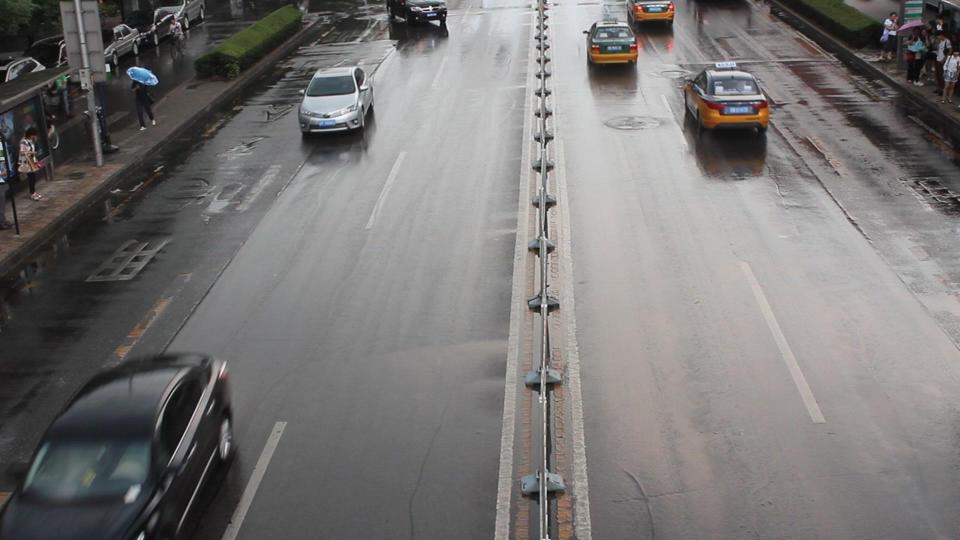

Supplement: Data S1 [file peerj-cs-09-1411-s001.zip › dataset/MVI_63521_img01933.jpg]

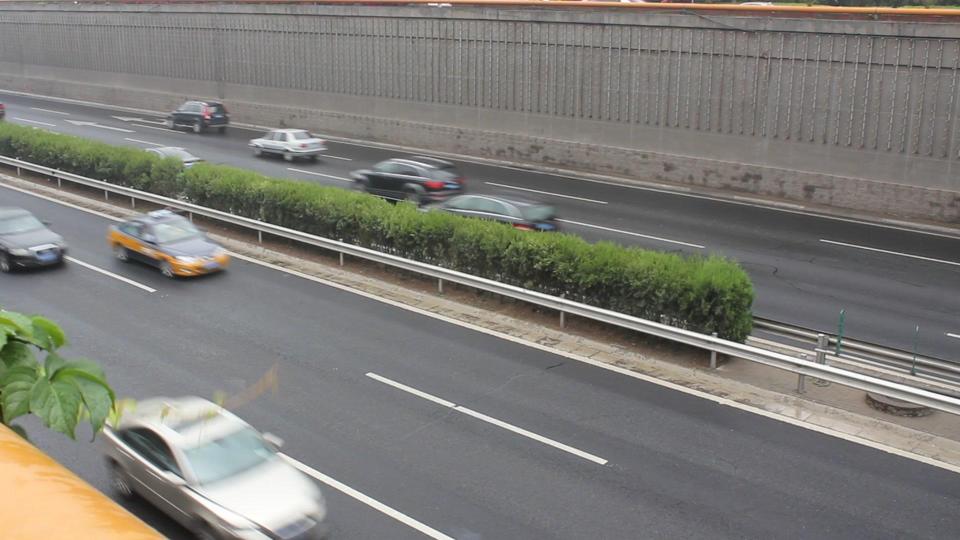

Supplement: Data S1 [file peerj-cs-09-1411-s001.zip › dataset/MVI_63553_img00603.jpg]

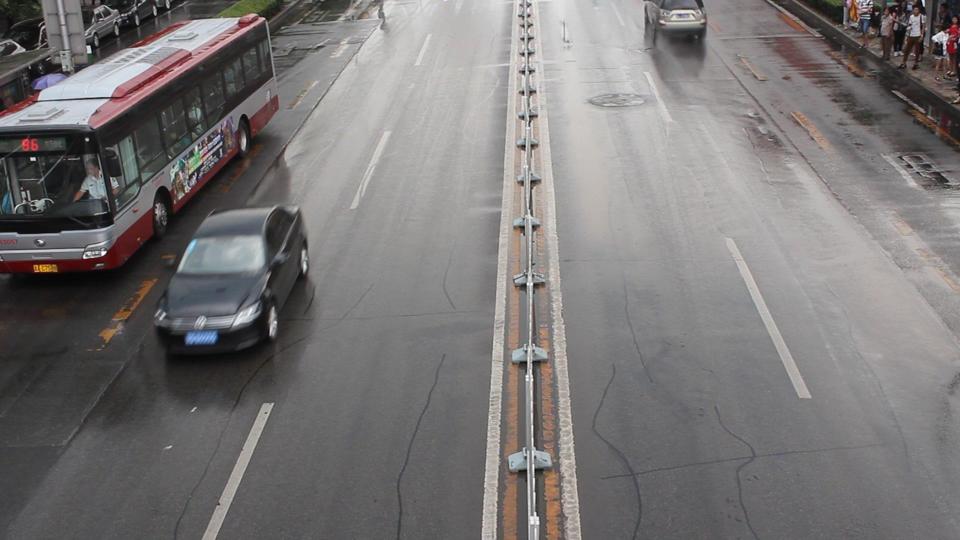

Supplement: Data S1 [file peerj-cs-09-1411-s001.zip › dataset/MVI_63525_img00172.jpg]

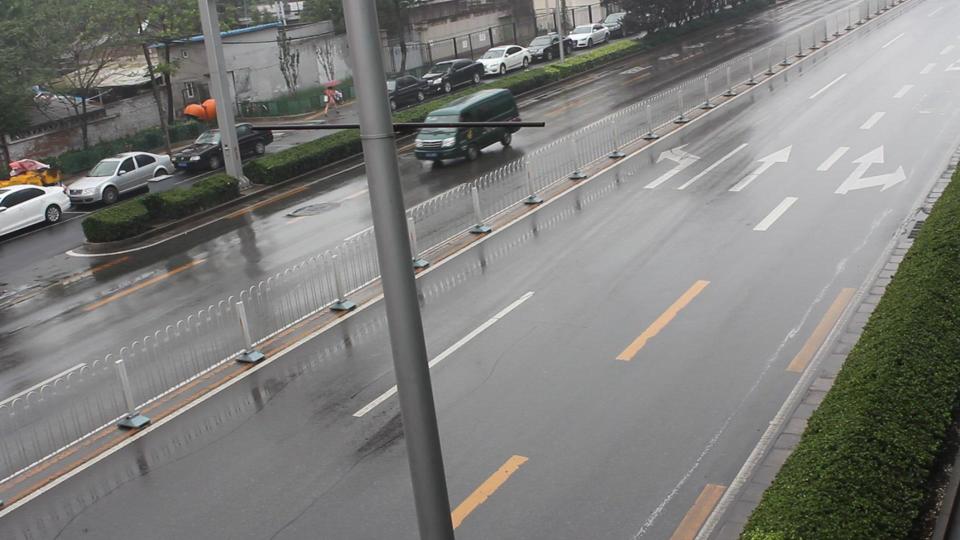

Supplement: Data S1 [file peerj-cs-09-1411-s001.zip › dataset/MVI_63544_img00433.jpg]

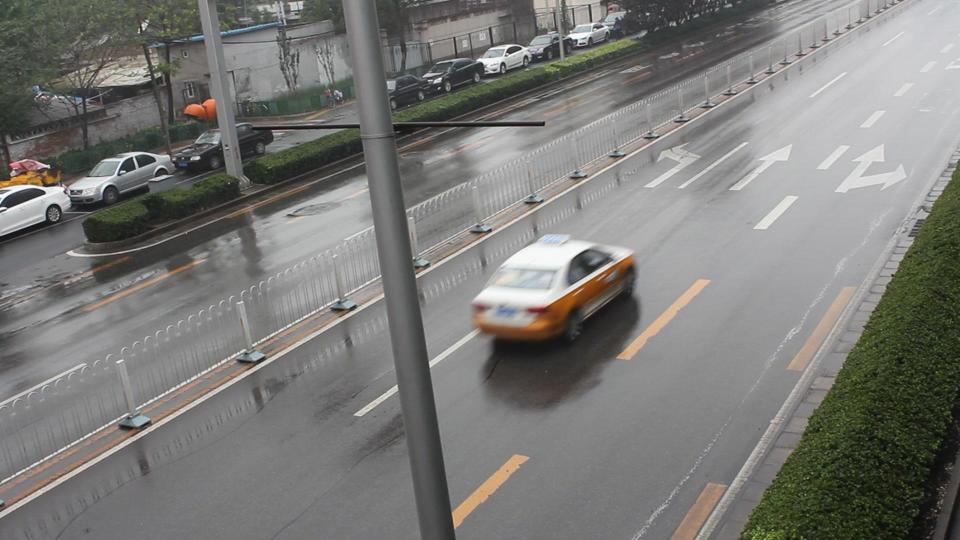

Supplement: Data S1 [file peerj-cs-09-1411-s001.zip › dataset/MVI_63544_img01117.jpg]

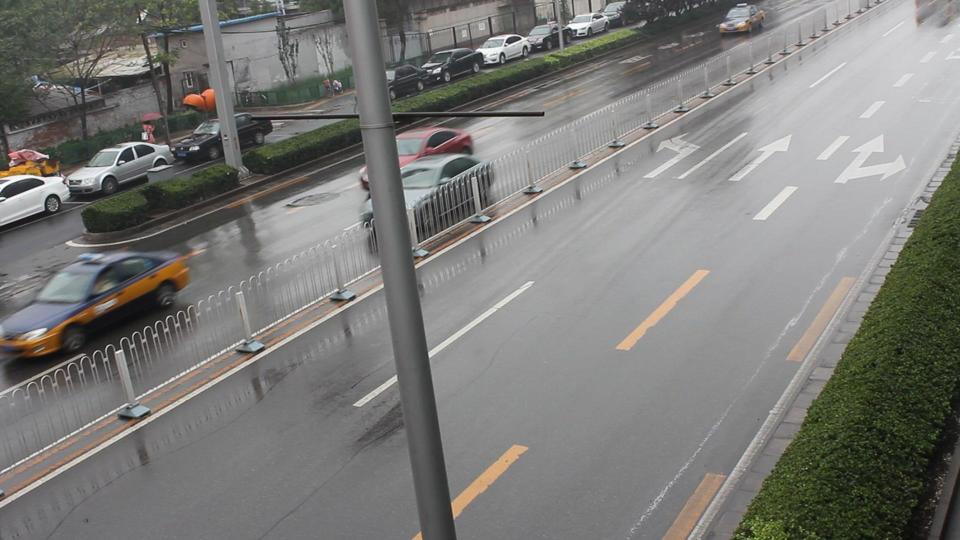

Supplement: Data S1 [file peerj-cs-09-1411-s001.zip › dataset/MVI_63544_img00209.jpg]

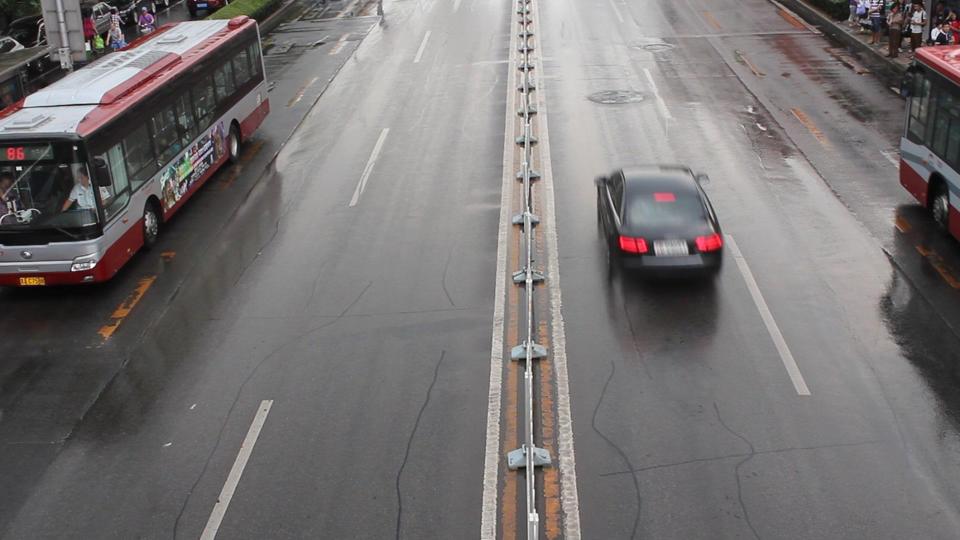

Supplement: Data S1 [file peerj-cs-09-1411-s001.zip › dataset/MVI_63525_img00774.jpg]

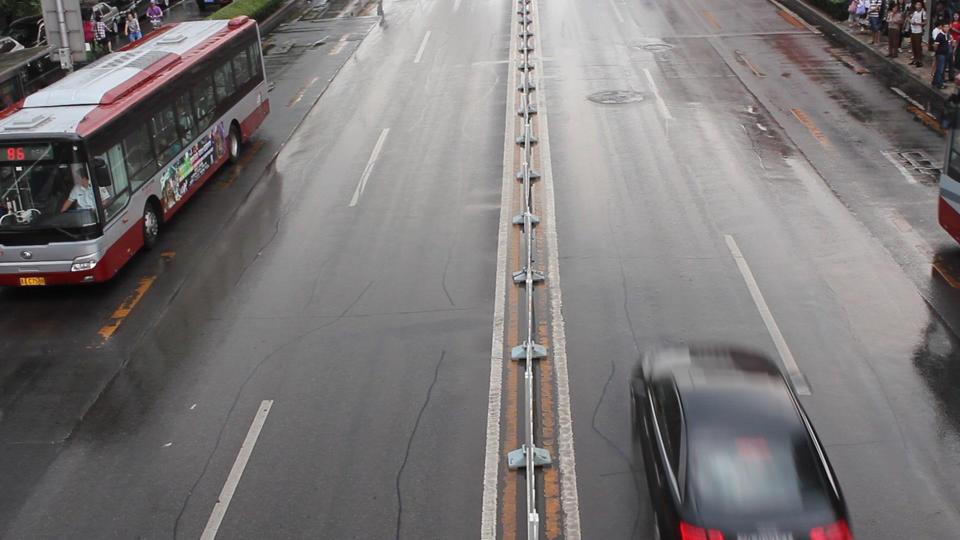

Supplement: Data S1 [file peerj-cs-09-1411-s001.zip › dataset/MVI_63525_img00760.jpg]

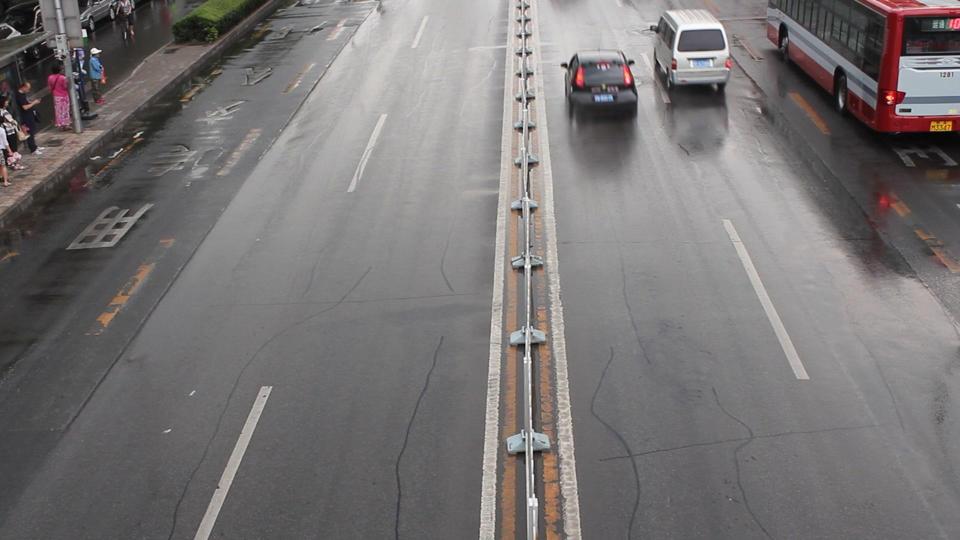

Supplement: Data S1 [file peerj-cs-09-1411-s001.zip › dataset/MVI_63525_img00984.jpg]

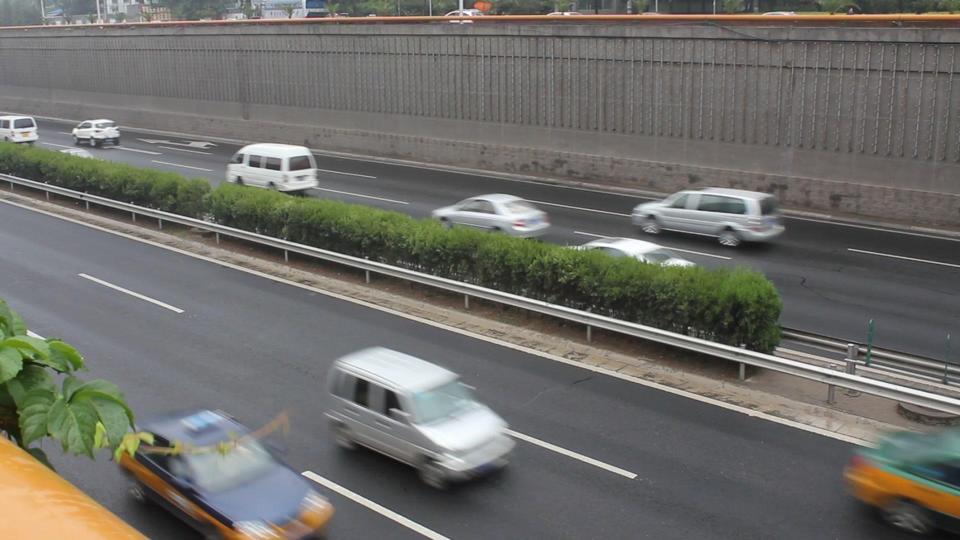

Supplement: Data S1 [file peerj-cs-09-1411-s001.zip › dataset/MVI_63552_img00638.jpg]

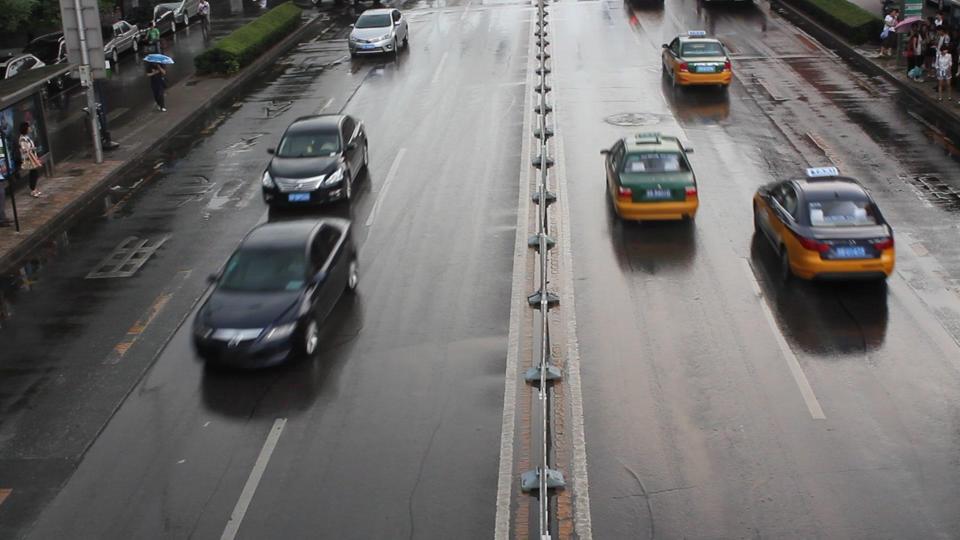

Supplement: Data S1 [file peerj-cs-09-1411-s001.zip › dataset/MVI_63521_img01891.jpg]

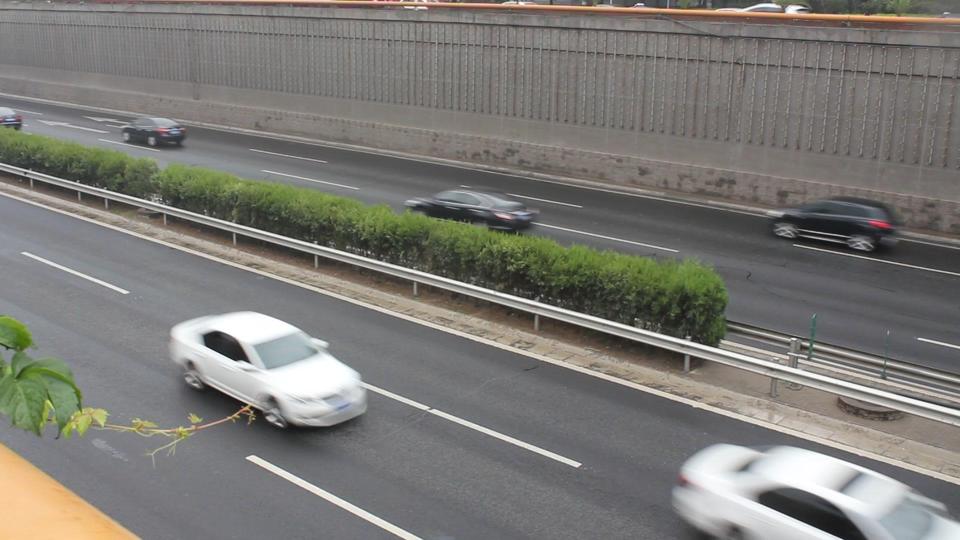

Supplement: Data S1 [file peerj-cs-09-1411-s001.zip › dataset/MVI_63554_img01044.jpg]

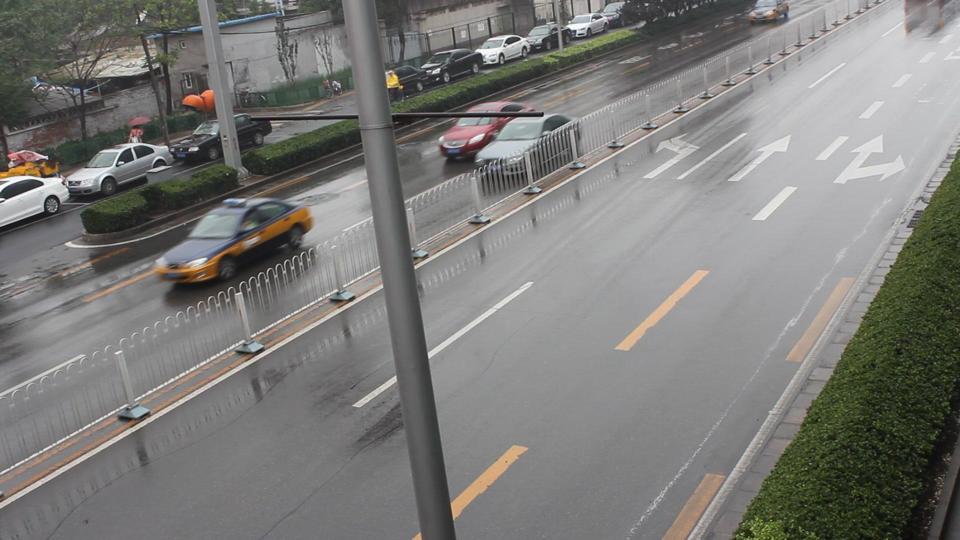

Supplement: Data S1 [file peerj-cs-09-1411-s001.zip › dataset/MVI_63544_img00195.jpg]

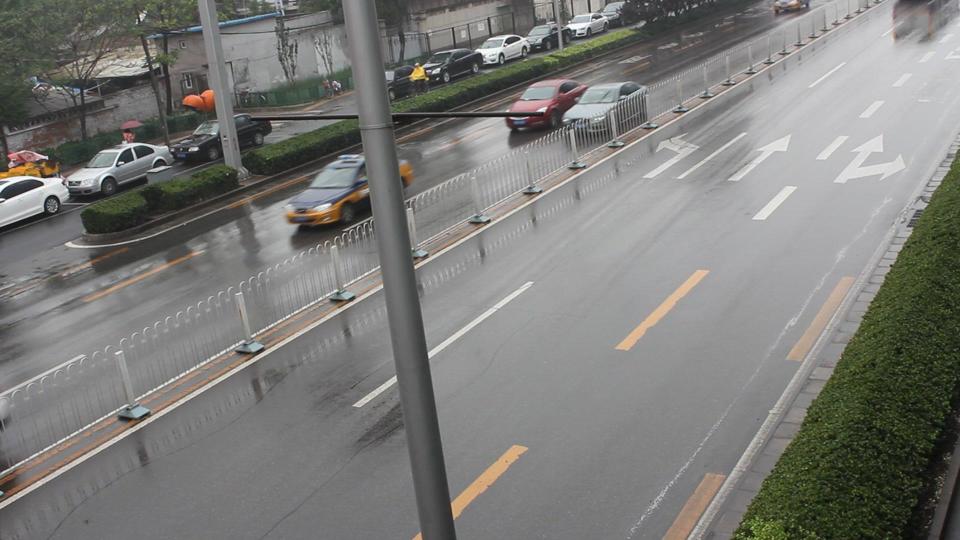

Supplement: Data S1 [file peerj-cs-09-1411-s001.zip › dataset/MVI_63544_img00181.jpg]

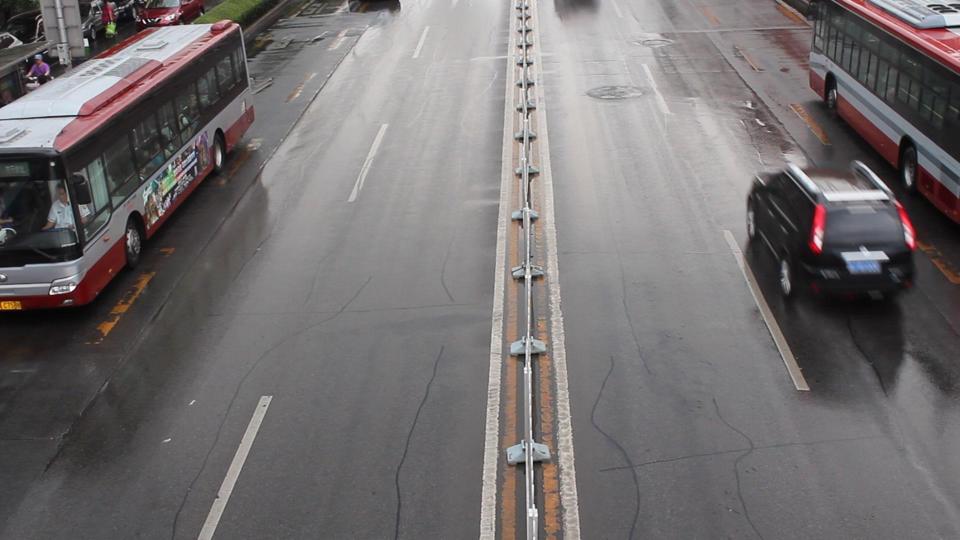

Supplement: Data S1 [file peerj-cs-09-1411-s001.zip › dataset/MVI_63525_img00830.jpg]

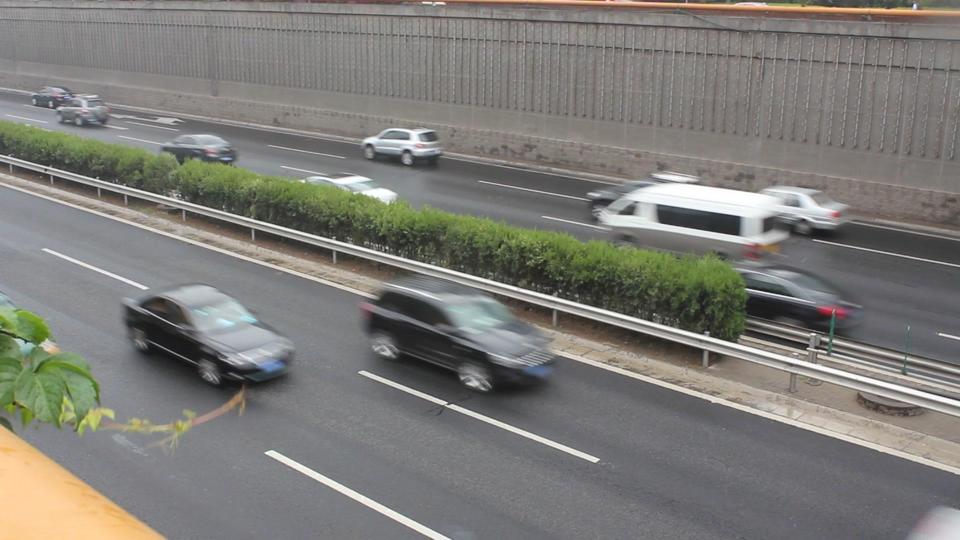

Supplement: Data S1 [file peerj-cs-09-1411-s001.zip › dataset/MVI_63554_img00576.jpg]

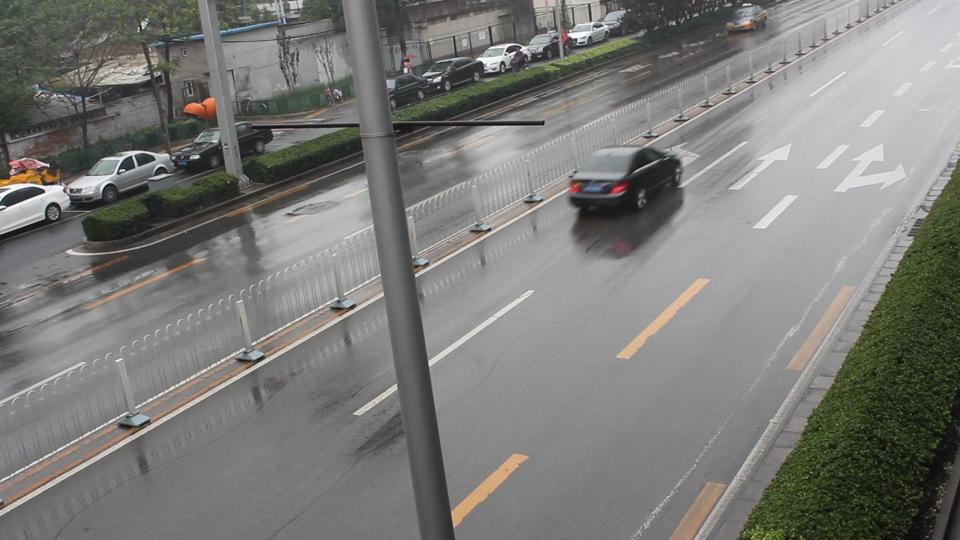

Supplement: Data S1 [file peerj-cs-09-1411-s001.zip › dataset/MVI_63544_img00587.jpg]

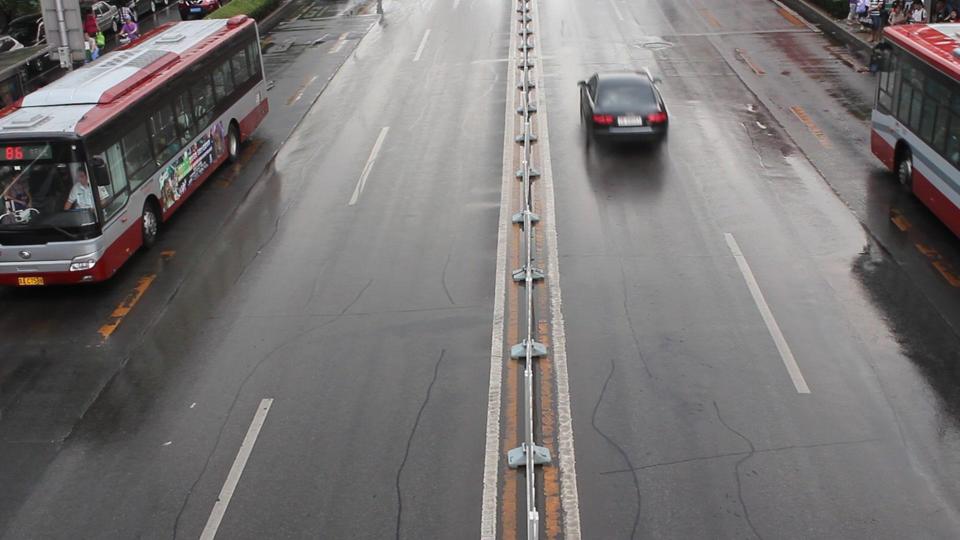

Supplement: Data S1 [file peerj-cs-09-1411-s001.zip › dataset/MVI_63525_img00788.jpg]

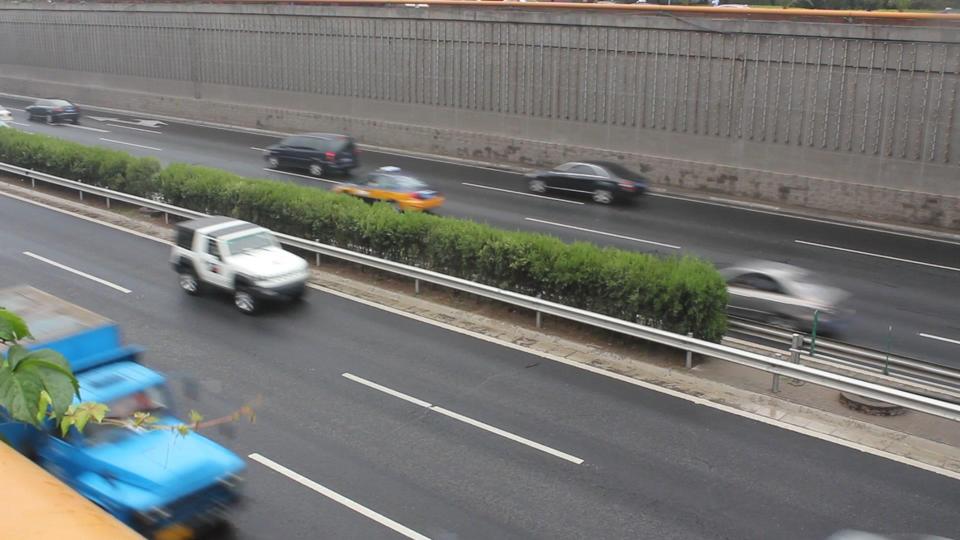

Supplement: Data S1 [file peerj-cs-09-1411-s001.zip › dataset/MVI_63554_img00774.jpg]

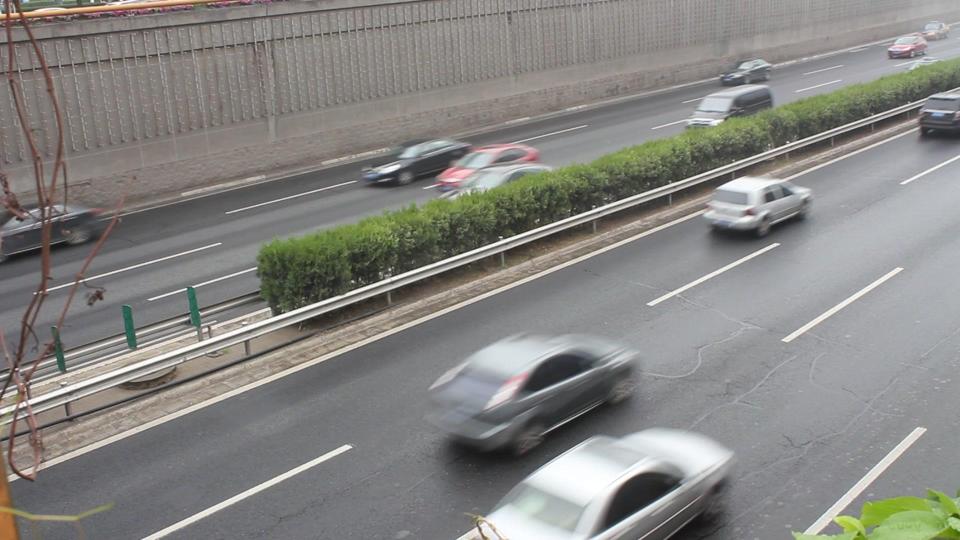

Supplement: Data S1 [file peerj-cs-09-1411-s001.zip › dataset/MVI_63561_img00811.jpg]

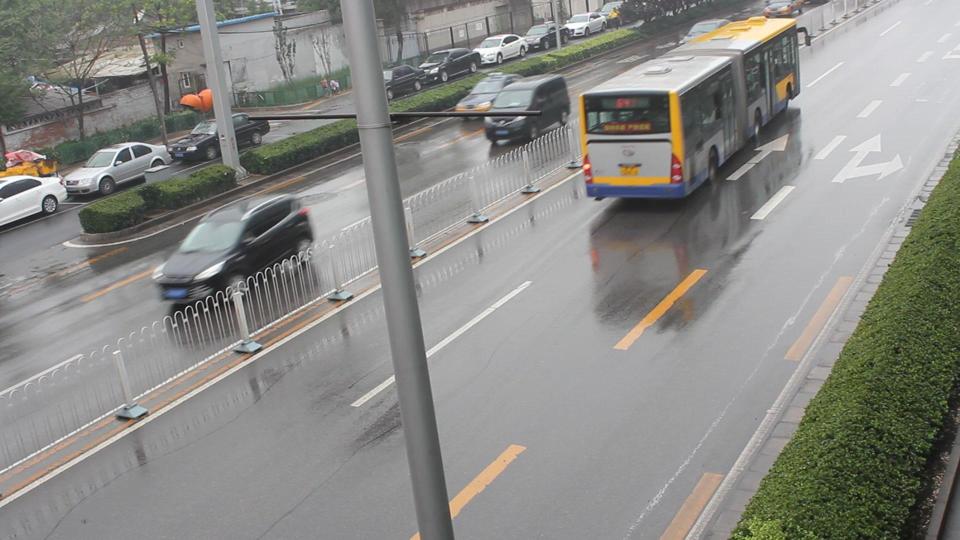

Supplement: Data S1 [file peerj-cs-09-1411-s001.zip › dataset/MVI_63544_img00034.jpg]

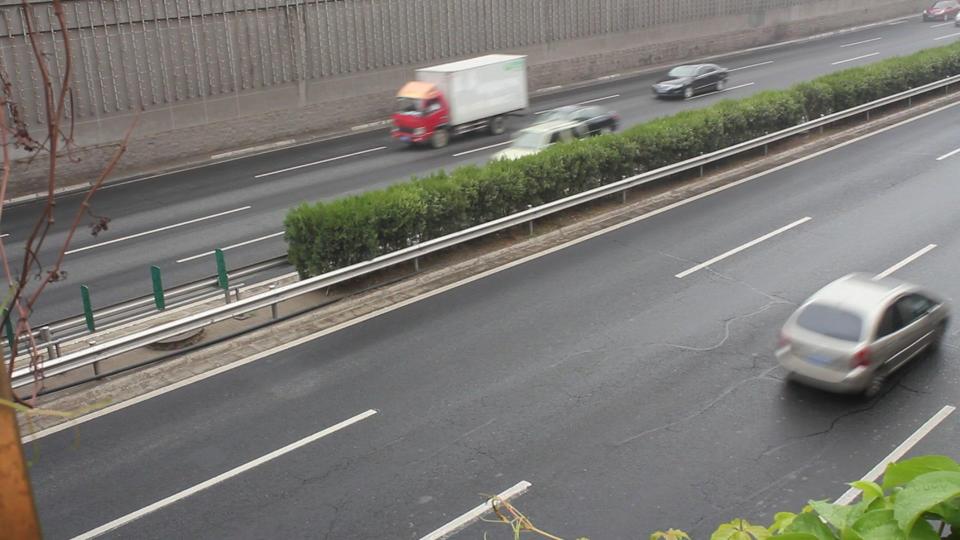

Supplement: Data S1 [file peerj-cs-09-1411-s001.zip › dataset/MVI_63563_img00980.jpg]

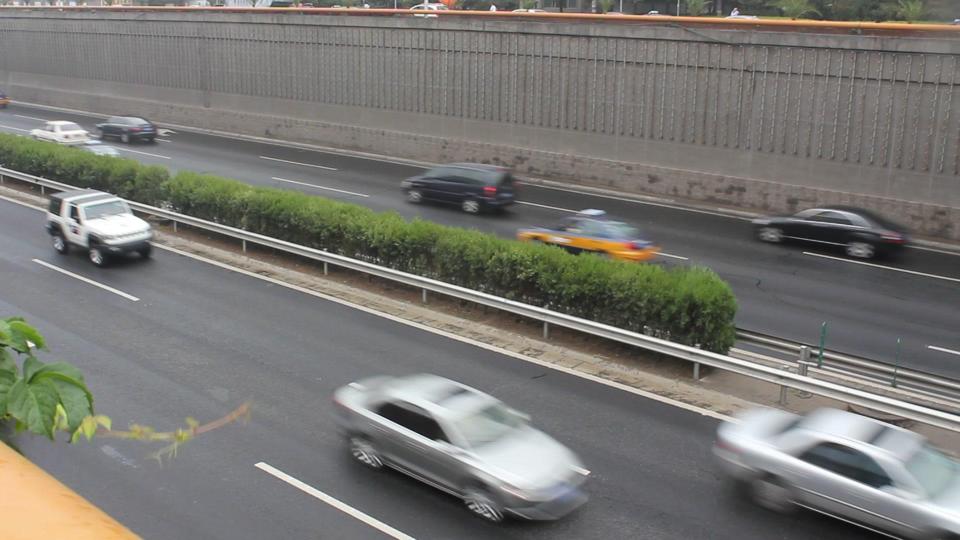

Supplement: Data S1 [file peerj-cs-09-1411-s001.zip › dataset/MVI_63554_img00761.jpg]

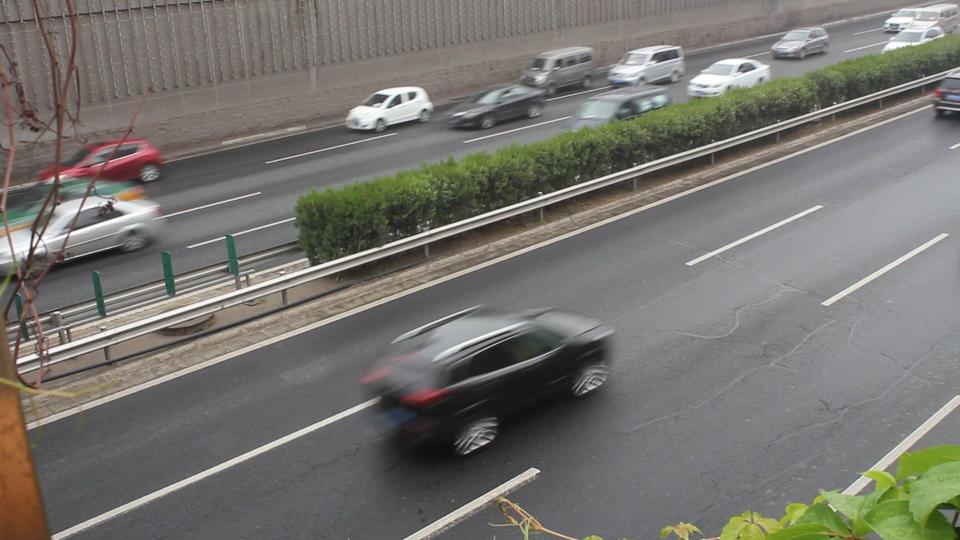

Supplement: Data S1 [file peerj-cs-09-1411-s001.zip › dataset/MVI_63563_img01136.jpg]

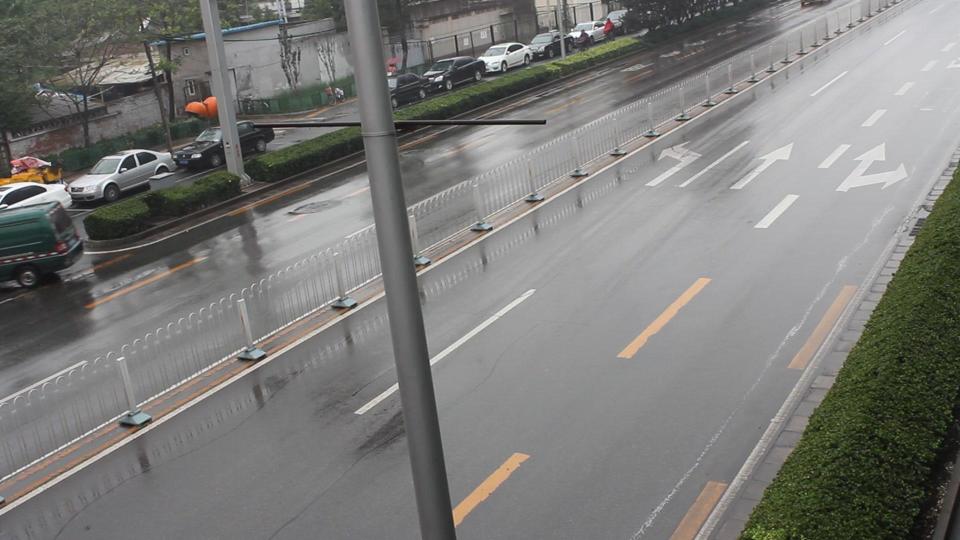

Supplement: Data S1 [file peerj-cs-09-1411-s001.zip › dataset/MVI_63544_img00545.jpg]

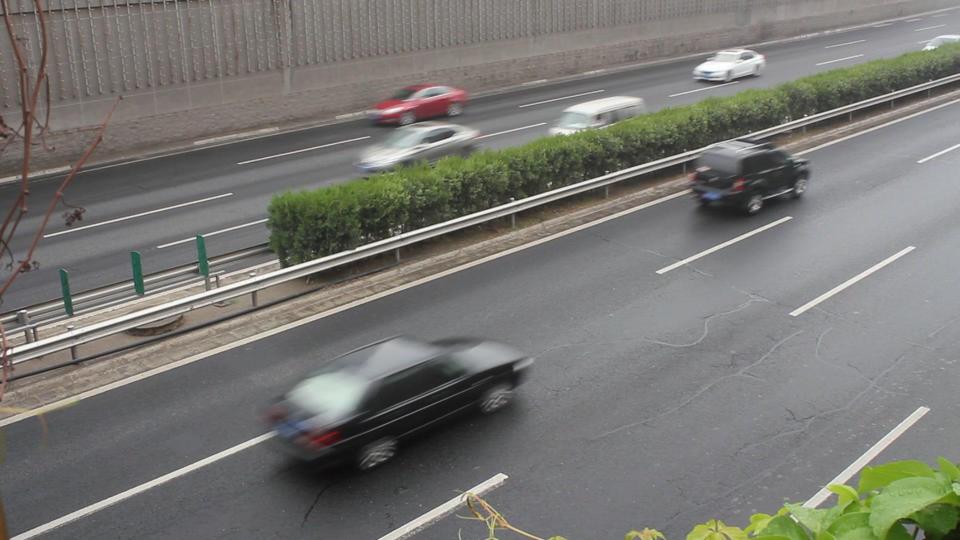

Supplement: Data S1 [file peerj-cs-09-1411-s001.zip › dataset/MVI_63562_img00603.jpg]

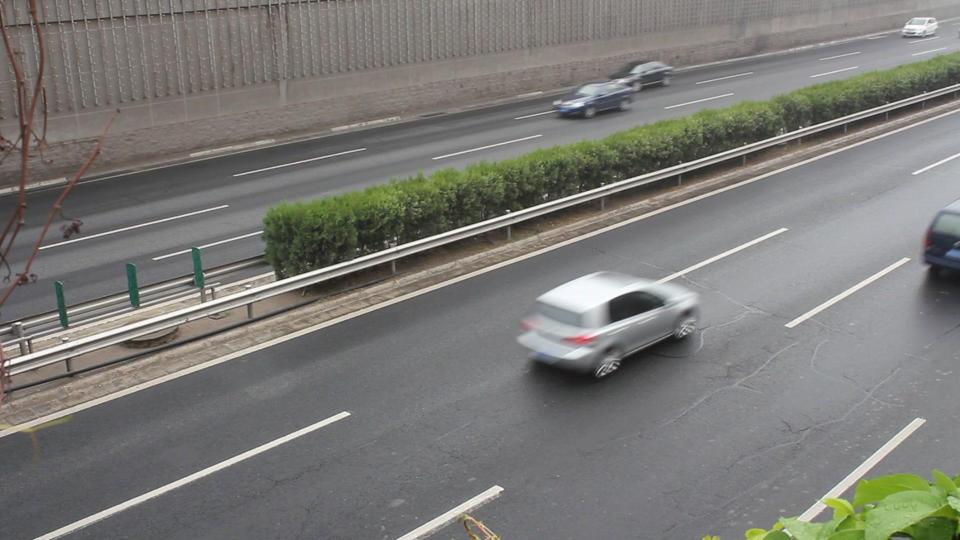

Supplement: Data S1 [file peerj-cs-09-1411-s001.zip › dataset/MVI_63562_img00824.jpg]

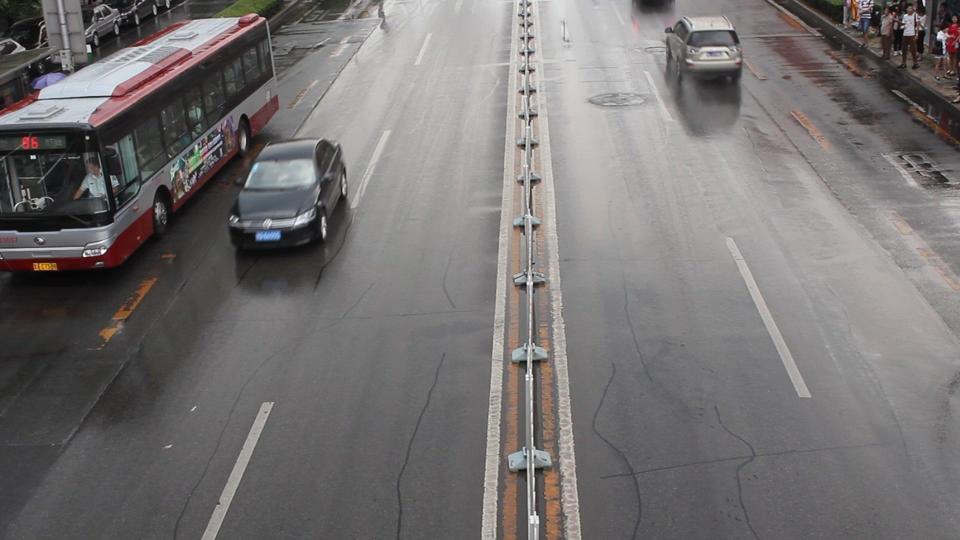

Supplement: Data S1 [file peerj-cs-09-1411-s001.zip › dataset/MVI_63525_img00158.jpg]

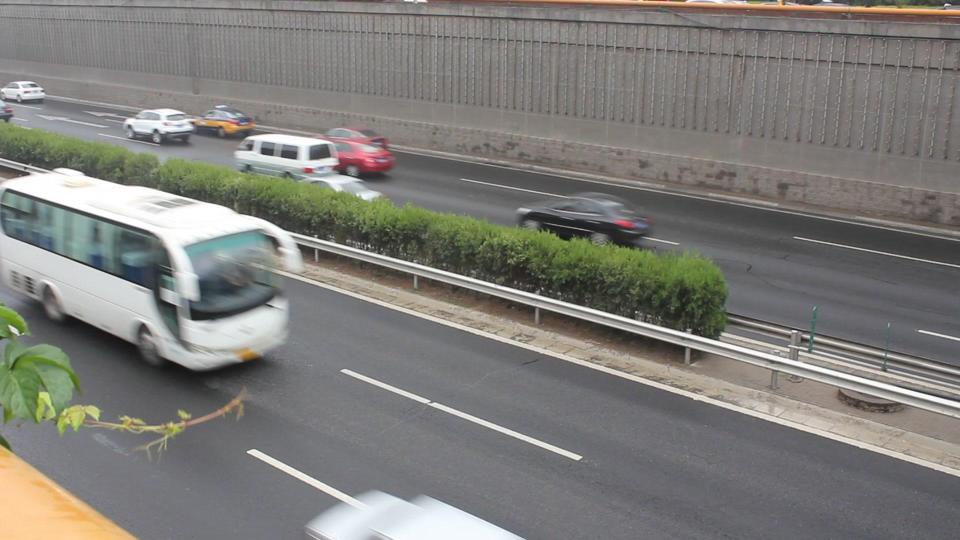

Supplement: Data S1 [file peerj-cs-09-1411-s001.zip › dataset/MVI_63553_img01292.jpg]

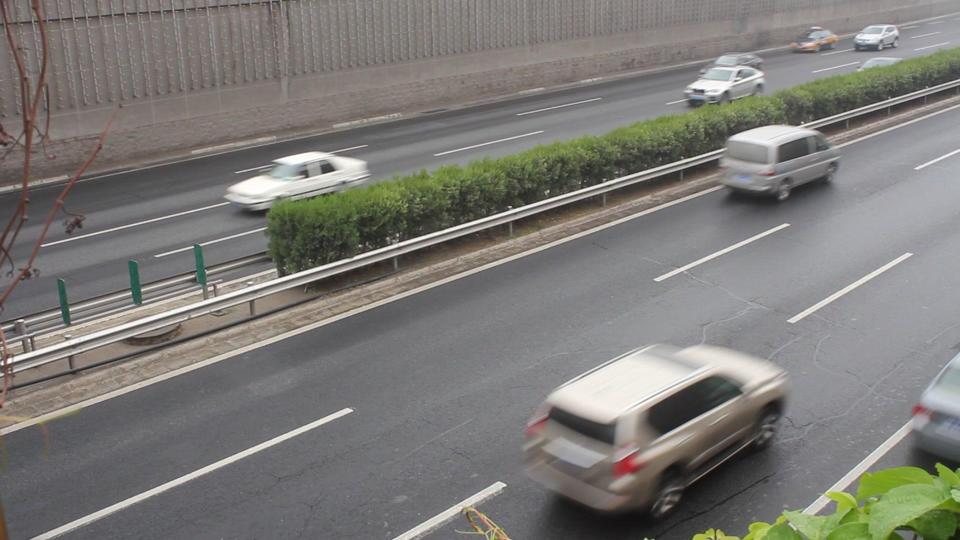

Supplement: Data S1 [file peerj-cs-09-1411-s001.zip › dataset/MVI_63562_img00993.jpg]

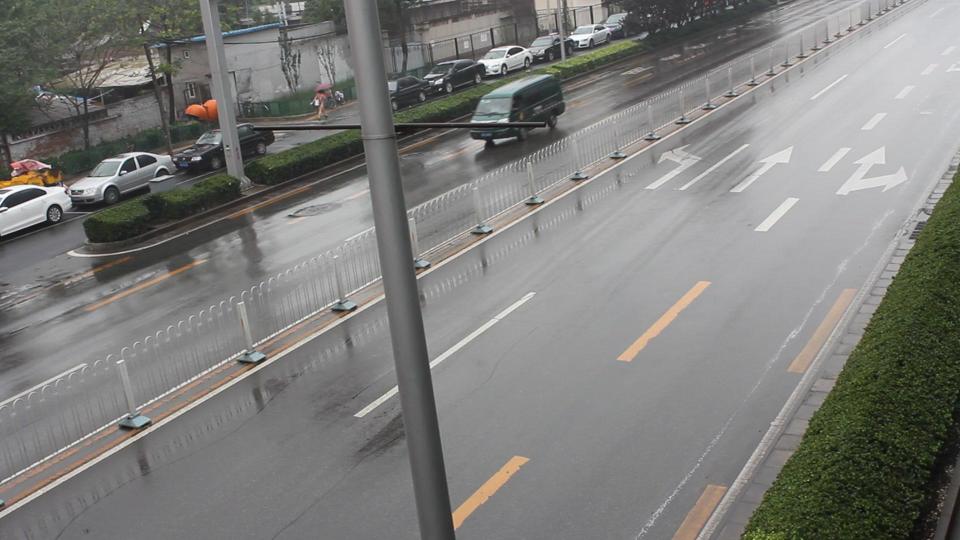

Supplement: Data S1 [file peerj-cs-09-1411-s001.zip › dataset/MVI_63544_img00419.jpg]

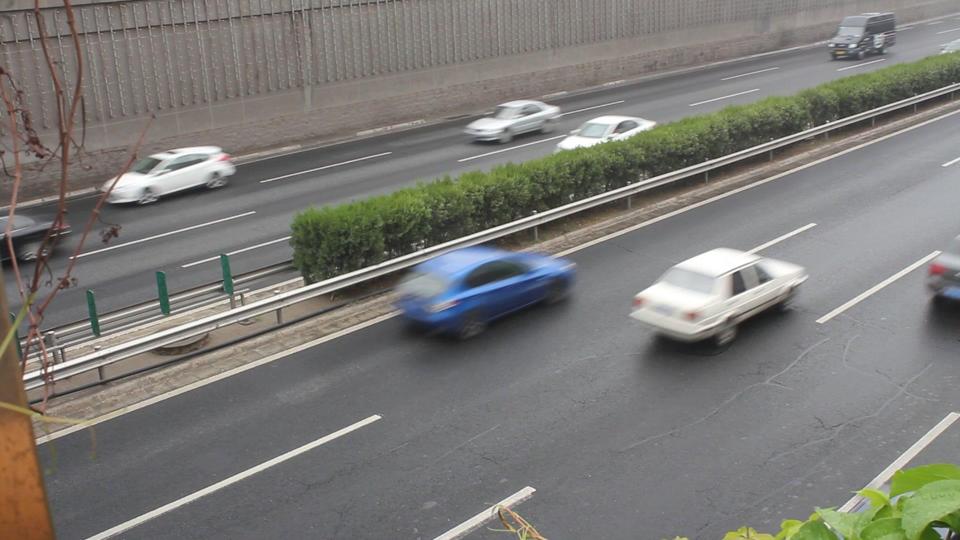

Supplement: Data S1 [file peerj-cs-09-1411-s001.zip › dataset/MVI_63563_img00406.jpg]

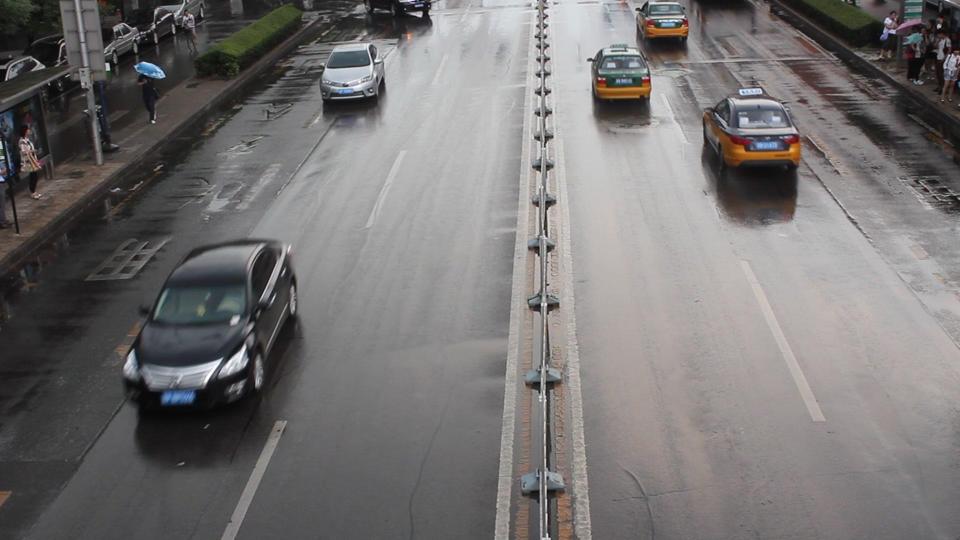

Supplement: Data S1 [file peerj-cs-09-1411-s001.zip › dataset/MVI_63521_img01919.jpg]

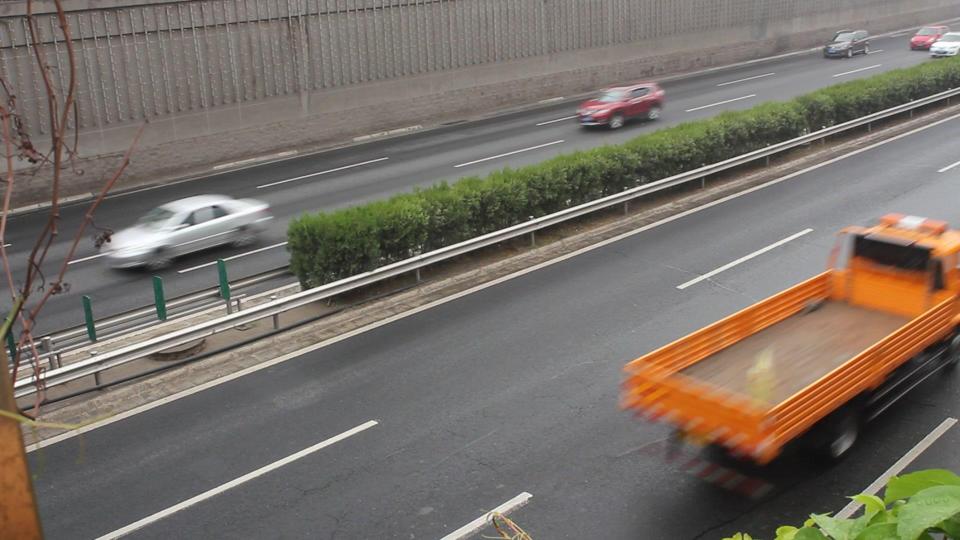

Supplement: Data S1 [file peerj-cs-09-1411-s001.zip › dataset/MVI_63563_img00850.jpg]

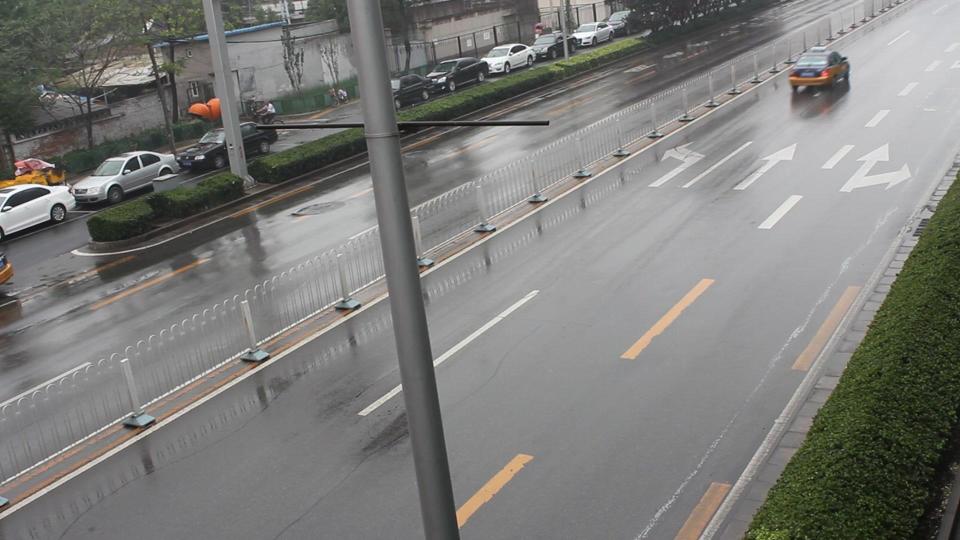

Supplement: Data S1 [file peerj-cs-09-1411-s001.zip › dataset/MVI_63544_img00867.jpg]

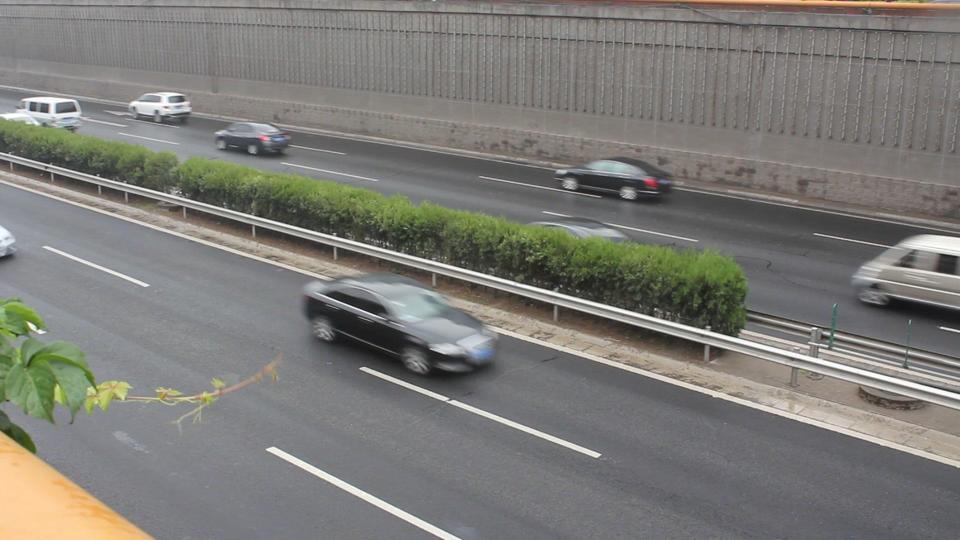

Supplement: Data S1 [file peerj-cs-09-1411-s001.zip › dataset/MVI_63554_img00316.jpg]

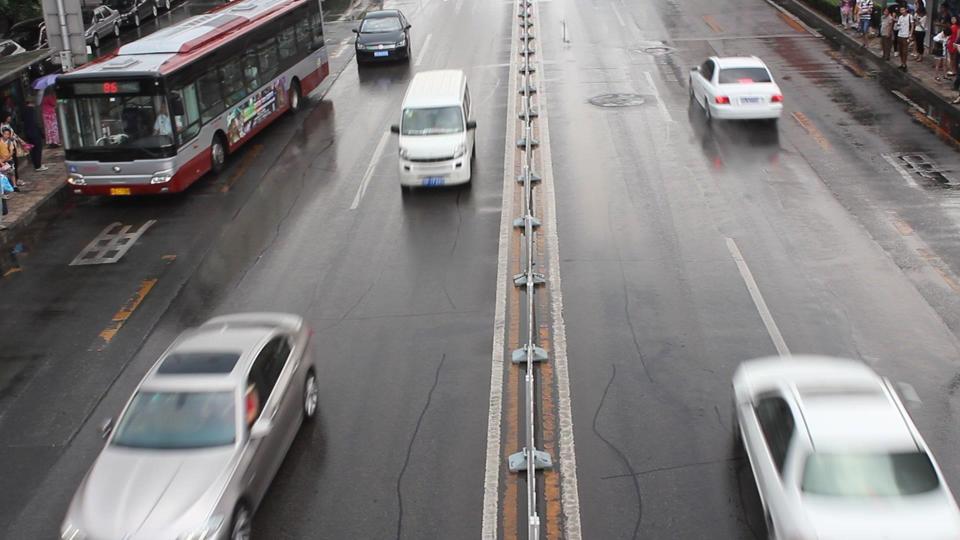

Supplement: Data S1 [file peerj-cs-09-1411-s001.zip › dataset/MVI_63525_img00088.jpg]

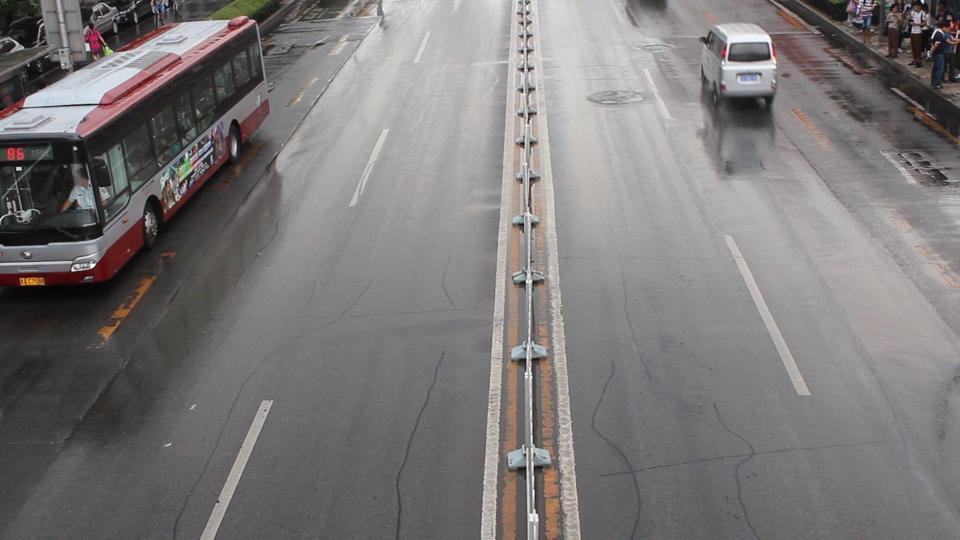

Supplement: Data S1 [file peerj-cs-09-1411-s001.zip › dataset/MVI_63525_img00704.jpg]

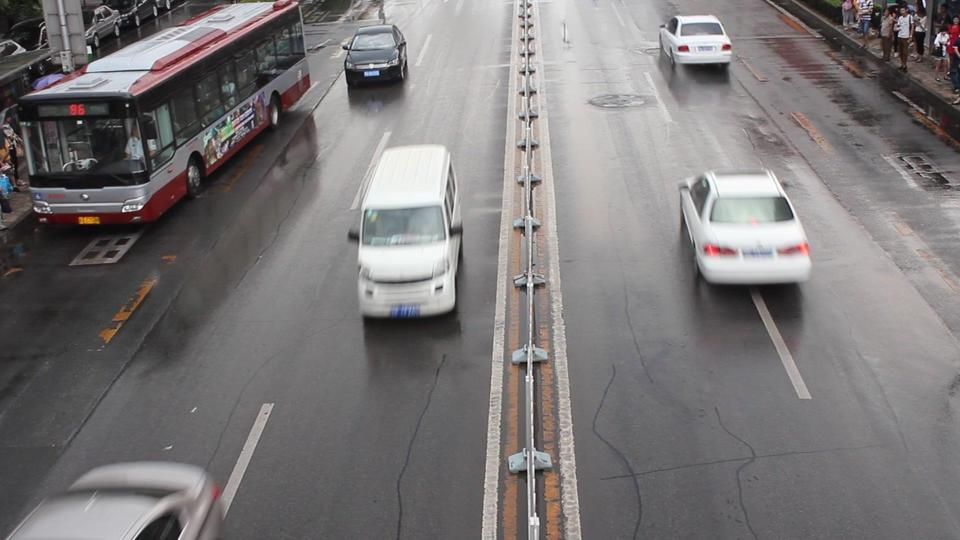

Supplement: Data S1 [file peerj-cs-09-1411-s001.zip › dataset/MVI_63525_img00102.jpg]

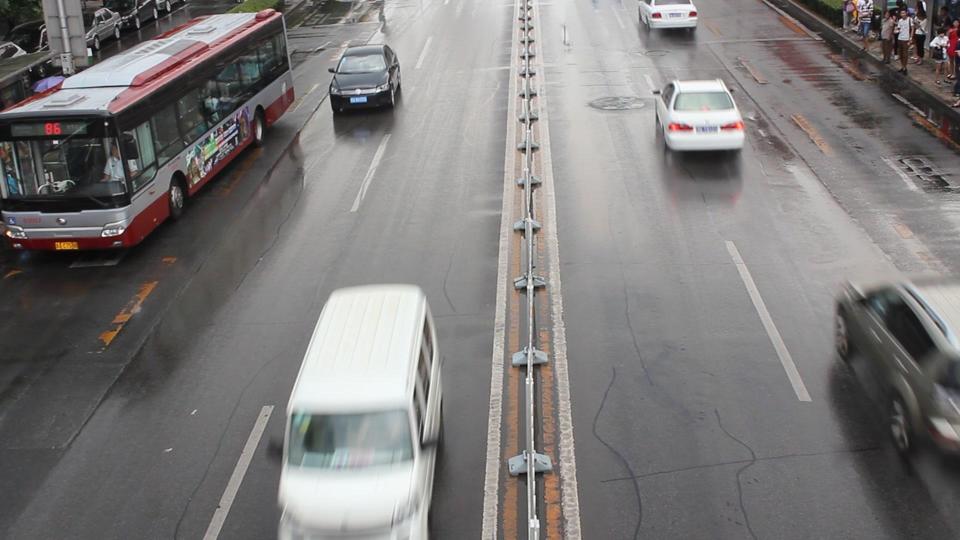

Supplement: Data S1 [file peerj-cs-09-1411-s001.zip › dataset/MVI_63525_img00116.jpg]

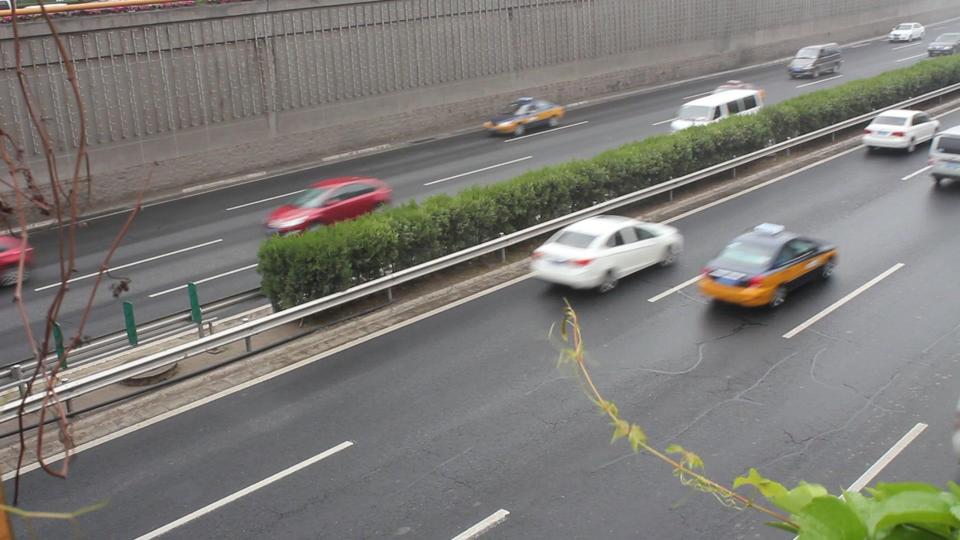

Supplement: Data S1 [file peerj-cs-09-1411-s001.zip › dataset/MVI_63561_img00902.jpg]

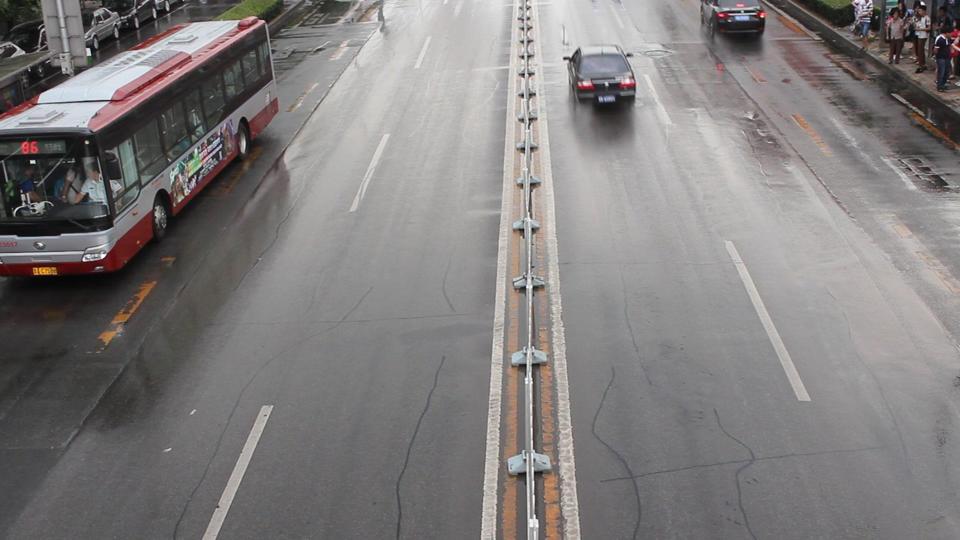

Supplement: Data S1 [file peerj-cs-09-1411-s001.zip › dataset/MVI_63525_img00466.jpg]

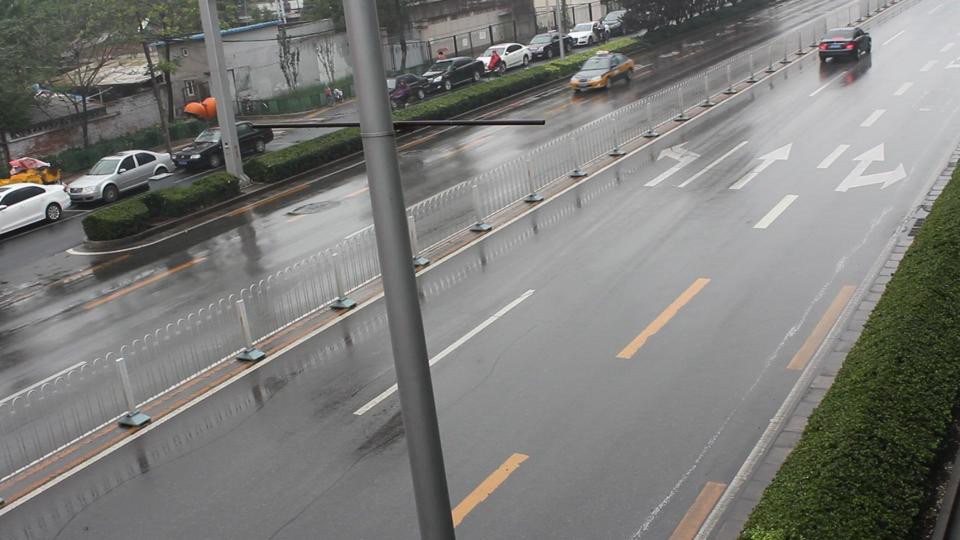

Supplement: Data S1 [file peerj-cs-09-1411-s001.zip › dataset/MVI_63544_img00643.jpg]

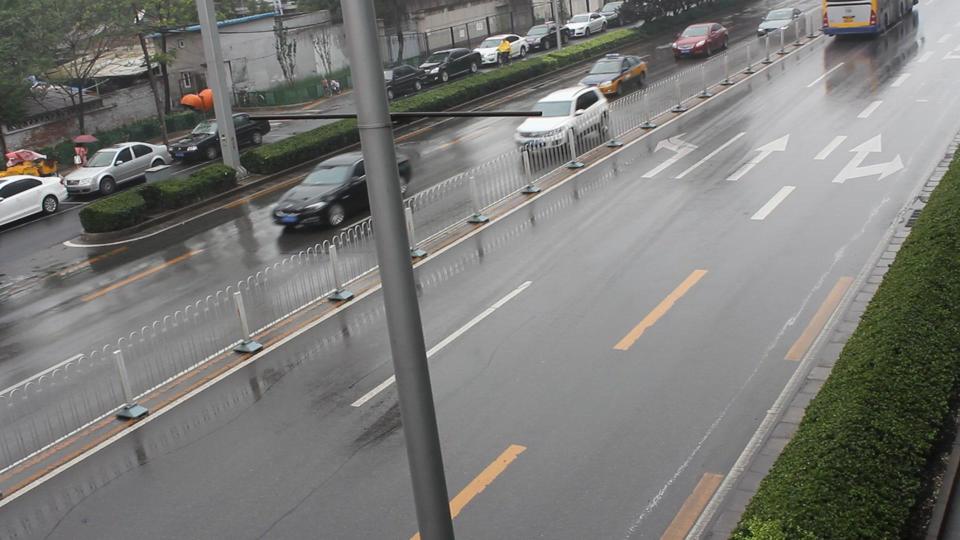

Supplement: Data S1 [file peerj-cs-09-1411-s001.zip › dataset/MVI_63544_img00125.jpg]

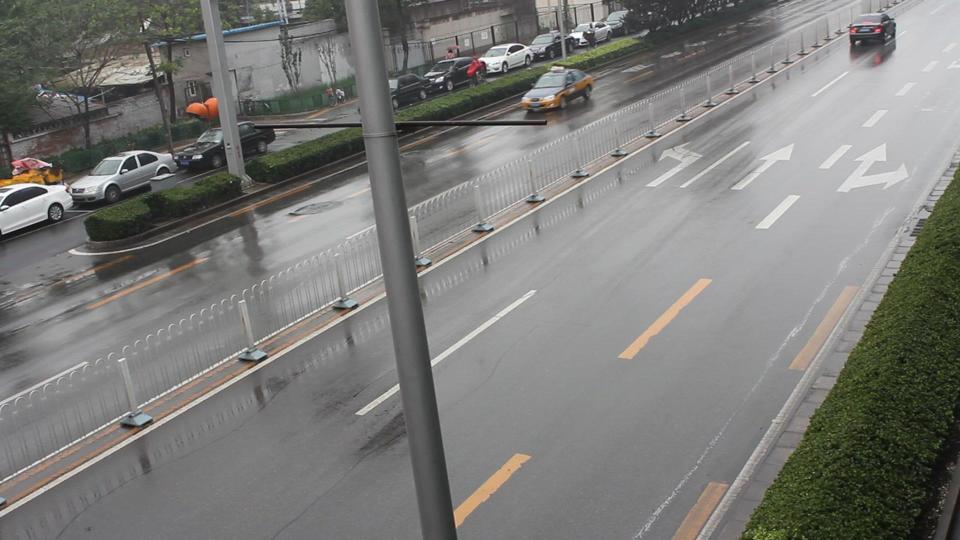

Supplement: Data S1 [file peerj-cs-09-1411-s001.zip › dataset/MVI_63544_img00657.jpg]

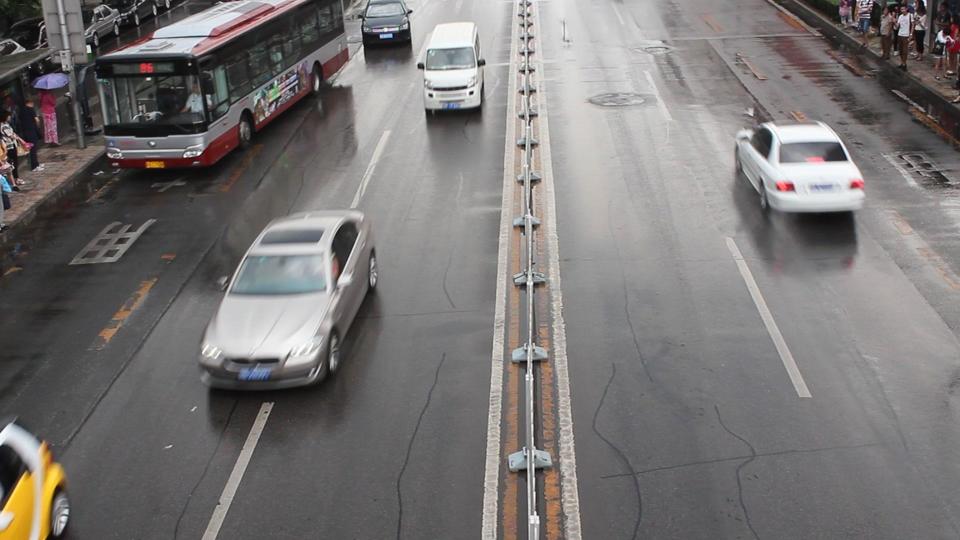

Supplement: Data S1 [file peerj-cs-09-1411-s001.zip › dataset/MVI_63525_img00074.jpg]

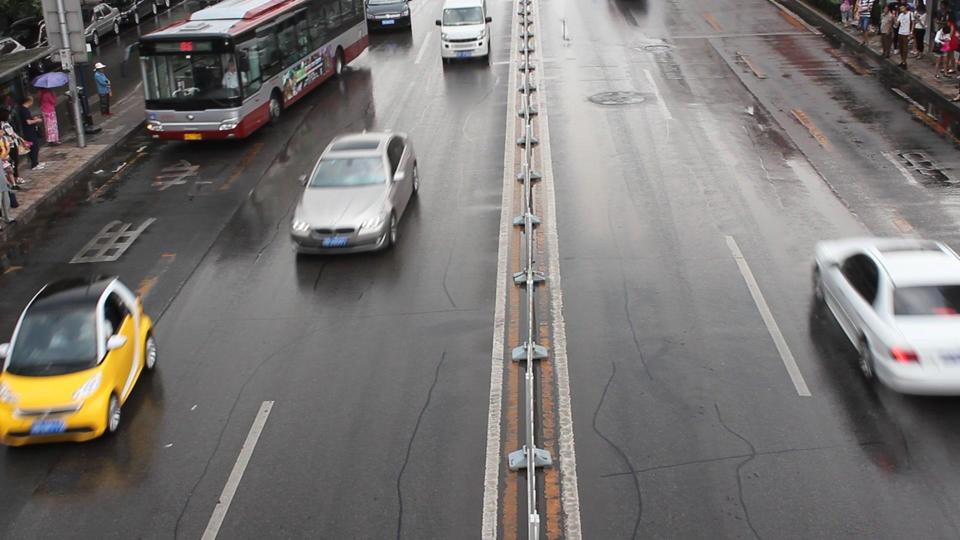

Supplement: Data S1 [file peerj-cs-09-1411-s001.zip › dataset/MVI_63525_img00060.jpg]

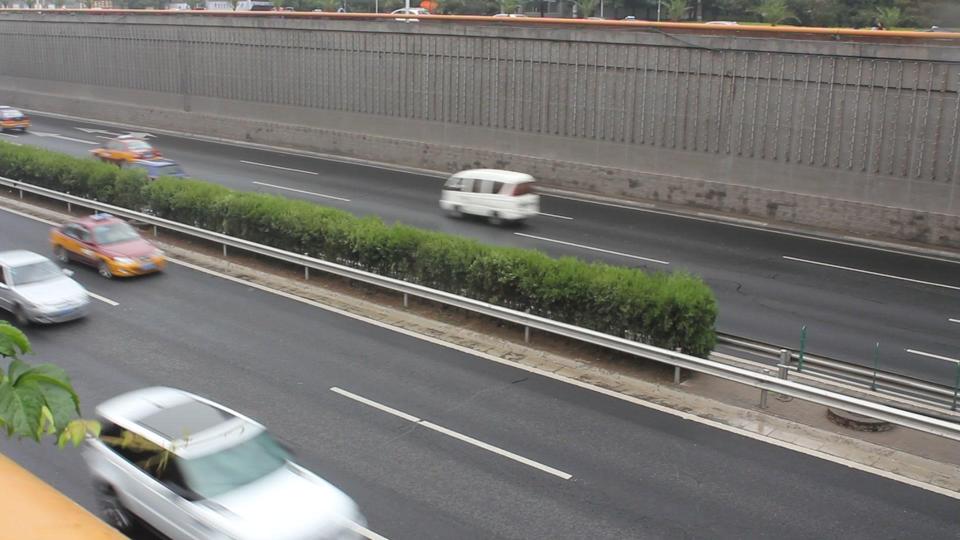

Supplement: Data S1 [file peerj-cs-09-1411-s001.zip › dataset/MVI_63554_img01355.jpg]

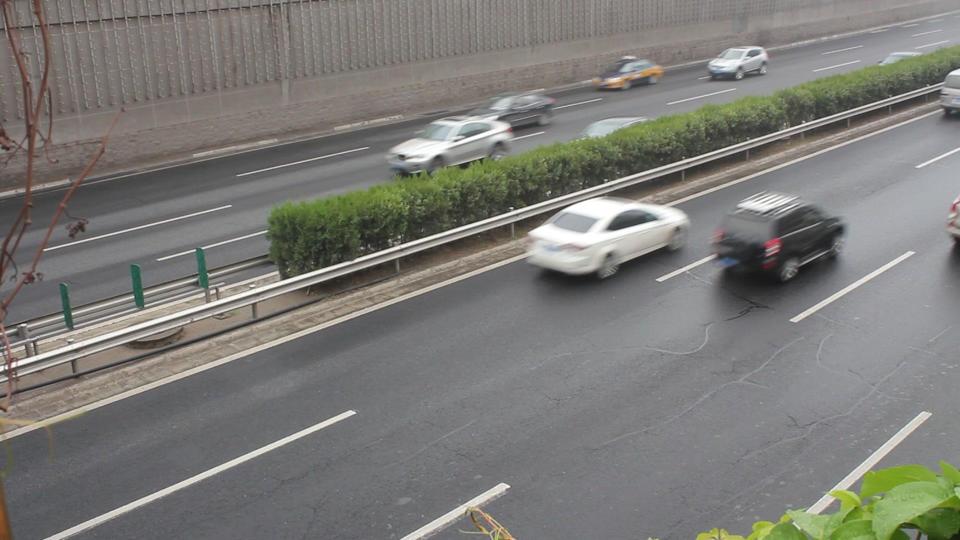

Supplement: Data S1 [file peerj-cs-09-1411-s001.zip › dataset/MVI_63562_img01021.jpg]

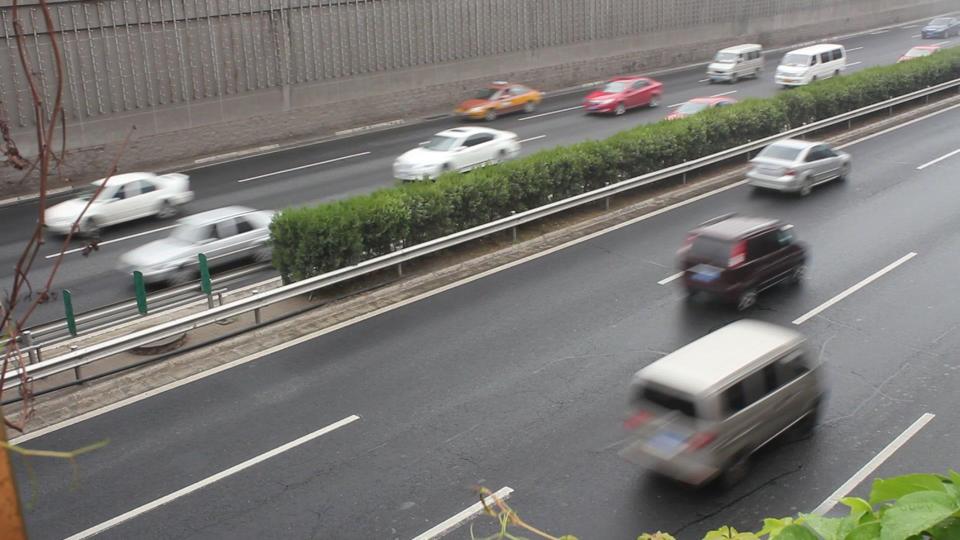

Supplement: Data S1 [file peerj-cs-09-1411-s001.zip › dataset/MVI_63562_img00317.jpg]

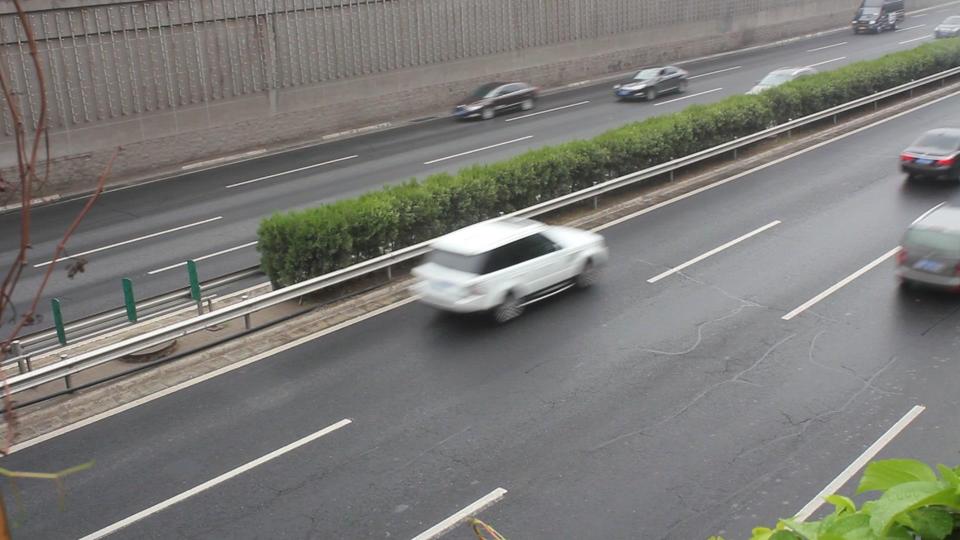

Supplement: Data S1 [file peerj-cs-09-1411-s001.zip › dataset/MVI_63561_img01160.jpg]

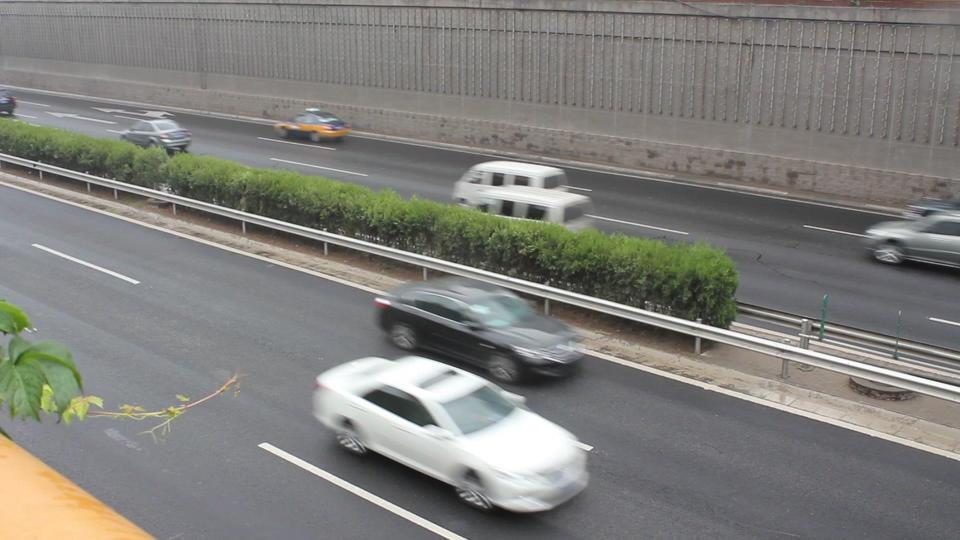

Supplement: Data S1 [file peerj-cs-09-1411-s001.zip › dataset/MVI_63554_img00133 - σë»μ£1⁄4.jpg]

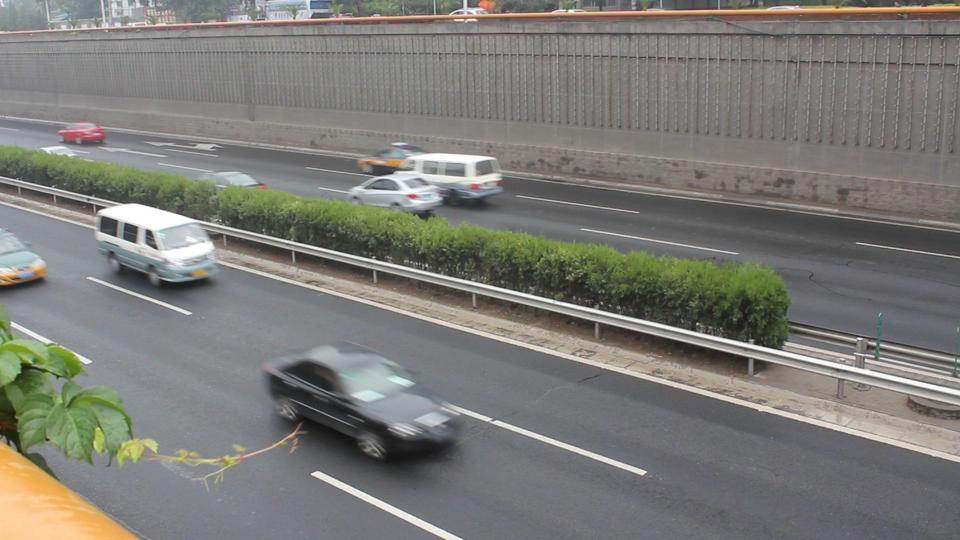

Supplement: Data S1 [file peerj-cs-09-1411-s001.zip › dataset/MVI_63552_img00529.jpg]

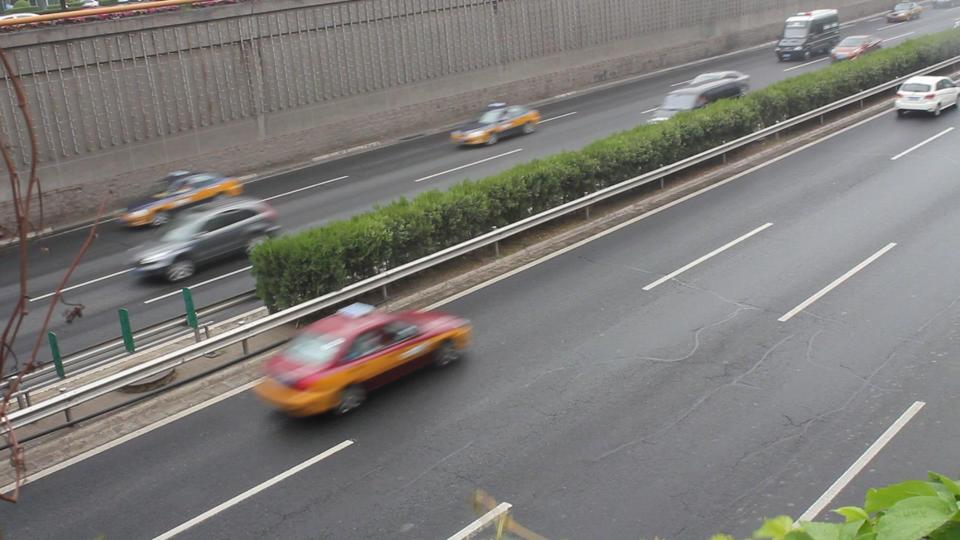

Supplement: Data S1 [file peerj-cs-09-1411-s001.zip › dataset/MVI_63561_img00406 - σë»μ£1⁄4.jpg]

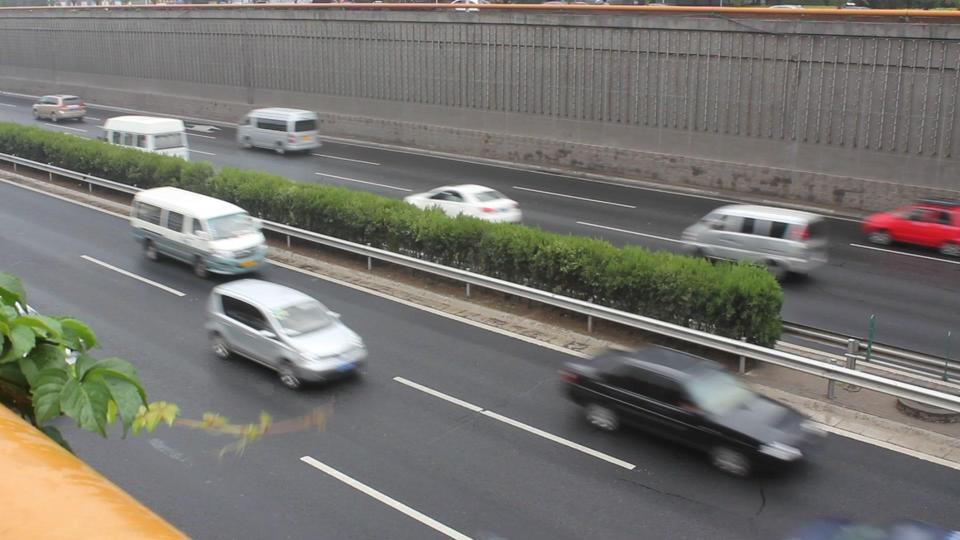

Supplement: Data S1 [file peerj-cs-09-1411-s001.zip › dataset/MVI_63553_img00021 - σë»μ£1⁄4.jpg]

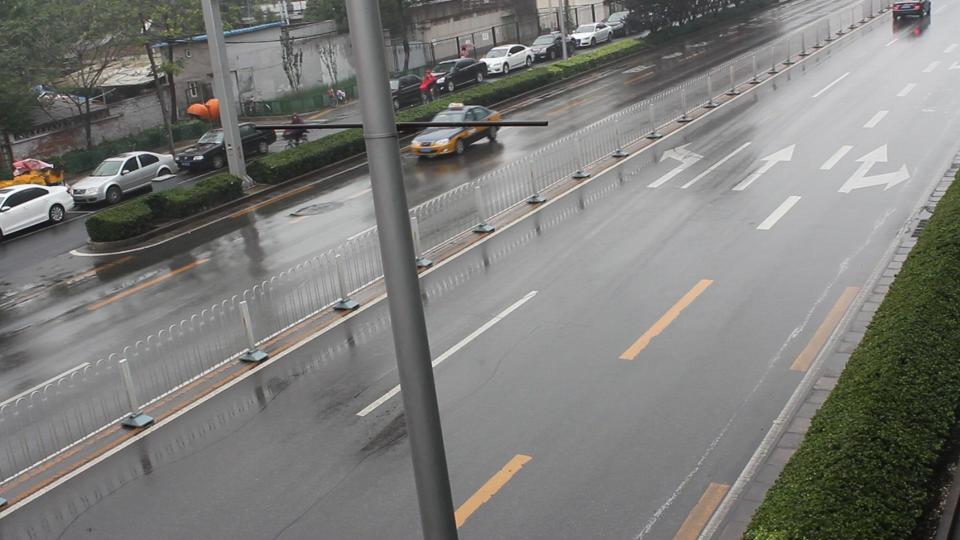

Supplement: Data S1 [file peerj-cs-09-1411-s001.zip › dataset/MVI_63544_img00685.jpg]

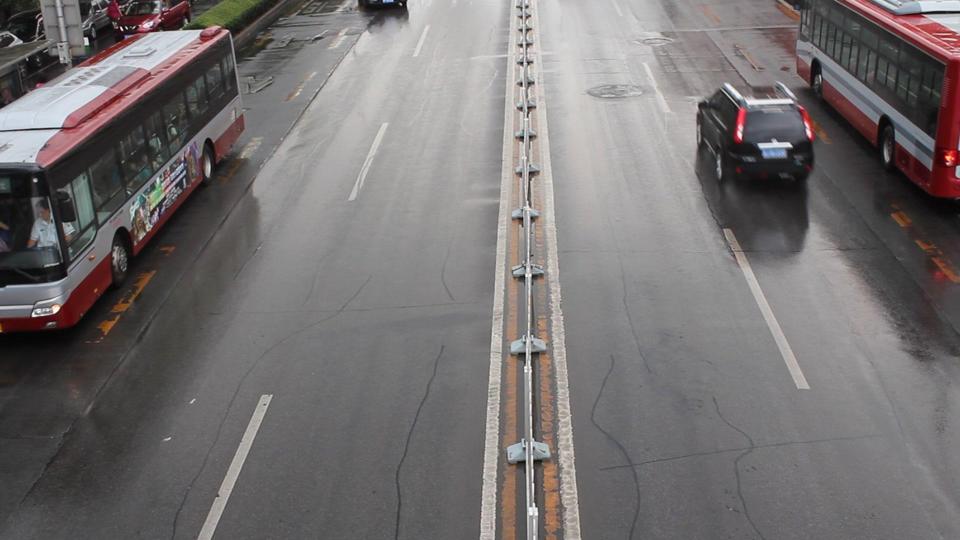

Supplement: Data S1 [file peerj-cs-09-1411-s001.zip › dataset/MVI_63525_img00844.jpg]

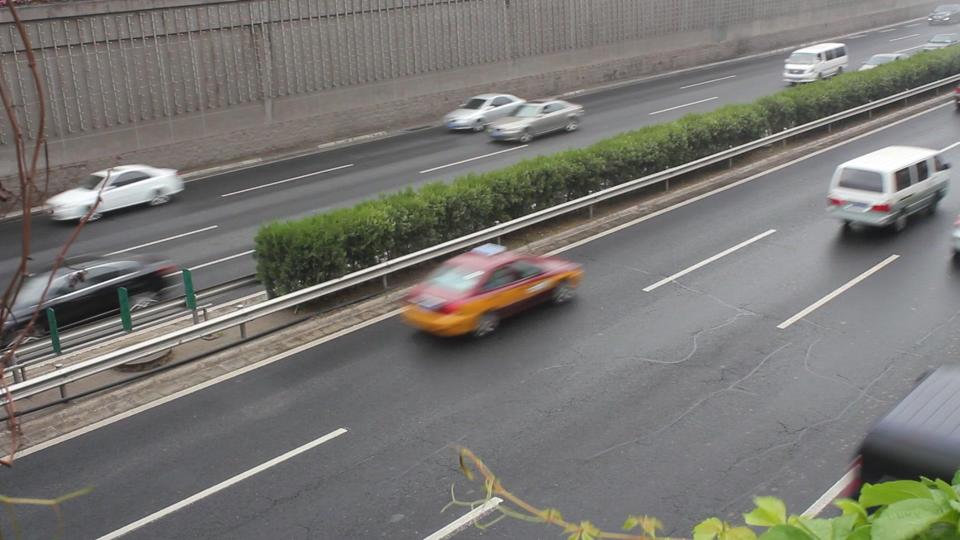

Supplement: Data S1 [file peerj-cs-09-1411-s001.zip › dataset/MVI_63561_img01004.jpg]

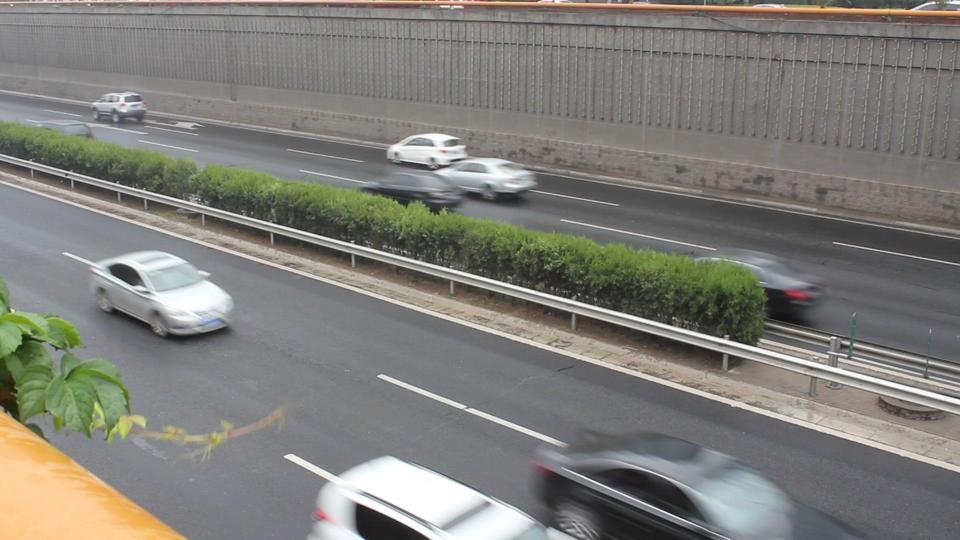

Supplement: Data S1 [file peerj-cs-09-1411-s001.zip › dataset/MVI_63553_img00264.jpg]

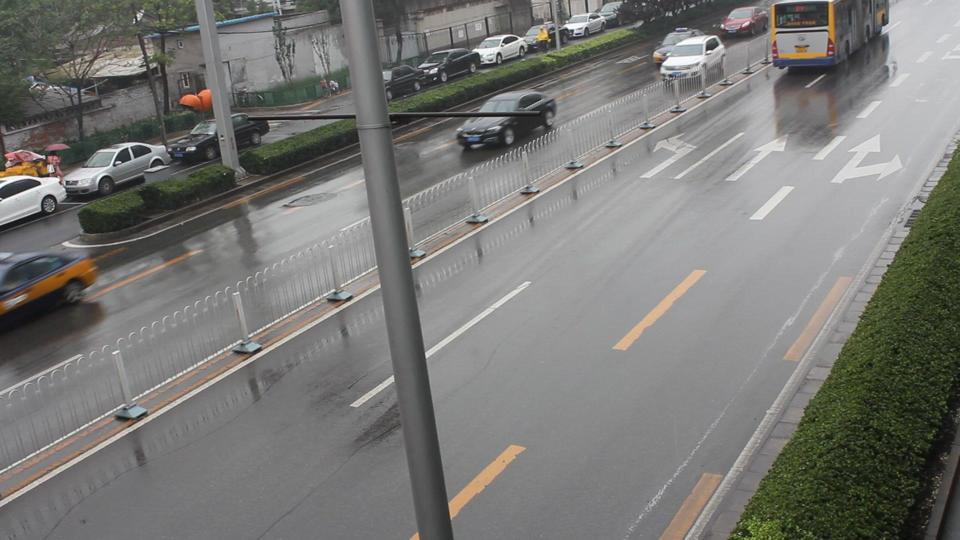

Supplement: Data S1 [file peerj-cs-09-1411-s001.zip › dataset/MVI_63544_img00097.jpg]

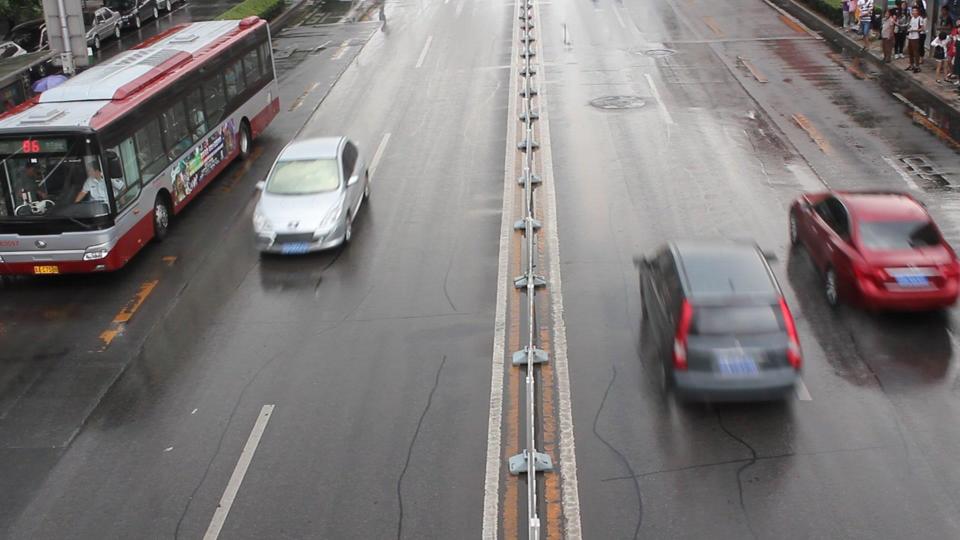

Supplement: Data S1 [file peerj-cs-09-1411-s001.zip › dataset/MVI_63525_img00298.jpg]

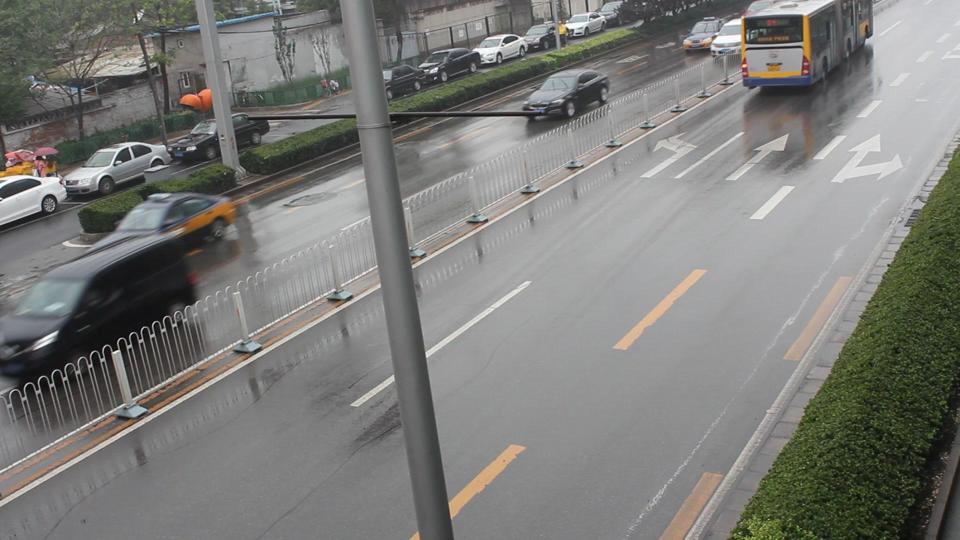

Supplement: Data S1 [file peerj-cs-09-1411-s001.zip › dataset/MVI_63544_img00083.jpg]

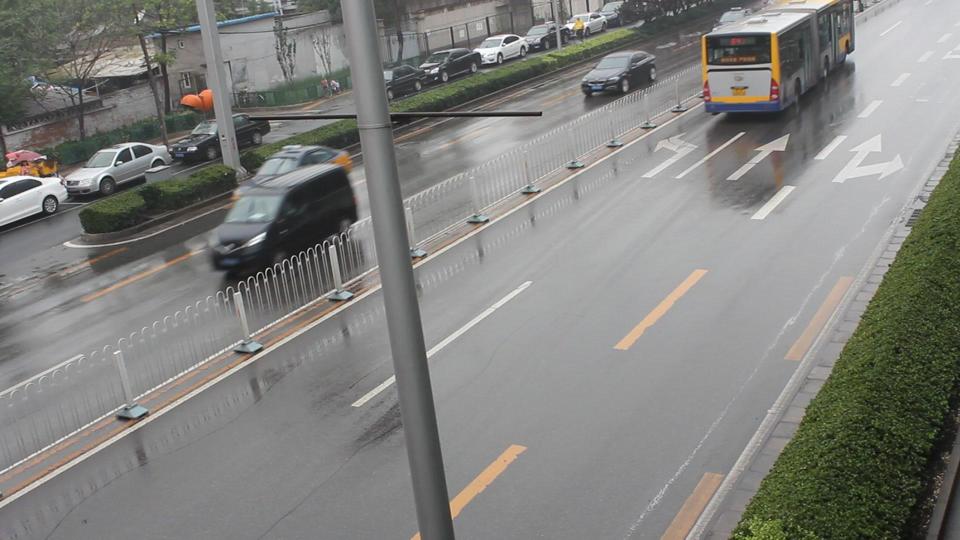

Supplement: Data S1 [file peerj-cs-09-1411-s001.zip › dataset/MVI_63544_img00068.jpg]

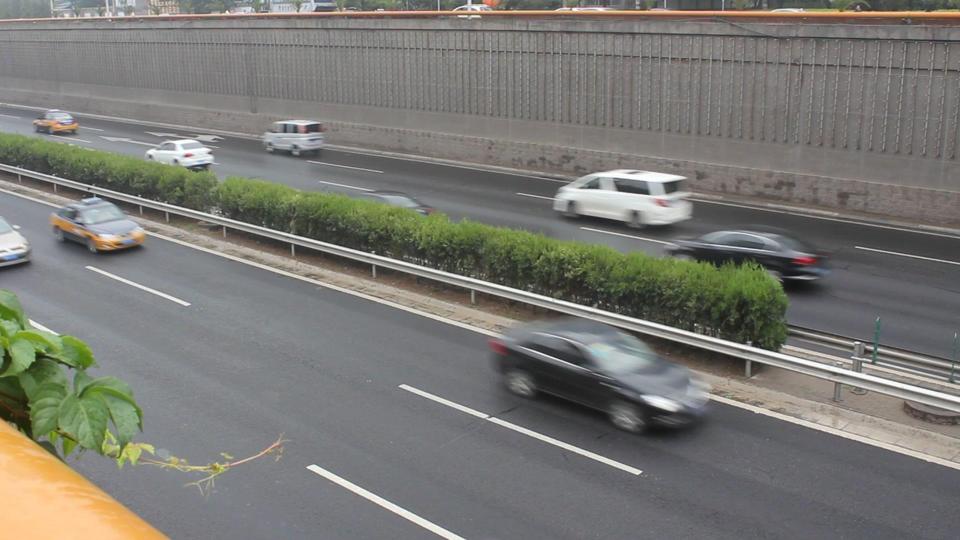

Supplement: Data S1 [file peerj-cs-09-1411-s001.zip › dataset/MVI_63552_img00854.jpg]

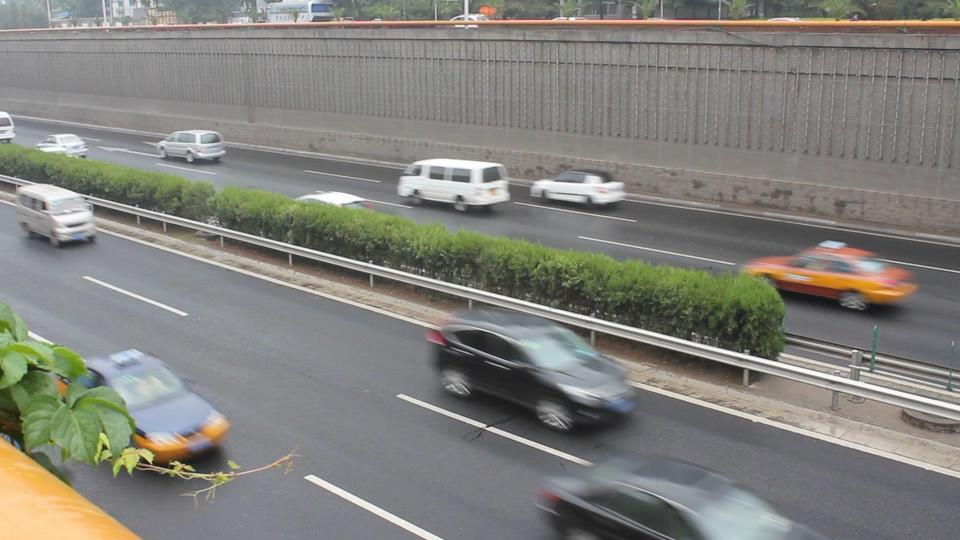

Supplement: Data S1 [file peerj-cs-09-1411-s001.zip › dataset/MVI_63552_img00698.jpg]

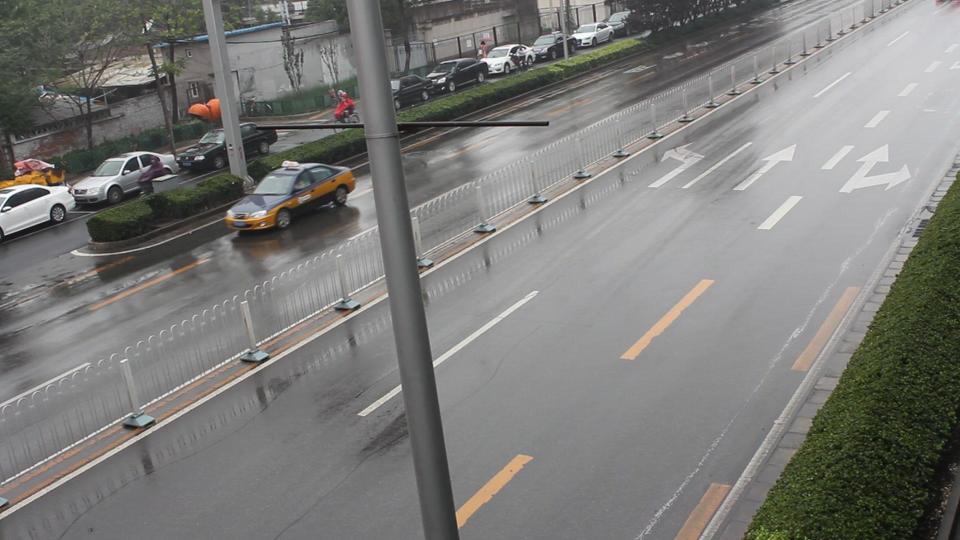

Supplement: Data S1 [file peerj-cs-09-1411-s001.zip › dataset/MVI_63544_img00727.jpg]

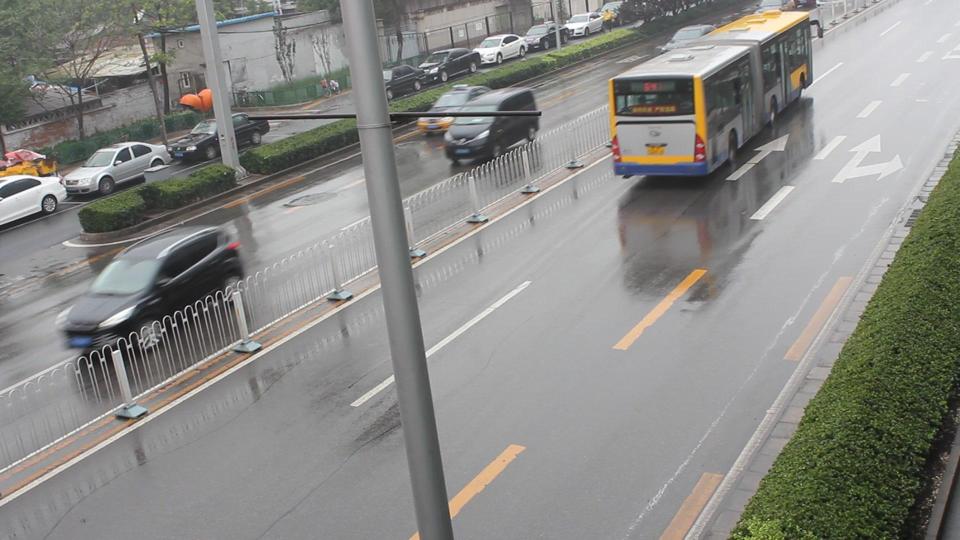

Supplement: Data S1 [file peerj-cs-09-1411-s001.zip › dataset/MVI_63544_img00041.jpg]

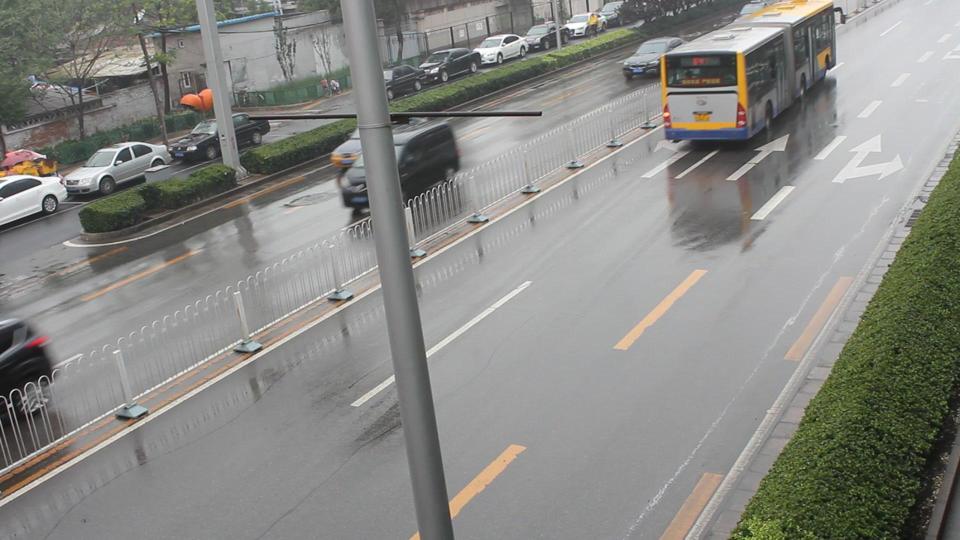

Supplement: Data S1 [file peerj-cs-09-1411-s001.zip › dataset/MVI_63544_img00055.jpg]

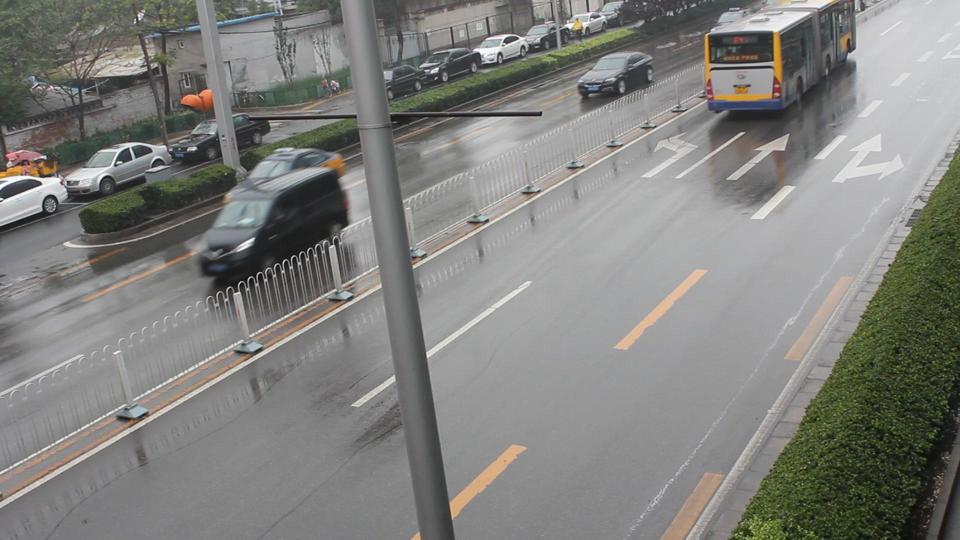

Supplement: Data S1 [file peerj-cs-09-1411-s001.zip › dataset/MVI_63544_img00069.jpg]
